# Methods for significance testing of categorical covariates in logistic regression models after multiple imputation: power and applicability analysis

**DOI:** 10.1186/s12874-017-0404-7

**Published:** 2017-08-22

**Authors:** Iris Eekhout, Mark A. van de Wiel, Martijn W. Heymans

**Affiliations:** 1 Department of Epidemiology & Biostatistics, VU University Medical Center, Amsterdam, The Netherlands; 2 Amsterdam Public Health research institute, VU University Medical Center, Amsterdam, The Netherlands; 3 Department Child Health, The Netherlands Organization of Applied Sciences (TNO), Schipholweg 77-89, 2316 ZL Leiden, The Netherlands; 4 Department of Mathematics, VU University, Amsterdam, The Netherlands

**Keywords:** Multiple imputation, Pooling, Categorical covariates, Significance test, Logistic regression, Simulation study

## Abstract

**Background:**

Multiple imputation is a recommended method to handle missing data. For significance testing after multiple imputation, Rubin’s Rules (RR) are easily applied to pool parameter estimates. In a logistic regression model, to consider whether a categorical covariate with more than two levels significantly contributes to the model, different methods are available. For example pooling chi-square tests with multiple degrees of freedom, pooling likelihood ratio test statistics, and pooling based on the covariance matrix of the regression model. These methods are more complex than RR and are not available in all mainstream statistical software packages. In addition, they do not always obtain optimal power levels. We argue that the median of the *p*-values from the overall significance tests from the analyses on the imputed datasets can be used as an alternative pooling rule for categorical variables. The aim of the current study is to compare different methods to test a categorical variable for significance after multiple imputation on applicability and power.

**Methods:**

In a large simulation study, we demonstrated the control of the type I error and power levels of different pooling methods for categorical variables.

**Results:**

This simulation study showed that for non-significant categorical covariates the type I error is controlled and the statistical power of the median pooling rule was at least equal to current multiple parameter tests. An empirical data example showed similar results.

**Conclusions:**

It can therefore be concluded that using the median of the *p*-values from the imputed data analyses is an attractive and easy to use alternative method for significance testing of categorical variables.

**Electronic supplementary material:**

The online version of this article (doi:10.1186/s12874-017-0404-7) contains supplementary material, which is available to authorized users.

## Background

Logistic regression modelling is a frequently applied method in epidemiological and medical studies. Although researchers try to avoid it, missing data occurs in all kinds of different study designs, and inevitably, also when logistic regression modelling is used. There are many different methods available to handle incomplete data [1, 2]. The most recommended method is multiple imputation (MI). MI is currently implemented in almost all statistical software packages and therefore within reach of many researchers. Hence, it will likely be applied more often. MI generates multiple imputed datasets, where after complete data analysis can be applied to each imputed dataset. Finally, parameter estimates can be combined using Rubin’s Rules (RR) [3].

For logistic regression modelling in combination with MI, the pooled regression coefficients and standard errors can easily be obtained by using RR. The pooled coefficient is derived by averaging the regression coefficient estimates from each complete data analysis result across the imputed datasets. The standard error is obtained by pooling the between imputation variance and the within imputation variance which account for sampling and imputation uncertainty, respectively. The pooled standard error is used to calculate 95% confidence intervals. For dichotomous and continuous covariates in a logistic regression model after MI, RR can easily be applied in combination with a single Wald statistic to obtain a *p*-value for significance [4].

To consider whether a categorical variable with more than two levels as a whole significantly contributes to the model, the methods to derive a pooled *p*-value are less straightforward. One method that can be used is to combine multiple chi-square values that result from a multiple parameter Wald or likelihood ratio test in each imputed dataset [5]. Alternatively, the pooled multivariate sampling variances of the regression model can be used to conduct a test that resamples a multivariate Wald statistic [6]. Meng and Rubin proposed another method in which the likelihood ratio test statistics are combined to provide a pooled *p*-value [7]. Unfortunately, none of these pooling methods are available in mainstream statistical packages. Consequently, the application of these methods may be complex for epidemiologists or other applied researchers, especially the Meng and Rubin pooling method. Furthermore, earlier studies showed that these methods do not always obtain optimal power levels, which is important for significance testing [7]. For that reason, it may be tempting for researchers to fall back on naïve methods, i.e., single imputation procedures, which often result in incorrect parameter estimates for statistical testing [2].

Van de Wiel et al. introduced the median of *p*-values in a cross-validation setting for inferring differences in prediction accuracies [8]. This setting is comparable to MI, because prediction accuracy is first obtained from separate (but related) generated versions of the data and subsequently inferred from those stochastically dependent data sets. This method showed proven control of the type I error rate and also good power results in different simulated data situations. It may therefore be a potential attractive method for significance testing of categorical variables.

Until now, methods to derive a pooled *p*-value for significance testing of categorical variables in logistic regression models have never been compared for their control of the type I error rate and power levels in different epidemiological data situations. Therefore, the aim of this study is to compare different pooling methods for significance testing of categorical and also continuous covariates in a logistic regression model after multiple imputation. Specifically type I error control and power after MI in a large simulation study will be evaluated. Moreover the characteristics of the pooling methods are further evaluated in an empirical dataset. In the Multiple imputation section the procedure of multiple imputation is more extensively described. In the Statistical hypothesis testing of a variable after MI section the different pooling methods for statistical testing after MI are discussed and in the Simulation section a simulation study is described that compared the different methods for pooling *p*-values of categorical variables. The methods are applied to a clinical dataset in the Application section.

## Multiple imputation

Multiple imputation is an advanced method to handle missing data, commonly performed in three phases: imputation, complete data analysis and pooling. In the imputation phase the missing values are replaced with *m* sets of plausible values. These values are estimated from a series of regression models to generate a posterior predictive distribution of the missing values that is used to draw the imputed values from. Each variable can be modeled according to its own distribution, i.e., continuous variables are modeled with linear regression and dichotomous variables with logistic regression. Imputations are generated within several sequential iteration rounds or chains, referred to as Multivariate Imputation by Chained Equations (MICE) [9, 10].

In the complete data analysis phase each imputed dataset is analyzed separately. The analysis performed is the same method that would have been applied had the data been complete. Accordingly, the analysis phase results in *m* sets of complete data results. The complete data analysis results from each imputed dataset will differ, because the imputed datasets differ

### Rubin’s rules (RR)

After the analyses the results are combined using pooling by RR. For parameter estimates (e.g., regression coefficients), the combined estimate $\overline{\theta}$ is the average of the estimates from the imputed data analyses:

$$ \overline{\theta}=\frac{\sum_{j=1}^m{\theta}_j}{m} $$

The standard errors of the parameter estimates are combined by using the within-imputation variance and the between-imputation variance [11]. The within imputation variance *Var(* $\overline{\theta}$ *)*_*within*_ is the average variance from the imputed data analyses:

$$ Var{\left(\overline{\theta}\right)}_{within}=\frac{\sum_{j=1}^m Var\left({\theta}_j\right)}{m} $$

The between imputation variance *Var(* $\overline{\theta}$ *)*_*between*_ is the sum of the squared deviation of the parameter estimate of each imputed data analysis from the pooled parameter estimate weighted by *m-1*:

$$ Var{\left(\overline{\theta}\right)}_{between}=\frac{\sum_{j=1}^m{\left({\theta}_j-\overline{\theta}\right)}^2}{m-1} $$

The variance of the parameter estimates is then calculated by combining the within and between variance:

$$ Var\left(\overline{\theta}\right)= Var\left(\overline{\theta}\right) within+\left(1+\frac{1}{m}\right) Var\left(\overline{\theta}\right) between $$

## Statistical hypothesis testing of a variable after MI

For logistic regression analysis, statistical testing of covariates after MI can be performed by different methods. The methods to pool the statistical tests after MI will be elaborated below with the focus on testing whether a categorical variable as a whole significantly contributes to the model.

### Univariate testing

For two-sided hypothesis testing of single regression coefficients in a logistic regression model after MI the Wald statistic W can be calculated as follows:

$$ {W}_{single}=\frac{{\left(\overline{\theta}-{\theta}_0\right)}^2}{Var\left(\overline{\theta}\right)}, $$

where $\overline{\uptheta}$ and $\mathrm{Var}\left(\overline{\uptheta}\right)$ are the pooled coefficient and corresponding variance, respectively and θ_0_ is the value under the null hypothesis. *W*_*single*_ follows a chi-square distribution with 1 degree of freedom. After MI, the Wald statistic can be calculated from the RR pooled statistics, which makes this an easy to apply method for continuous variables.

### Multivariate testing

For categorical variables in the logistic regression model only the pooled statistics for each separate level of a categorical variable can be obtained by RR, not the overall statistic. RR requires access to the variance-covariance matrices. Accordingly, each category can be tested, but the categorical variable as a whole cannot be tested without adapting the method. The several different multivariate pooling methods are discussed below. The formulas for these methods can be found in detail in Additional file 1.

#### Multiple parameter Wald test (CHI pooling)

One possibility is to pool the chi-square values from the multiple parameter Wald or likelihood ratio tests with multiple degrees of freedom (CHI pooling) [5]. The multiple parameter values are obtained after applying the test to each imputed datasets separately.

#### The pooled sampling variance (VAR pooling) method

Alternatively, a combination of the pooled parameter estimates and the pooled sampling variances can be used to construct a test that resembles a multivariate Wald test (VAR pooling) [12]. This test pools within and between covariance matrices that are obtained in each imputed dataset and finally corrects the total parameter covariance matrix of the multivariate Wald test by including the average relative increase in variance to account for the missing data [13].

#### Meng and Rubin pooling (MR pooling)

Meng and Rubin proposed a method to test overall categorical variables indirectly based on the likelihood ratio test statistic (MR pooling) [7]. For each regression parameter, two nested models are fitted in each imputed dataset: one restricted model where the parameter is not included in the model and one full model where the parameter is included. The pooled likelihood ratio tests are then compared to obtain pooled *p*-values for each parameter. The MR pooling method requires fitting multiple models for each variable in the data, hence it is an indirect approach. This can be a very time-consuming process.

#### The median P rule (MPR)

For the median P rule one simply uses the median *p*-value of the significance tests conducted in each imputed dataset (MPR pooling). Hence, it depends on *p*-values only and not on the parameter estimates. The MPR was developed in a cross-validation setting for comparing predictive performances of two methods [8]. In that setting, multiple splits of the same data set into training and test sets render multiple dependent *p*-values. Various bounds for the type I error when using thresholds like median *P* < 0.05 were proven under a variety of assumptions. One of these is the multivariate normal null-distribution (MVN) for *p*-values that are transformed to a standard normal scale: then, median *P* < α implies a type I error smaller than α. In the imputation setting, dependence between the multiple *p*-values is caused by the fact that all imputed datasets share the same observed data. Therefore, the dependency is likely to be strong. In the remainder of the paper we will use the significance rule median *P* < α, which we refer to as “median P rule” (MPR). For a real dataset the underlying MVN assumption cannot be checked, because we observe only one instance of the *p*-value vector. In simulations, however, it can be checked using the asymptotic χ^2^(p) distribution for the observed Mahalanobis distance as computed from the *p*-values transformed to the standard normal scale. Fig. A1 in Additional file 1 shows the empirical distribution of Mahalanobis distance *d* together with the χ^2^(10) distribution for 10 imputations (which allows reliable estimation of the inverse covariance matrix, required for computing *d*). These are based on 1000 simulations (see Simulation Section). Indeed, we observe a good match between the two for both the imputed categorical and the imputed continuous variable. Hence, we conclude that at least in this setting the MVN assumption is reasonable.

The MPR was evaluated by using the *p*-values from the likelihood ratio test for multiple parameters for the categorical variables in the multivariable model. In addition, we performed extensive simulations to support the validity of the MPR (Simulation section) and we supply a bootstrapping scheme that allows anyone to check the appropriateness of this rule (and the aforementioned alternatives) for a given data set (Application section).

## Simulation

### Simulation design

To study the performance of the CHI, VAR, MR and MPR pooling methods after multiple imputation we conducted a simulation study. In this study, data was generated for 250 cases. The data contained one categorical variable with four categories (Factor1) and four continuous variables (Covar1 – Covar4). The categorical variable was first created as a continuous variable, and then categorized by the quartiles of the variable. The categorical variable and the four continuous variables were the covariates in a model for a dichotomous outcome. The predictors were related to the outcome by multiplying coefficient loadings with the data matrix, and the resulting predictor matrix was used to estimate the probability of the outcome using a log-normal transformation of the linear predictor. The categorical variable was coded in the matrix by three dummy variables.

To create a variety of settings the data characteristics were varied. The correlation between the variables was varied between 0.2, 0.4, 0.6 and 0.8. Furthermore we varied the relation of the variables with the outcome by adjusting the coefficient values (betas). The betas for the continuous variables were varied from 0 to 1 with steps of 0.1. The betas of the dummy variables were varied by drawing the coefficients from a normal distribution with mean zero and a variance that also varied from 0 to 1 with steps of 0.1. Hence, for each correlation variation, ten different coefficient situations were simulated for 1000 datasets. This resulted in 40 conditions with 1000 datasets in each of these conditions.

We created missing values in the categorical variable (i.e., Factor1) and in the first continuous variable (i.e., Covar1). The percentage of missing values in both variables was set to either 25% or 40%. Accordingly, we created 40 conditions with 25% missing data, and 40 conditions with 40% missing data. The missing data was related to the other continuous variables in the data in order to simulate a missing at random missing data situation [3]. Each dataset with missing data was then imputed by multiple imputation. The number of imputations was set to 100. The data were analyzed using a generalized linear model.

### Comparing methods

We compared the performance of five methods to pool the *p*-values of the variable tests. The first method that was used is RR. For the continuous variables this method is used by default in the MICE algorithm in R. However, for the categorical dummy variables, this method will produce three pooled *p*-values in our study; one for each dummy. So no overall *p*-value is obtained. The second method is MR pooling, the third method the chi-square test with multiple degrees of freedom (CHI pooling), the fourth method the multivariate sampling variance method (VAR pooling), and the fifth method the MPR, which pooled the *p*-values from the overall likelihood ratio test in each imputed dataset by taking the median.

For each of the simulated data conditions, the average type I errors and powers of all pooling methods were compared for the incomplete categorical variable and the incomplete continuous variable. We compared the results of the pooled *p*-values to the *p*-values from the complete data. Those ‘full data’ *p*-values were obtained by applying the generalized linear model to the simulated data without missing values, followed by computing the average type I error and power over the 1000 simulated data sets per condition.

Note that it has been shown that for the purpose of regression coefficient estimation, inclusion of the outcome variable in the imputation model (i.e., outcome-based imputation) is recommended [14]. However, for hypothesis testing, outcome-based imputation may lead to over-optimistic *p*-values, rendering the pooled test result as too liberal. In the simulation study we investigated this aspect of the imputation model extensively by comparing the performances of the pooling methods for outcome-based imputation with the results when the outcome was excluded from the imputation model.

### Description of results

Table 1 presents the type I error for each pooling method compared to the complete data type I error, which is considered as the full data type I error, in the simulated condition when the beta equaled zero and 25% missing data. For all existing pooling methods outcome-based imputation was used, whereas for the MPR, results are presented both with inclusion (MPR_in_) and without (MPR_out_). Table 1 shows that the type I errors for all existing pooling methods, and also for the MPR_out_, were close to the target in all simulation conditions. For the MPR_in_ method the type I error was sometimes too liberal. These findings are confirmed in the situation where 40% of the data is missing (Additional file 2, Table B1).

**Table 1:** Type I error for each pooling method for simulated data with beta equal to zero for 25% missing data in varying correlations between the variables where Factor1 is categorical and Covar1-Covar4 are continuous

Cor		Full data	RR	MR	CHI	VAR	MPR_in_	MPR_out_
0.2	Factor	0.057	^a^	0.019	0.024	0.018	0.065	0.038
	covar1	0.056	0.048	0.052	0.057	0.048	0.104	0.028
	covar2	0.056	0.056	0.057	0.057	0.056	0.061	0.059
	covar3	0.043	0.051	0.057	0.055	0.051	0.063	0.052
	covar4	0.070	0.058	0.061	0.063	0.058	0.070	0.052
0.4	Factor	0.057	^a^	0.020	0.026	0.025	0.065	0.035
	covar1	0.056	0.046	0.048	0.052	0.046	0.094	0.030
	covar2	0.056	0.051	0.054	0.054	0.051	0.060	0.056
	covar3	0.043	0.059	0.063	0.061	0.059	0.070	0.052
	covar4	0.070	0.056	0.057	0.056	0.056	0.057	0.055
0.6	Factor	0.061	^a^	0.026	0.026	0.026	0.068	0.023
	covar1	0.058	0.048	0.049	0.051	0.048	0.088	0.031
	covar2	0.065	0.055	0.057	0.060	0.055	0.066	0.056
	covar3	0.059	0.051	0.054	0.053	0.051	0.058	0.051
	covar4	0.063	0.063	0.066	0.066	0.063	0.075	0.064
0.8	Factor	0.057	^a^	0.026	0.026	0.025	0.077	0.033
	covar1	0.056	0.057	0.058	0.058	0.057	0.098	0.019
	covar2	0.056	0.058	0.060	0.061	0.058	0.063	0.055
	covar3	0.043	0.060	0.061	0.061	0.060	0.070	0.043
	covar4	0.070	0.053	0.052	0.054	0.053	0.062	0.059

^a^For the categorical variable the *p*-value could not be obtained by RR; *Cor* correlation between variables; *Full data* complete data; *RR* Rubin’s Rules, *MR* Meng and Rubin pooling, *CHI* chi-square test with multiple degrees of freedom, *VAR* pooled sampling variance method, *MPR*_*in*_ Median P Rule with the outcome included in model, *MPR*_*out*_ Median P Rule with the outcome excluded from model

The power for each of the pooling methods was evaluated for the categorical and continuous variable after imputation of the missing data. Note that the standard errors of the estimated type I error/power, denoted by $\widehat{p}$, equal $sd\left(\widehat{p}\right)=\sqrt{\widehat{p}\left(1-\widehat{p}\right)/1000}$, which equals 0.007 for target type I error $\widehat{p}$ = 0.05 and which has its maximum for $\widehat{p}$ =0.5, rendering 0.015. Given that these standard errors are relatively small we have not displayed these in the Figures. Results are presented separately for outcome-based imputation and imputation where the outcome was excluded from the imputation model. Lastly, we present a summary result where the best strategy for each method is depicted.

#### Outcome-based imputation

**Fig. 1:**
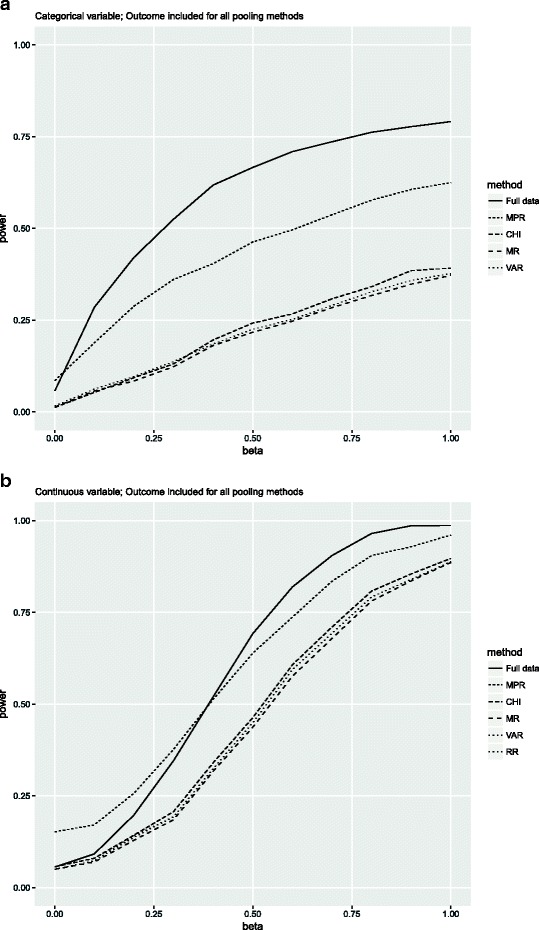
Power for the condition where the percentage of missing data was 25% and the correlation between the variables was 0.4 and the outcome was included in the imputation model. Note that for the continuous variable the lines for RR and VAR overlap. Full data = complete data; MPR = median P rule; CHI = chi-square test with multiple degrees of freedom; MR = Meng and Rubin pooling; VAR = pooled sampling variance method; RR = Rubin’s Rules

Figure 1 depicts the power for each pooling method after outcome-based imputation for the condition of 25% missing data and a correlation of 0.4, for each simulated coefficient value (beta). Figure 1 shows that for the categorical variable, the power for the CHI, MR and VAR pooling methods was smaller than for the full data situation. The power for the MPR was closer to the full data power. This occurs for all beta values in all data conditions, however as was concluded from Table 1, the type I error for MPR was slightly inflated. For the continuous variable, the MPR results in inflated power levels compared with the full data and the other pooling methods up to a beta value of 0.3. For beta values beyond 0.3 the power for MPR was larger compared to the other methods and closer to the full data power. The conditions with 40% missing data resulted in larger differences between the MPR, CHI, MR, VAR and RR pooling methods than in the 25% missing data conditions (see Additional file 2 for a full overview of the results).

#### Outcome excluded from imputation model

**Fig. 2:**
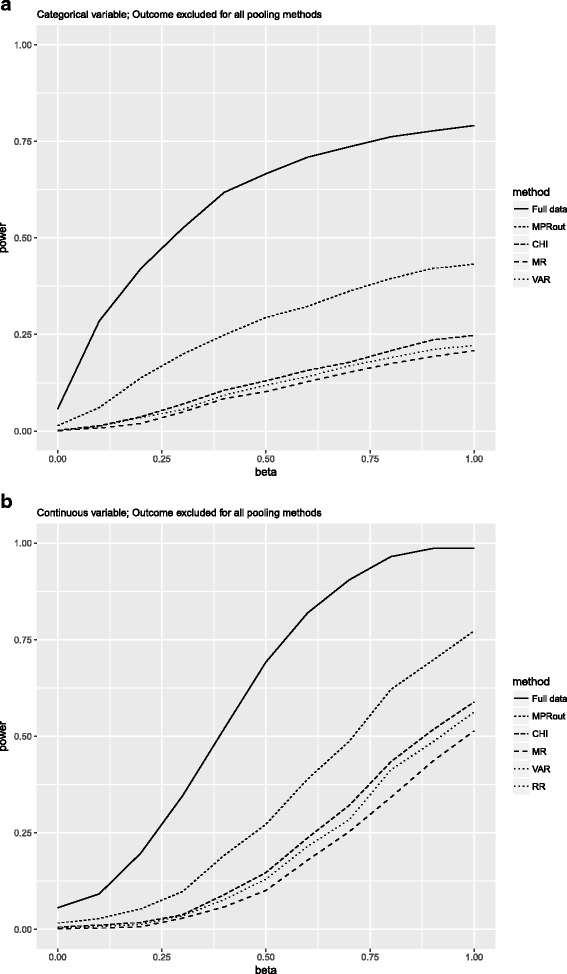
Power for the condition where the percentage of missing data was 25% and the correlation between the variables was 0.4 and the outcome was excluded from the imputation model. Note that for the continuous variable the lines for RR and VAR overlap. Full data = complete data; MPR = median P rule; CHI = chi-square test with multiple degrees of freedom; MR = Meng and Rubin pooling; VAR = pooled sampling variance method; RR = Rubin’s Rules

Figure 2 depicts the power for each pooling method with the outcome excluded from the imputation model for the condition of 25% missing data and a correlation of 0.4, for each simulated coefficient value (beta). Figure 2 shows that for the categorical variable, the power of the CHI, MR and VAR pooling methods is smaller than that of both the full data analysis as of the MPR method. This is also the case for the continuous variable as well as for the situation with 40% missing data (see Additional file 2 for a full overview of the results).

#### Summary: Outcome-based imputation except for MPR

**Fig. 3:**
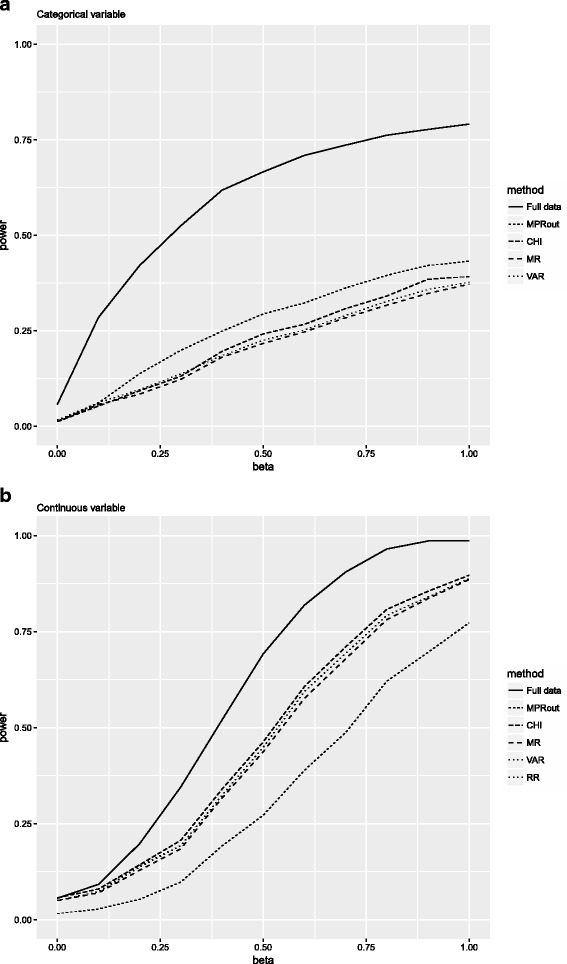
Power for the condition where the percentage of missing data was 25% and the correlation between the variables was 0.4. Note that for the continuous variable the *lines* for RR and VAR overlap. Full data = complete data; MPR_out_ = median P rule with outcome excluded from imputation model; CHI = chi-square test with multiple degrees of freedom; MR = Meng and Rubin pooling; VAR = pooled sampling variance method; RR = Rubin’s Rules

Figure 3 displays the results of the power analysis when for the RR, CHI, MR and the VAR pooling methods outcome-based imputation was applied, as recommended, and for the MPR when the outcome is excluded for imputation (MPR_out_). It is clear from these figures that the power for the MPR_out_ method for categorical variables is higher than for all other pooling methods and closer to the full data results at all beta values. Opposite results are found for the continuous variables where RR, CHI, MR and the VAR pooling methods show better power results. The results for 40% missing data and correlations of 0.2, 0.6 and 0.8 confirm these findings with larger differences in power levels between the MPR_out_ and the other pooling methods (see Additional file 2).

## Application

To illustrate our methods we used an example dataset adapted from a study about low back pain. The study population consisted of 299 workers that were listed as sick for a period of three weeks due to low back pain. Three treatment groups, high-intensity back school, low-intensity back schools and usual treatment by the occupational physician, were compared in a randomized clinical trial. The results for the short-term effects were published previously [15]. The primary outcome was the difference in pain after three months, measured with a dichotomous variable indicating a difference of at least three points. Possible continuous predictors for the outcome were age, body mass index (BMI), pain at baseline, physical functioning, disability, and kinesiophobia. Categorical predictors were treatment group (three categories), gender (two categories), education level (five categories), and some work-related physical variables, which were daily exposure to sitting (four categories), heavy lifting (four categories), and working with vibration tools (four categories). The original data contained missing values: 6% for BMI; 5.4% for pain at baseline; 25.5% for physical functioning; 6% for kinesiophobia; 7.8% for educational level; 5.7% for sitting; 5.4% for heavy lifting; and 10% for working with vibration tools. We started with a complete data situation by imputing the missing values once for the purpose of this illustration. This complete dataset was used as a true reference and a multivariable logistic regression model was fitted to this data. Model estimates including the corresponding *p*-values for this complete dataset are depicted in Table 2.

**Table 2:** Model estimates of complete data analysis

	Estimate	SE	Z	*p*-value
Intercept	−8.0215	2.4064	−3.3333	0.0008
Group				0.0516
Group (1)^a^	0.7535	0.3287	2.2927	0.0219
Group (2)^a^	0.5986	0.3338	1.7936	0.0729
Age	−0.0007	0.0141	−0.0498	0.9602
Gender	0.4247	0.3525	1.2049	0.2282
BMI	0.0619	0.0352	1.7587	0.0786
Education				0.5108
Education (1)^a^	−0.1501	0.4058	−0.3699	0.7114
Education (2)^a^	−0.3208	0.4397	−0.7297	0.4656
Education (3)^a^	−0.5997	0.6829	−0.8782	0.3798
Education (4)^a^	−1.2694	0.8117	−1.5639	0.1179
Sitting				0.0195
Sitting (1)^a^	0.6305	0.3295	1.9134	0.0557
Sitting (2)^a^	−0.3515	0.4795	−0.7329	0.4636
Sitting (3)^a^	1.0407	0.5286	1.9690	0.0489
Lifting				0.9830
Lifting (1)^a^	0.1441	0.3692	0.3903	0.6963
Lifting (2)^a^	0.0574	0.4127	0.1389	0.8894
Lifting (3)^a^	0.0990	0.4424	0.2238	0.8229
Vibration tools				0.0090
Vibration tools (1)^a^	−0.5406	0.3717	−1.4543	0.1459
Vibration tools (2)^a^	0.0554	0.4165	0.1329	0.8942
Vibration tools (3)^a^	−1.6335	0.5573	−2.9313	0.0034
Pain at baseline	0.3232	0.0836	3.8656	0.0001
Physical functioning	0.3220	0.1919	1.6778	0.0934
Disability	−0.9110	0.3283	−2.7747	0.0055
Kinesiophobia	0.0299	0.0222	1.3524	0.1762

^a^The numbers between brackets indicate the dummy variables; *SE* Standard Error

Missing values were generated in categorical and continuous variables by the missing at random mechanism, so the probability for missing data was related to other variables in the data, to create a realistic data situation. About 25% of the cases had missing data on BMI, education, heavy lifting, and physical functioning. This incomplete dataset was imputed 100 times without including the outcome variable in the imputation model for MPR pooling of categorical variables (MPR_out_) and outcome-based imputation was used for the other variables and the RR, MR, CHI and VAR pooling methods. The variables without missing observations were included in the imputation model and multivariate imputation by chained equations was used to impute missing values. The same multivariable logistic regression model was fitted as for the complete data analysis on each imputed dataset. Subsequently, the *p*-values for each independent variable were pooled according to the four compared methods: RR, MR pooling, CHI pooling, VAR pooling and the MPR_out_. Listwise deletion was also applied and presented as comparison, where only the cases with completely observed data are included in the analysis. The resulting *p*-values are depicted in Table 3, along with the complete reference data *p*-values (‘Full data’) in the first column.

**Table 3:** *P*-values from complete data analysis, pooling methods and listwise deletion

	Full data	RR	MR	CHI	VAR	MPR_out_	Listwise
Group	0.0515	^a^	0.0498	0.0583	0.0643	0.0549	0.3234
Age	0.9602	0.9245	0.9283	0.8780	0.9244	0.8898	0.8245
Gender	0.2250	0.3040	0.2854	0.3017	0.3041	0.2862	0.8836
BMI	0.0764	0.0172	0.0222	0.0137	0.0173	0.0049	0.0103
Education	0.5108	^a^	0.7546	0.7235	0.7468	0.4579	0.6141
Sitting	0.0195	^a^	0.0396	0.0355	0.0498	0.0306	0.1196
Lifting	0.9830	^a^	0.9485	0.8755	0.9484	0.7605	0.9289
Vibration Tools	0.0090	^a^	0.0115	0.0130	0.0236	0.0109	0.0833
Pain baseline	0.0000	0.0001	0.0000	0.0000	0.0001	0.0000	0.0008
Physical Functioning	0.0913	0.0970	0.1095	0.0943	0.0970	0.0532	0.0608
Disability	0.0049	0.0032	0.0009	0.0027	0.0032	0.0022	0.0595
Kinesiophobia	0.1730	0.2115	0.2312	0.2084	0.2115	0.2018	0.0438

^a^For the categorical variables the overall *p*-value could not be obtained by RR. *Full data* complete data, *RR* Rubin’s Rules, *MR* Meng and Rubin pooling, *CHI* chi-square test with multiple degrees of freedom, *VAR* pooled sampling variance method; *MPR*_*out*_ Median P Rule with the outcome excluded from model, *Listwise* analysis after excluding cases with missings

In this example we observe that the MPR performed similar to the Meng and Rubin method, the chi-square pooling with multiple degrees of freedom and pooled sampling variance method. The smaller *p*-values were often closer to the *p*-values from the complete data analysis for the MPR than for MR, CHI and VAR. Furthermore, multiple imputation improved the results in particular for Group and Sitting when comparing the pooled *p*-values to the *p*-values after listwise deletion.

We use this data example to show how one can verify control of type I error and power for the various pooling methods using a *data-based* simulation. We used the estimated means and covariance matrix from our own data example to generate 1000 bootstrap samples of the covariates from a multivariate normal distribution. The sample size from the original data was used for the bootstrap samples (*n* = 299). Subsequently, we used the pooled coefficient estimates after MI from the analysis performed above to create the dichotomous outcome variable. Note that to obtain the coefficient estimates we used outcome-based imputation as recommended [14]. For simulating the outcome variable we only used the coefficients that were significantly contributing to the model by using the MPR *p*-values with a threshold of *P* < 0.05. So the outcome was predicted by BMI, sitting, vibration tools, pain at baseline and disability. Then missing data were generated according to the pattern that was used in the example above and MI was applied with the different methods to pool the *p*-values.

Table 4 presents the probability for each variable to obtain a significant result (*P* < 0.05). From the variables above the dashed line (that are present in the model), we conclude that the power of MPR is larger compared to RR, MR, CHI and VAR pooling. For example, we observe that the power for the detection of the association between the variable ‘Disability’ and the outcome was about 21% higher for MPR than for MR pooling. From the variables below the dashed line (not present in the model) we observe that the type I error of MPR is generally more on target than that of the other methods, although MPR was for some variables slightly anti-conservative with a type I error slightly over 0.05, which is not very problematic in practice. These findings are in concordance with those of our simulation study.

**Table 4:**
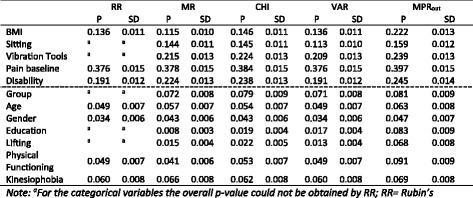
Probability (P) and standard deviation (SD) for rejection of the null-hypothesis, i.e. power for variables above dashed line and type I error for variables below dashed line, from data based simulation

^a^For the categorical variables the overall *p*-value could not be obtained by RR; *RR* Rubin’s Rules, *MR* Meng and Rubin pooling, *CHI* chi-square test with multiple degrees of freedom, *VAR* pooled sampling variance method, *MPR*_*out*_ Median P Rule with the outcome excluded from model

## Discussion

Multiple imputation is a frequently used method when covariate data is missing in prognostic models. In the process of defining the prognostic model one is most interested in the overall test to make a decision on the relevance of the categorical variable as a whole for the model. However, such a test is not easily applied in the case of MI and asks for (complex) adjustments in the pooling process or for switching between different software packages. If these packages are not available by the researcher, the researcher may fall back to simple but erroneously single imputation techniques. We showed in a simulation study that the MPR is an easy-to-use method for statistical testing of categorical variables in a multiple imputation context. The performance of the MPR is tested in many different data conditions and proved to be consistently satisfactory. In particular, when compared to alternative methods which are pooling the chi-square values with multiple degrees of freedom, using the pooled sampling variances and the method proposed by Meng and Rubin [7], the MPR performs equally well and the resulting pooled *p*-value for the categorical variable is often more on target than the pooled *p*-values derived from the other methods.

To obtain a powerful significance test for continuous and dichotomous variables with RR after MI, the MI procedure has to include the outcome variable, as was also indicated by Moons et al. [16]. For overall significance testing of categorical variables by using the multiple parameter Wald test (Chi Pooling), the pooled sampling variance (VAR pooling) method and Meng and Rubin pooling (MR pooling), we suggest following this recommendation. It should be mentioned though that these results assume a correct imputation model, and establishing robustness against misspecification of this model requires further study. The imputation should be outcome-based for continuous variables that are pooled with RR, or when applying the more complex pooling methods (i.e., CHI pooling, VAR pooling or MR pooling), because for these methods the pooling parameters are estimates that are pooled after which the result of the hypothesis test is obtained. However, to obtain correct and powerful pooled *p*-values for significance testing of categorical variable as a whole with the MPR, the outcome should be omitted from the imputation model. Note that omitting the outcome for imputation may have a robustness advantage, because such imputation does not assume a specific model for the relation between outcome and covariates. In MPR, the hypothesis test results are directly pooled; the pooling parameters are the *p*-values. In this case, using the outcome in the imputation model would lead to over-optimistic hypothesis test results, as was shown in the simulation. What are the practical consequences of our results? We suggest the following guideline: If the continuous variable(s) are most important: use one of the available pooling after outcome-based imputation methods. If the categorical variable(s) are of primary interest: use MPR with outcome-excluded imputation. In both cases, both procedures render valid results for the other variables as well (in terms of type I error control), but may lack power. Hence, if *p*-values for the other type of variables are just above 0.05, we recommend applying the alternative procedure to gain power. This comes at some computational cost, but, the MPR rule is very easy to apply in any software package that can perform MI and therefore time-saving in itself.

The usability of the pooling methods depends largely on their availability in statistical software. Software packages vary in methodology to pool parameters after MI. For example, In Stata and Mplus the multiple parameter pooling method (CHI pooling) can be used [17, 18]. There is separate SAS add-on code available for CHI pooling and a translated version was developed for R [19]. In Mplus, SAS and R the Meng and Rubin test (MR pooling) is available [20]. For R this procedure is available in the MICE package [9, 20]. The pooled sampling variance method is available in Mplus, SAS and R. The strength of the MPR rule is that this rule can easily be applied posterior to MI in any software package. This is a large advantage for the many researchers that are most familiar with the use of statistical software package SPSS. These researchers do not have to switch to other software programs for the MI procedure in order to pool *p*-values of categorical variables.

The parameters that are pooled with RR or in MR, CHI, and VAR pooling follow a normal distribution. To pool these parameters, the mean is used. The parameters that are pooled with MPR are *p*-values, which do not follow a normal distribution. For that reason, it is warranted to pool using the median instead of the mean. As described in the paper by Marshall et al. [4], for other parameters, such as the proportion of variance explained or discrimination indices, the median may be a good summary estimator after MI. In future research, the application of MPR can be explored in many other situations where there is not yet a widely available pooling method at hand. Examples are non-parametric testing of variables after MI such as the pooled *p*-values for spearman rho correlation coefficients. But also to pool *p*-values from the F-tests of an analysis of variance (ANOVA). Van Ginkel and Kroonenberg developed a method to pool the F-tests of an ANOVA, but this procedure is rather complicated and not available in all software packages [21]. Also situations where likelihood ratio test statistics have to be pooled, i.e. comparing multilevel models in multiply imputed datasets, may benefit from the application of a pooling procedure such as MPR.

## Conclusions

In conclusion, the MPR is an attractive rule for statistical inference of categorical variables with more than two levels because it has at least equal power as the multi parameter tests that are currently used but is much easier to apply in any software package.

## Additional files

Additional file 1: Formulas of the different multivariate pooling methods. (DOCX 85 kb)

Additional file 2: Full overview of simulation results. (PDF 81 kb)

## Abbreviations

ANOVA: Analysis of variance
BMI: Body mass index
CHI: Chi-square test with multiple degrees of freedom
MI: Multiple imputation
MICE: Multiple imputation by chained equations
MPR: Median P rule
MPR_out_: Median P rule with outcome excluded from imputation model
MR: Meng and Rubin pooling
RR: Rubin’s Rules
VAR: Pooled sampling variance method
