# Supplementary material for: Methods for significance testing of categorical covariates in logistic regression models after multiple imputation: power and applicability analysis
Source: BMC Med Res Methodol. 2017 Aug 22;17:129. doi: 10.1186/s12874-017-0404-7 (PMC5568368; doi:10.1186/s12874-017-0404-7)
Supplement: Supplementary file 1 — Formulas of the different multivariate pooling methods. (DOCX 85 kb) [file 12874_2017_404_MOESM1_ESM.docx]

**Appendix A.**

*Multiple parameter Wald test (CHI pooling)*

The following formula is used to obtain the chi-square values from a multiple parameter Wald test (Marshall, Altman, Holder, & Royston, 2009):

$W_{chi}=\left( 1+r \right)^{-1}\left( \frac{\bar{\omega}}{k}-\frac{m+1}{m-1} r \right)$ ,

where $\bar{\omega}$ is the mean of the chi-square values over the imputed datasets, $k$ is the degrees of freedom of the chi-square test statistic, $m$ is the number of imputed datasets and *r* reflects a measure of the relative increase in variance due to nonresponse (or fraction of missing information), which is obtained by the following formula:

$r= \frac{m+1}{m(m-1)}\sum_{j=1}^{m} ({\sqrt{\omega_{j}}-\sqrt{\bar{\omega}})}^{2}$ ,

with m and $\bar{\omega}$ as above, j = 1 …, m the index of each separate imputed dataset and $\omega_{j}$ is the chi-square value in each imputed dataset. The p-value is calculated by comparing the $W_{chi}$ statistic to an $F$distribution with $k$ and $v$ degrees of freedom as follows:

$$P=\Pr[F_{k,v}>W_{chi}]$$

The pooled sampling variance (VAR pooling) method

The multivariate Wald statistic is calculated as (Enders, 2010; Marshall et al., 2009):

$W_{mvar}=\frac{\left( 1+r_{1} \right)^{-1}(\theta_{0}-\bar{\theta})\bar{U}^{-1}{(\theta_{0}-\bar{\theta})}^{t}}{k}$ ,

where $\bar{\theta}$ and $\theta_{0}$are the pooled coefficient and the value under the null hypothesis, $\bar{U}$ is the within imputation variance (Var($\bar{\theta}$)_within_), $t$ is the total variance for the pooled estimate ($Var\left( \bar{\theta} \right)$), and k is the number of parameters. The $r_{1}$is the relative increase in variance due to nonresponse (fraction of missing information), which is in this case obtained by:

$r_{1}=\frac{\left( 1+m^{-1} \right)Tr({B\bar{U}}^{-1})}{k}$,

where $B$ is the between imputation variance (Var($\bar{\theta}$)_between_) and $m$ is the number of imputed datasets. The p-values is calculated by comparing the $W_{mvar}$ statistic to an $F$distribution with $k$ and $v_{1}$ degrees of freedom.

$$P=\Pr[F_{k,v_{1}}>W_{mvar}]$$

$v_{1}=4+\left( km-k-4 \right){[1+\left( 1-\frac{2}{km-k} \right)\frac{1}{r_{1}}]}^{2}$,

If $km-k<4$, the formula above applies, otherwise:

$v_{1}=\frac{(km-k)(1+\frac{1}{k}){(1+\frac{1}{r_{2}})}^{2}}{2}$.

*Meng and Rubin pooling (MR pooling)*

The Meng and Rubin pooling method works in the following steps (Meng & Rubin, 1992):

1. for each regression parameter θ two nested models are fitted in each imputed dataset: one where θ is included (full model) and one where θ is not included in the model (restricted model). Subsequently, these models are pooled to obtain $\bar{\theta}_{full}$ and $\bar{\theta}_{restricted}$.
2. The average likelihood ratio test statistic $\bar{d}$_L_ over the imputed datasets as a result of comparing the log likelihood values between these models is calculated as:

$$\bar{d}_{L}=\frac{1}{m}\sum_{j=1}^{m} 2\left( L_{restricted}-L_{full} \right),$$

where L_restricted_  and *L_full_* represent the maximum log likelihood values with respect to θ.

1. The log likelihood values from the two models of step 2 are then re-calculated and averaged using the model parameters $\bar{\theta}_{full}$ and $\bar{\theta}_{restricted}$ of step 1 (which were constrained to the values from the models in the imputed data):

$\bar{d}_{constrained}=\frac{1}{m}\sum_{j=1}^{m} 2\left( L\left( \bar{\theta}_{full} \right)-L\left( \bar{\theta}_{restricted} \right) \right)$,

1. The resulting test statistic D_L_ , required to obtain the pooled p-value, is calculated by incorporating the average increase in variance due to nonresponse $\bar{r}$_L_ as follows:

$D_{L}= \frac{\bar{d}_{constrained}}{k(1+\bar{r_{L}})}$,

$\bar{r_{L}}=\frac{m+1}{k\left( m-1 \right)}(\bar{d}_{L}-\bar{d}_{constrained})$,

where k is the number of degrees of freedom in the complete data likelihood ratio test (Mistler, 2013; van Buuren, 2012). The p-value is calculated by comparing the $D_{L}$ statistic with an F distribution with k and *v_L_*  (i.e., degrees of freedom of the denominator) according to:

$$P_{L}=\Pr[F_{k,v_{L}}>D_{L}]$$

$$v_{L}=4+(km-k-4)\left[ 1+(1-\frac{2}{km-k}\frac{1}{r_{L}} \right]^{2}$$

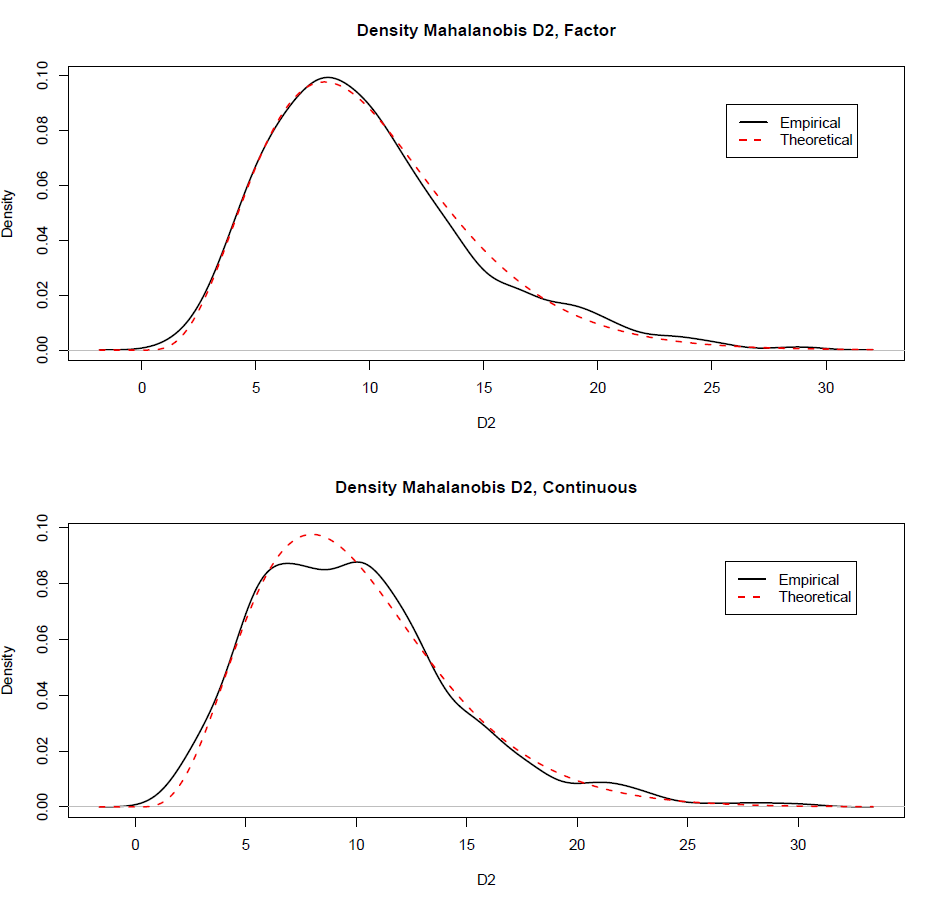


*Figure A1. The empirical distribution of Mahalanobis distance d together with the χ2(10) distribution for 10 imputations based on 1000 simulations*

**References**

Enders, C. K. (2010). *Applied Missing Data Analysis*. (T. D. Little, Ed.)*Methodology in the Social Sciences*. New York, NY: The Guilford Press.

Marshall, A., Altman, D. G., Holder, R. L., & Royston, P. (2009). Combining estimates of interest in prognostic modelling studies after multiple imputation: current practice and guidelines. *BMC Med Res Methodol*, *9*, 57. http://doi.org/10.1186/1471-2288-9-57

Meng, X.-L., & Rubin, D. B. (1992). Performing Likelihood Ratio Tests with Multiply-Imputed Data Sets. *Biometrika*, *79*(1), 103–111. Retrieved from http://www.jstor.org/stable/2337151

Mistler, S. A. (2013). A SAS® Macro for Computing Pooled Likelihood Ratio Tests with Multiply Imputed Data. *Statistical and Data Analysis*. San Francisco, California: Contributed Paper : SAS Global Forum 2013.

Van Buuren, S. (2012). *Flexible Imputation of Missing data*. (N. Keiding, B. J. T. Moragan, C. K. Wikle, & P. van der Heijden, Eds.)*Interdisciplinary Statistics Series*. New York: Chapman & Hall/CRC.
