# Supplementary material for: Methods for significance testing of categorical covariates in logistic regression models after multiple imputation: power and applicability analysis
Source: BMC Med Res Methodol. 2017 Aug 22;17:129. doi: 10.1186/s12874-017-0404-7 (PMC5568368; doi:10.1186/s12874-017-0404-7)

Table B1a. Type I error with beta equaling zero.40% missing data & Cor = 0.2

	True	RR	MR	CHI	VAR	MPRout	MPRin
**Factor**	0.057	0.069	0.014	0.015	0.015	0.02	0.088
**covar1**	0.056	0.044	0.041	0.048	0.044	0.02	0.123
**covar2**	0.056	0.058	0.058	0.06	0.058	0.053	0.066
**covar3**	0.043	0.065	0.063	0.068	0.065	0.046	0.082
**covar4**	0.07	0.048	0.049	0.048	0.048	0.054	0.058

Table B1b. Type I error with beta equaling zero.40% missing data & Cor = 0.4

	True	RR	MR	CHI	VAR	MPRout	MPRin
**Factor**	0.057	0.072	0.013	0.012	0.016	0.014	0.085
**covar1**	0.056	0.05	0.05	0.057	0.05	0.016	0.152
**covar2**	0.056	0.057	0.058	0.06	0.057	0.059	0.062
**covar3**	0.043	0.063	0.066	0.066	0.063	0.046	0.074
**covar4**	0.07	0.051	0.055	0.055	0.051	0.048	0.064

Table B1c. Type I error with beta equaling zero.40% missing data & Cor = 0.6

	True	RR	MR	CHI	VAR	MPRout	MPRin
**Factor**	0.061	0.075	0.02	0.019	0.021	0.014	0.083
**covar1**	0.058	0.038	0.037	0.043	0.038	0.018	0.12
**covar2**	0.065	0.05	0.051	0.054	0.05	0.053	0.066
**covar3**	0.059	0.042	0.044	0.047	0.042	0.039	0.051
**covar4**	0.063	0.067	0.064	0.071	0.067	0.067	0.085

Table B1d. Type I error with beta equaling zero.40% missing data & Cor = 0.8

	True	RR	MR	CHI	VAR	MPRout	MPRin
**Factor**	0.057	0.074	0.014	0.016	0.015	0.014	0.098
**covar1**	0.056	0.05	0.044	0.053	0.049	0.014	0.127
**covar2**	0.056	0.055	0.06	0.057	0.055	0.054	0.061
**covar3**	0.043	0.063	0.064	0.067	0.063	0.044	0.074
**covar4**	0.07	0.049	0.053	0.053	0.049	0.055	0.064

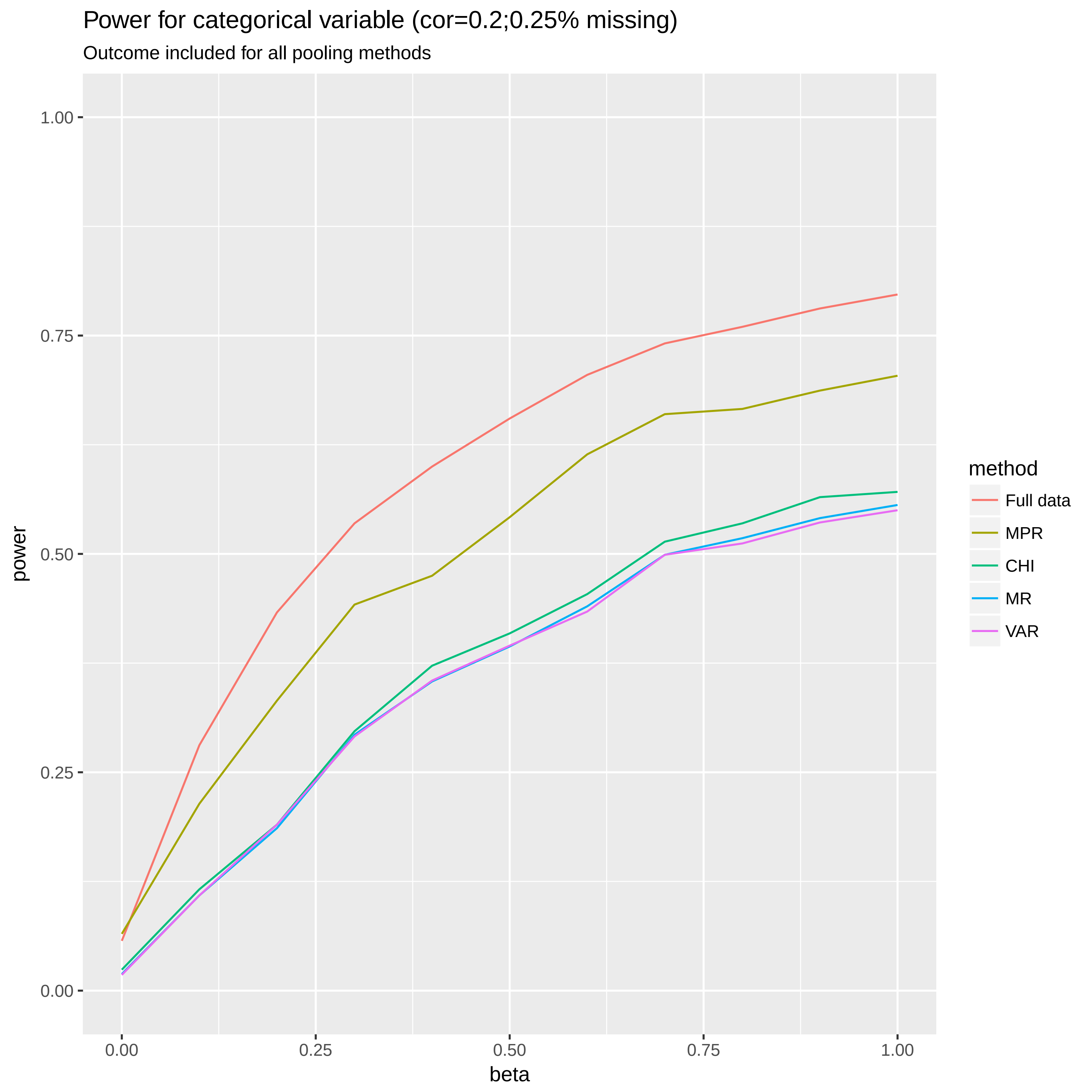

Power for categorical variable (cor=0.2;0.25% missing)
Outcome included for all pooling methods
power
beta
0.00
0.25
0.50
0.75
1.00
method
Full data
MPR
CHI
MR
VAR

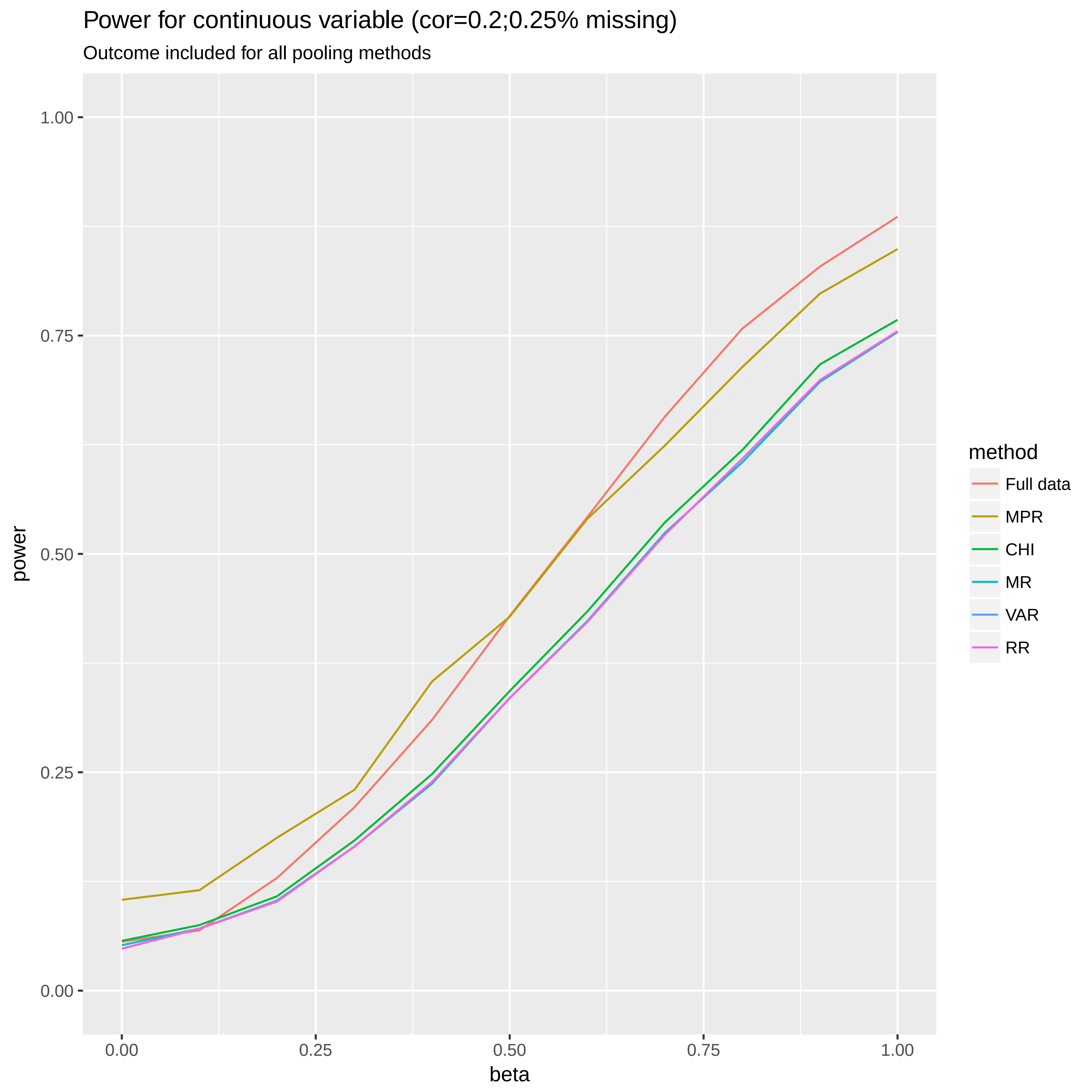
Power for continuous variable (cor=0.2;0.25% missing)
Outcome included for all pooling methods
power
1.00
0.75
0.50
0.25
0.00
beta
0.00
0.25
0.50
0.75
1.00
method
Full data
MPR
CHI
MR
VAR
RR

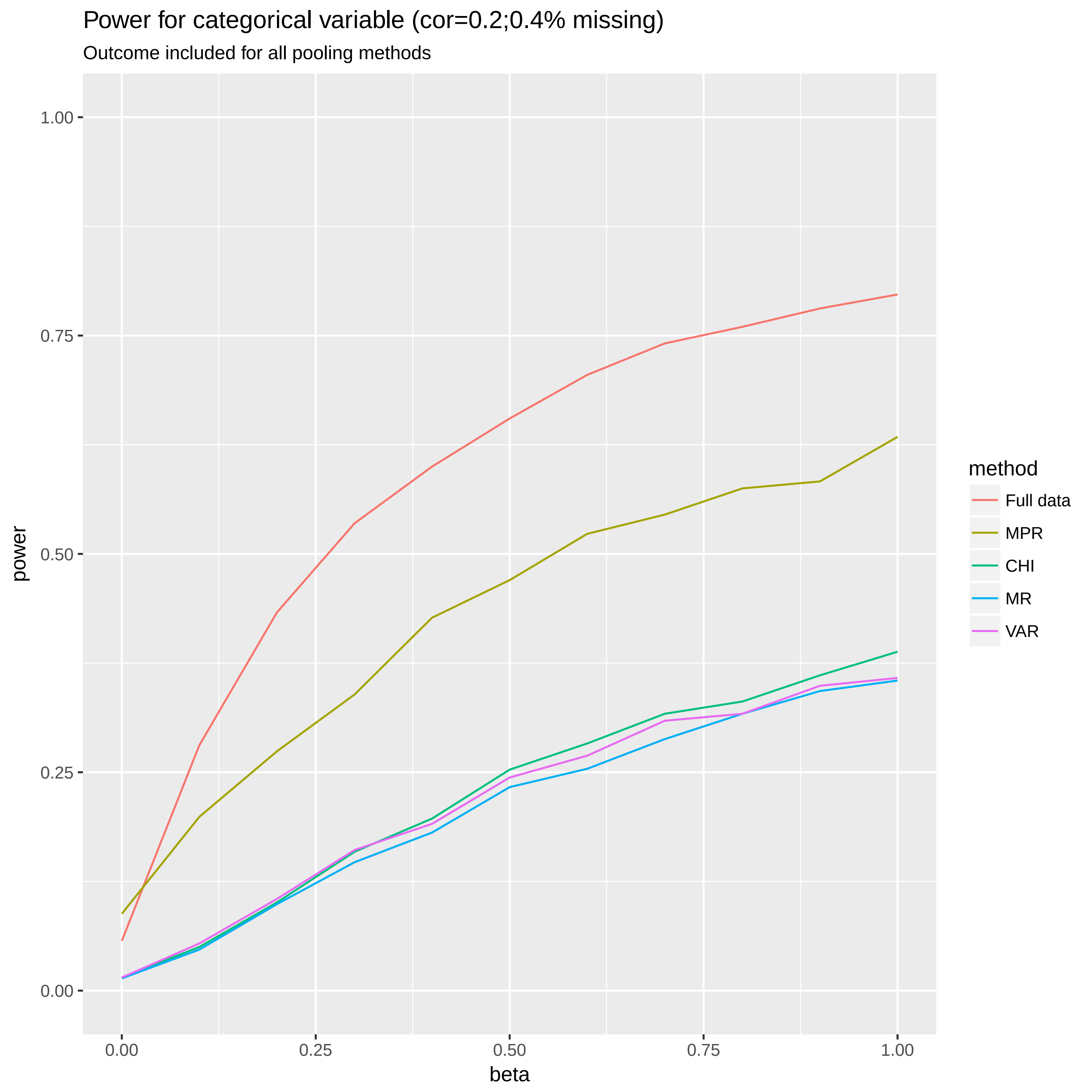

Power for categorical variable (cor=0.2;0.4% missing)
Outcome included for all pooling methods
power
beta
1.00
0.75
0.50
0.25
0.00
0.00
0.25
0.50
0.75
1.00
method
Full data
MPR
CHI
MR
VAR

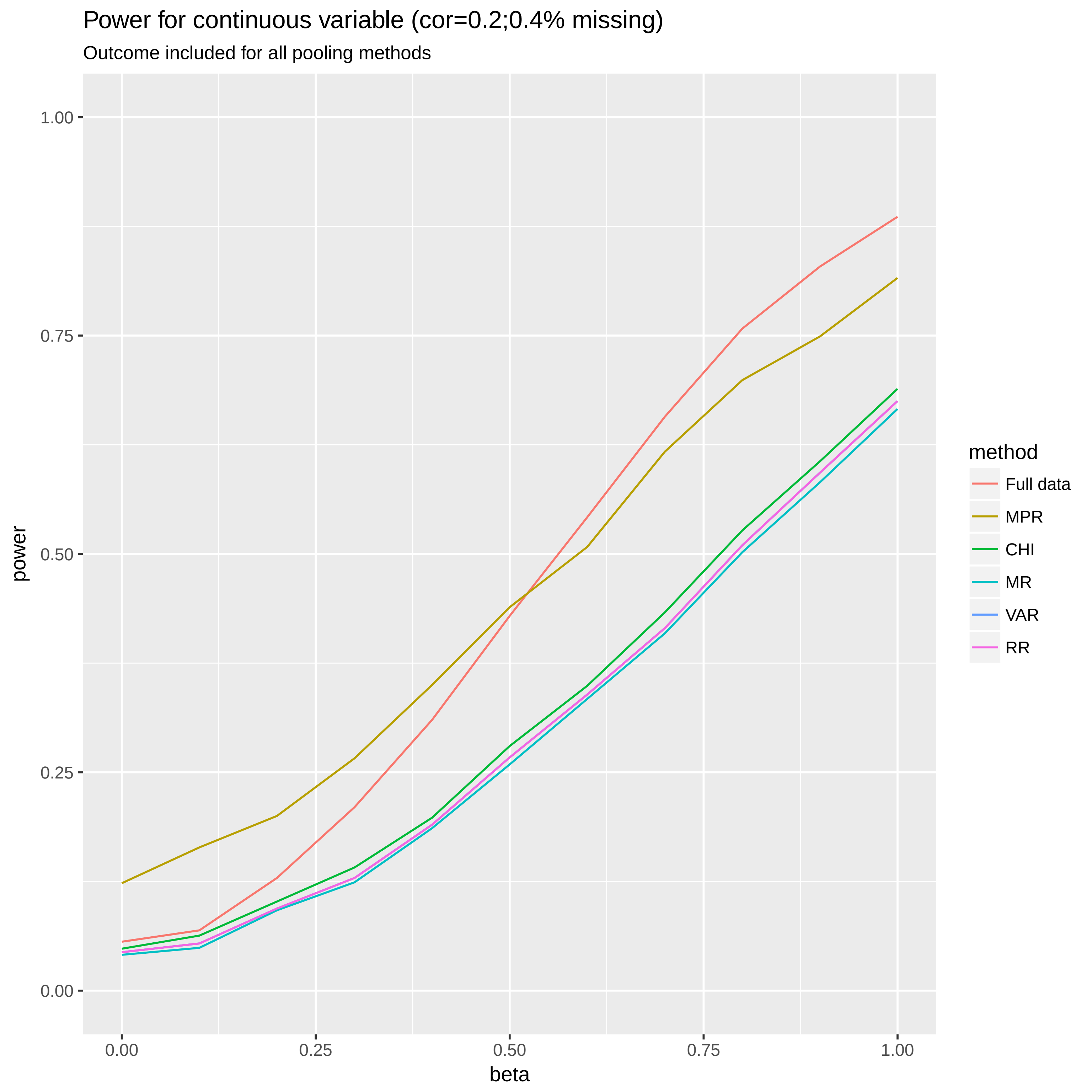

Power for continuous variable (cor=0.2;0.4% missing)
Outcome included for all pooling methods
power
1.00
0.75
0.50
0.25
0.00
beta
0.00
0.25
0.50
0.75
1.00
method
Full data
MPR
CHI
MR
VAR
RR

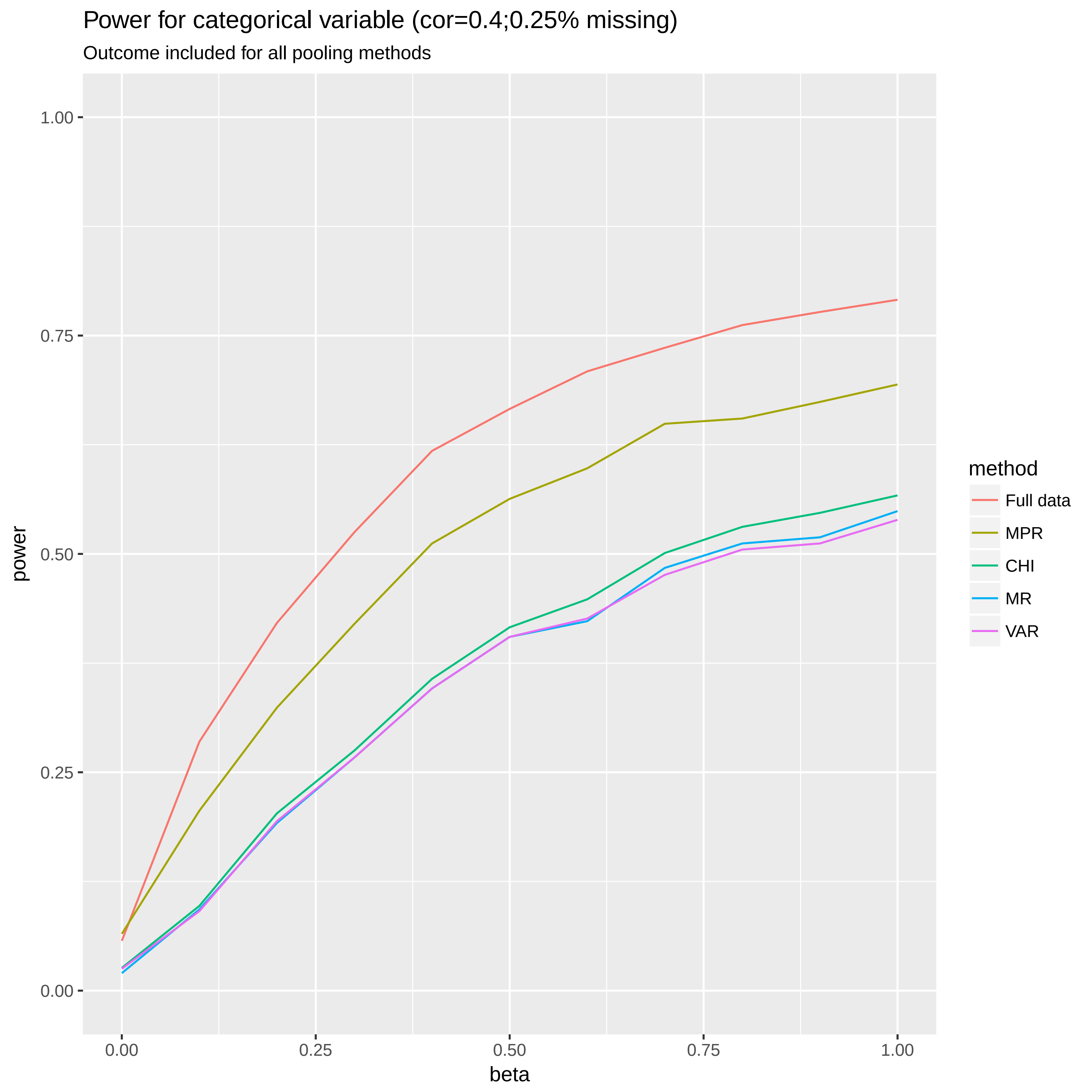

Power for categorical variable (cor=0.4;0.25% missing)
Outcome included for all pooling methods
power
1.00
0.75
0.50
0.25
0.00
beta
0.00
0.25
0.50
0.75
1.00
method
Full data
MPR
CHI
MR
VAR

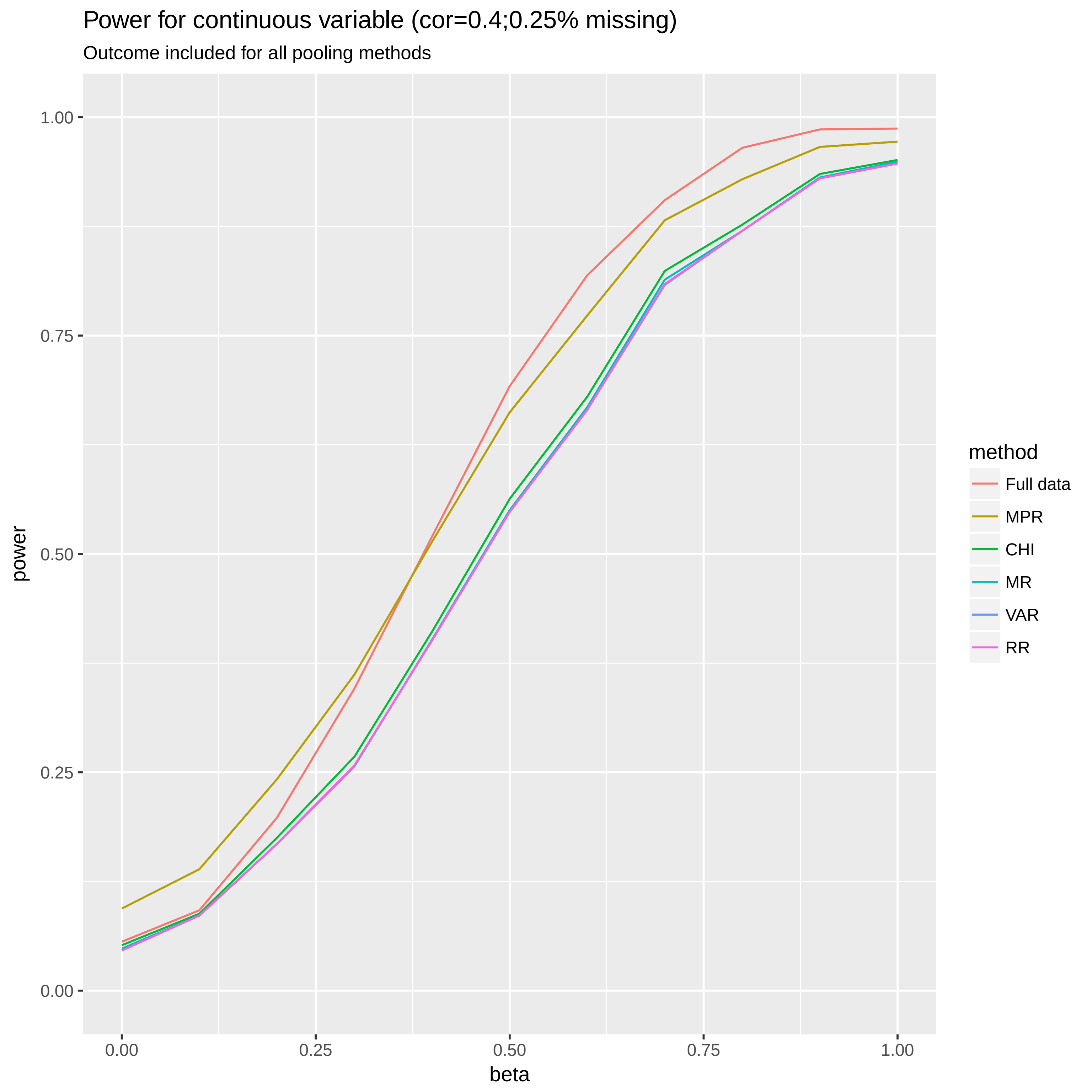

Power for continuous variable (cor=0.4;0.25% missing)
Outcome included for all pooling methods
power
1.00
0.75
0.50
0.25
0.00
0.00
0.25
0.50
0.75
1.00
beta
method
Full data
MPR
CHI
MR
VAR
RR

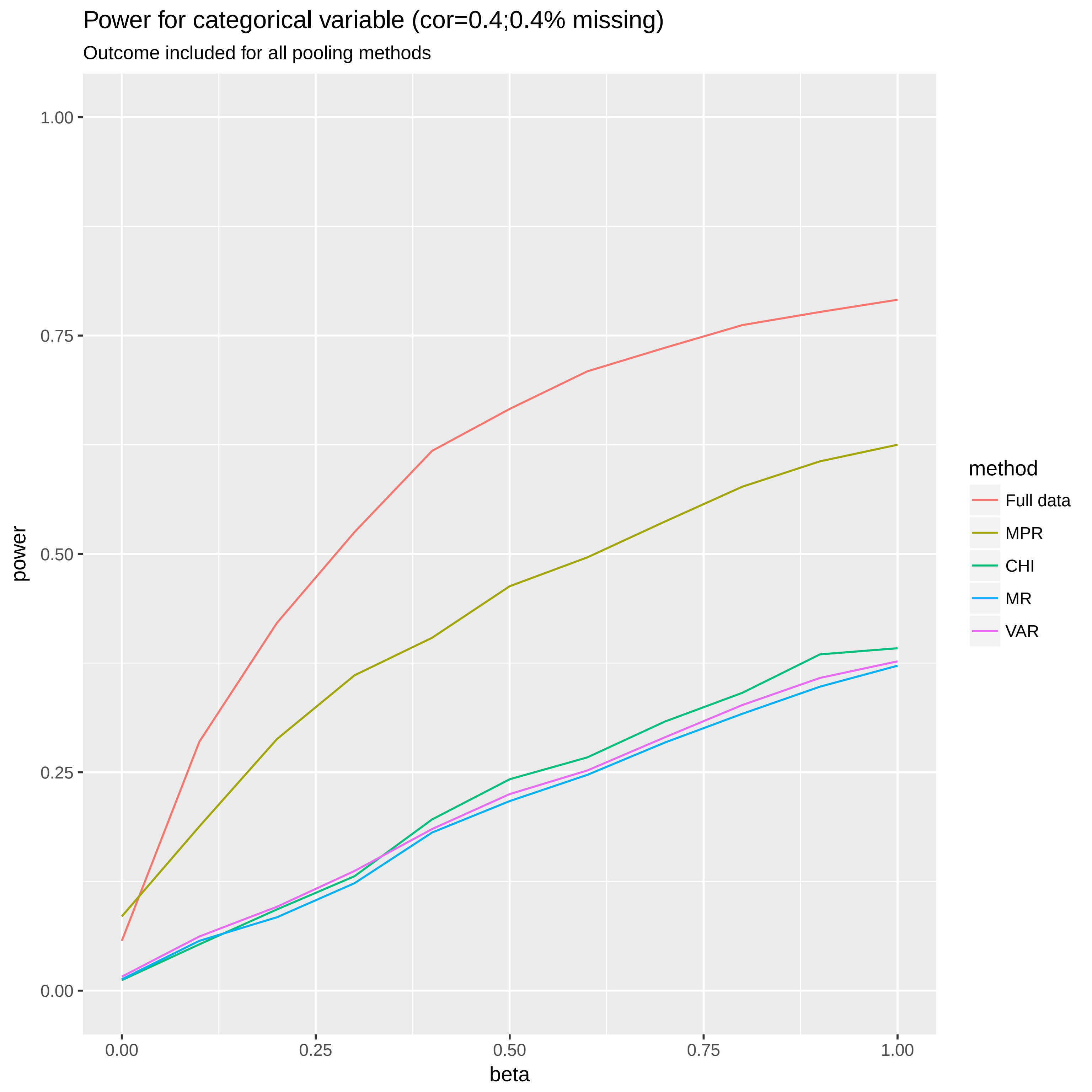
Power for categorical variable (cor=0.4;0.4% missing)
Outcome included for all pooling methods
power
1.00
0.75
0.50
0.25
0.00
beta
0.00
0.25
0.50
0.75
1.00
method
Full data
MPR
CHI
MR
VAR

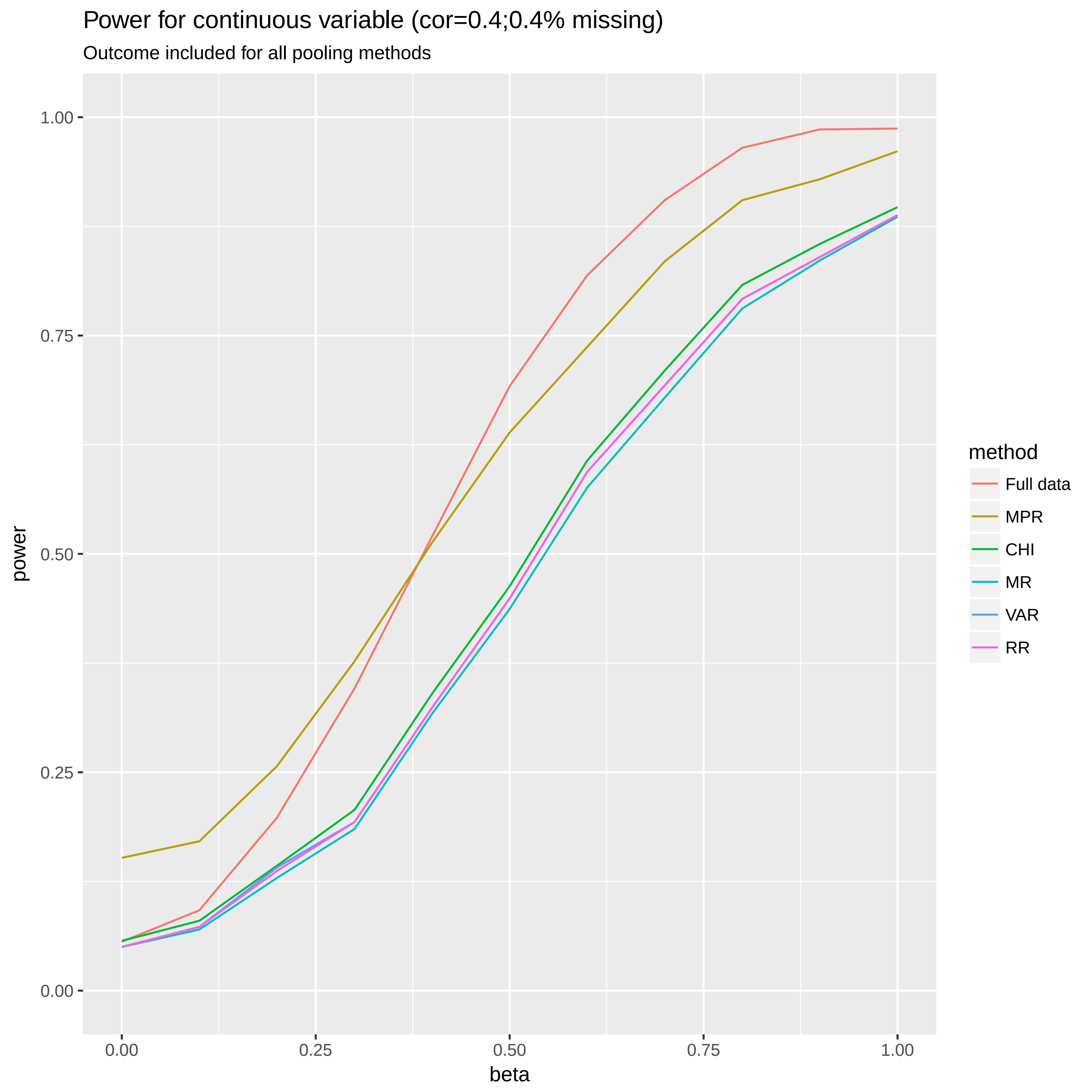

Power for continuous variable (cor=0.4;0.4% missing)
Outcome included for all pooling methods
power
1.00
0.75
0.50
0.25
0.00
beta
0.00
0.25
0.50
0.75
1.00
method
Full data
MPR
CHI
MR
VAR
RR

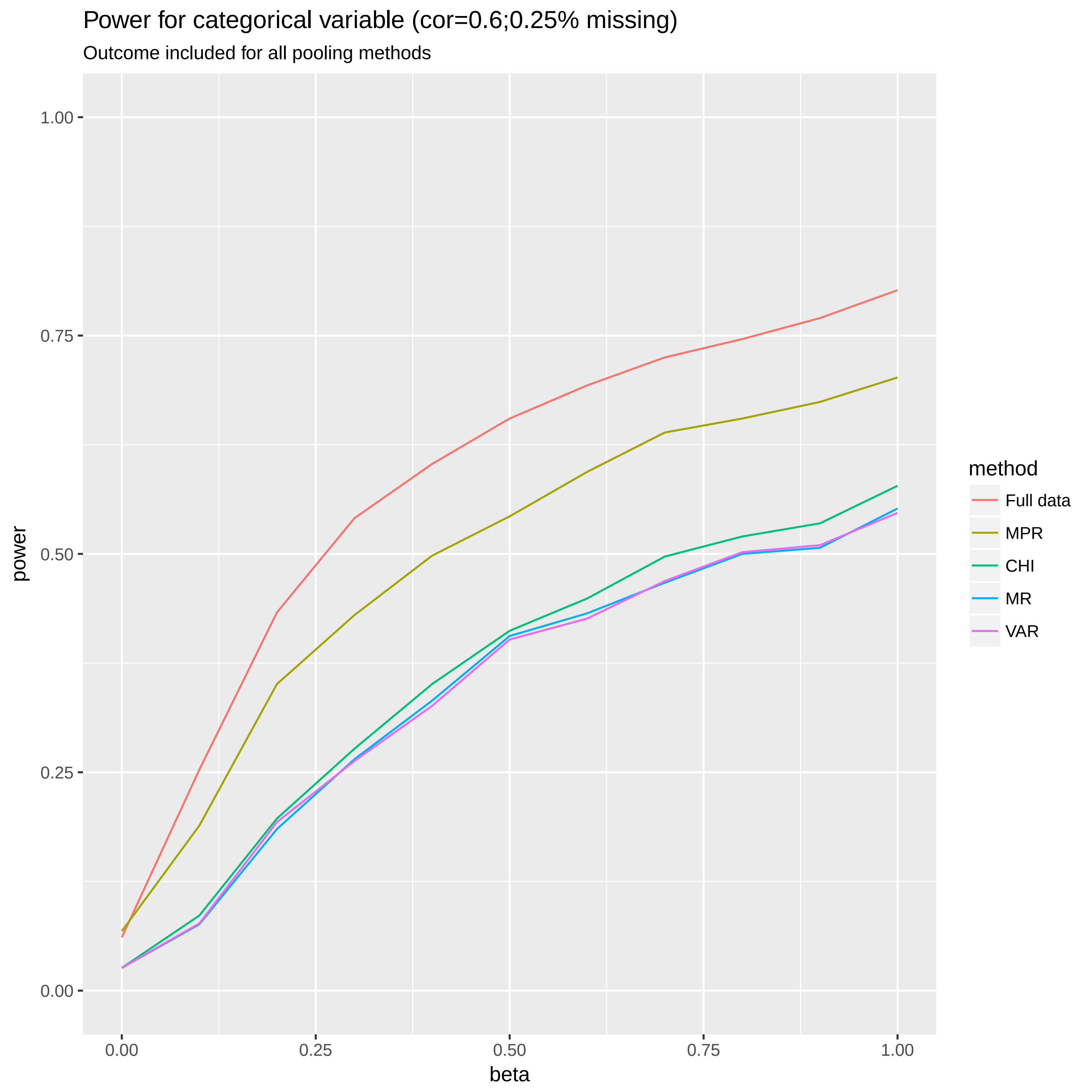
Power for categorical variable (cor=0.6;0.25% missing)
Outcome included for all pooling methods
power
beta
0.00
0.25
0.50
0.75
1.00
method
Full data
MPR
CHI
MR
VAR

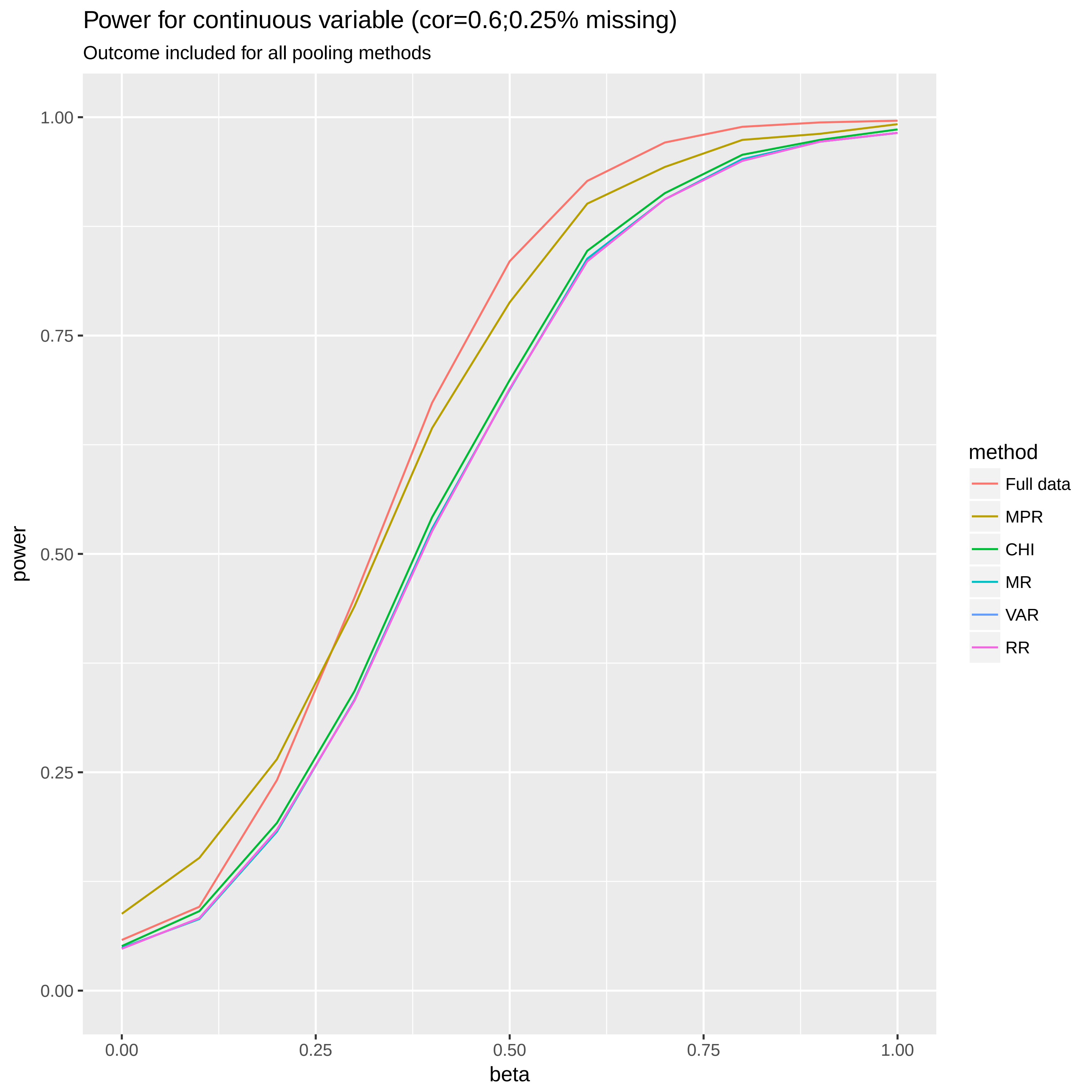
Power for continuous variable (cor=0.6;0.25% missing)
Outcome included for all pooling methods
power
1.00
0.75
0.50
0.25
0.00
beta
0.00
0.25
0.50
0.75
1.00
method
Full data
MPR
CHI
MR
VAR
RR

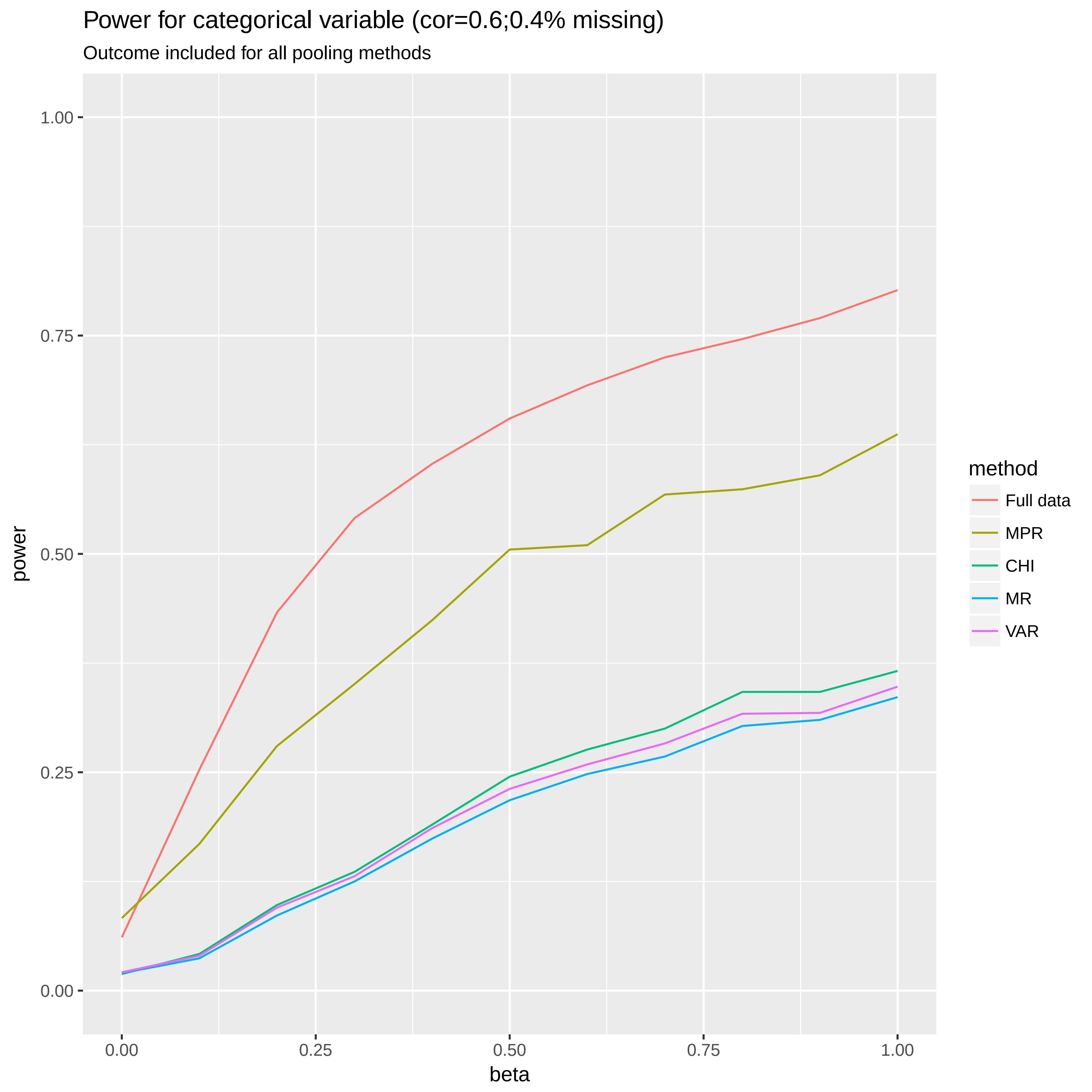

Power for categorical variable (cor=0.6;0.4% missing)
Outcome included for all pooling methods
power
1.00
0.75
0.50
0.25
0.00
beta
0.00
0.25
0.50
0.75
1.00
method
Full data
MPR
CHI
MR
VAR

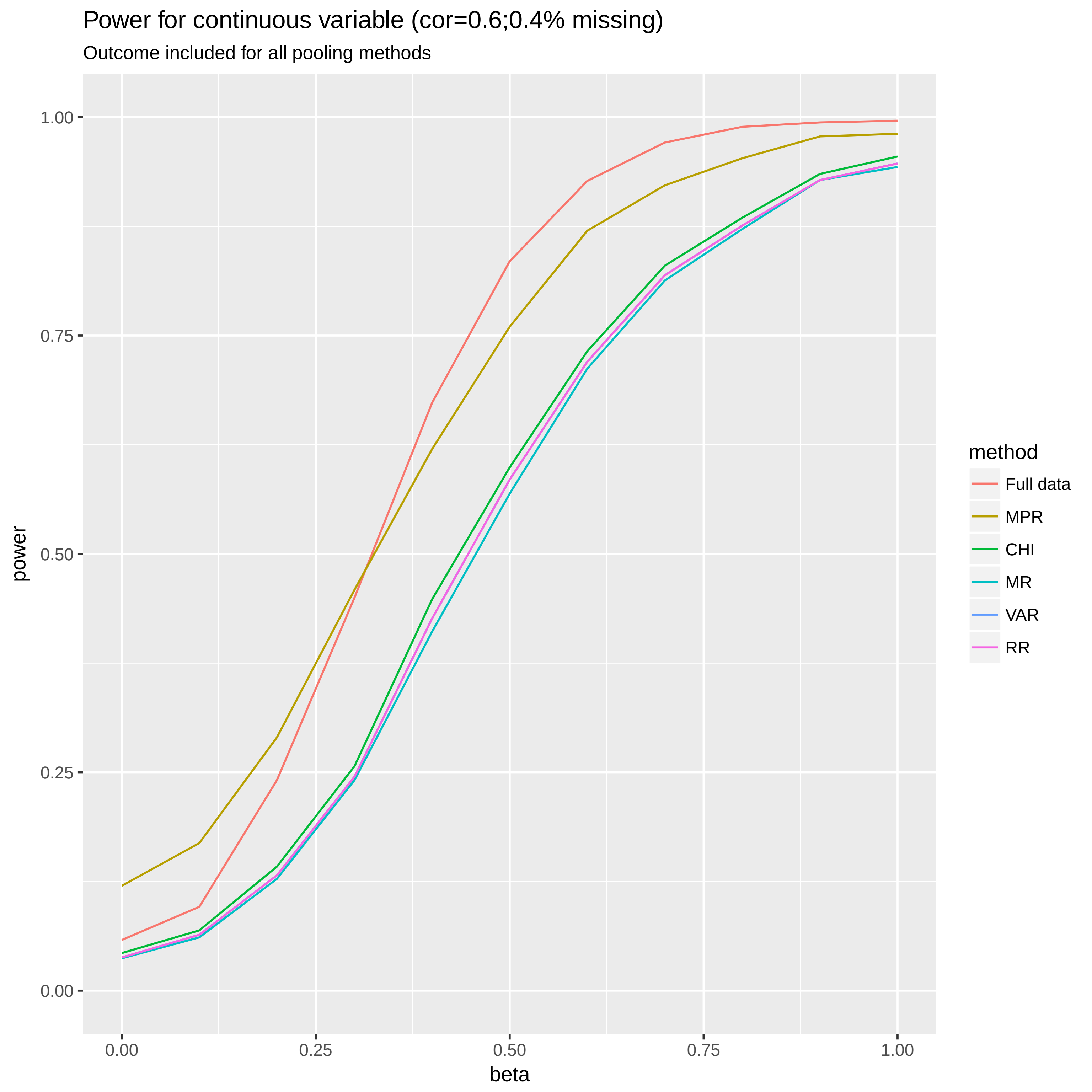
Power for continuous variable (cor=0.6;0.4% missing)
Outcome included for all pooling methods
power
beta
1.00
0.75
0.50
0.25
0.00
0.00
0.25
0.50
0.75
1.00
method
Full data
MPR
CHI
MR
VAR
RR

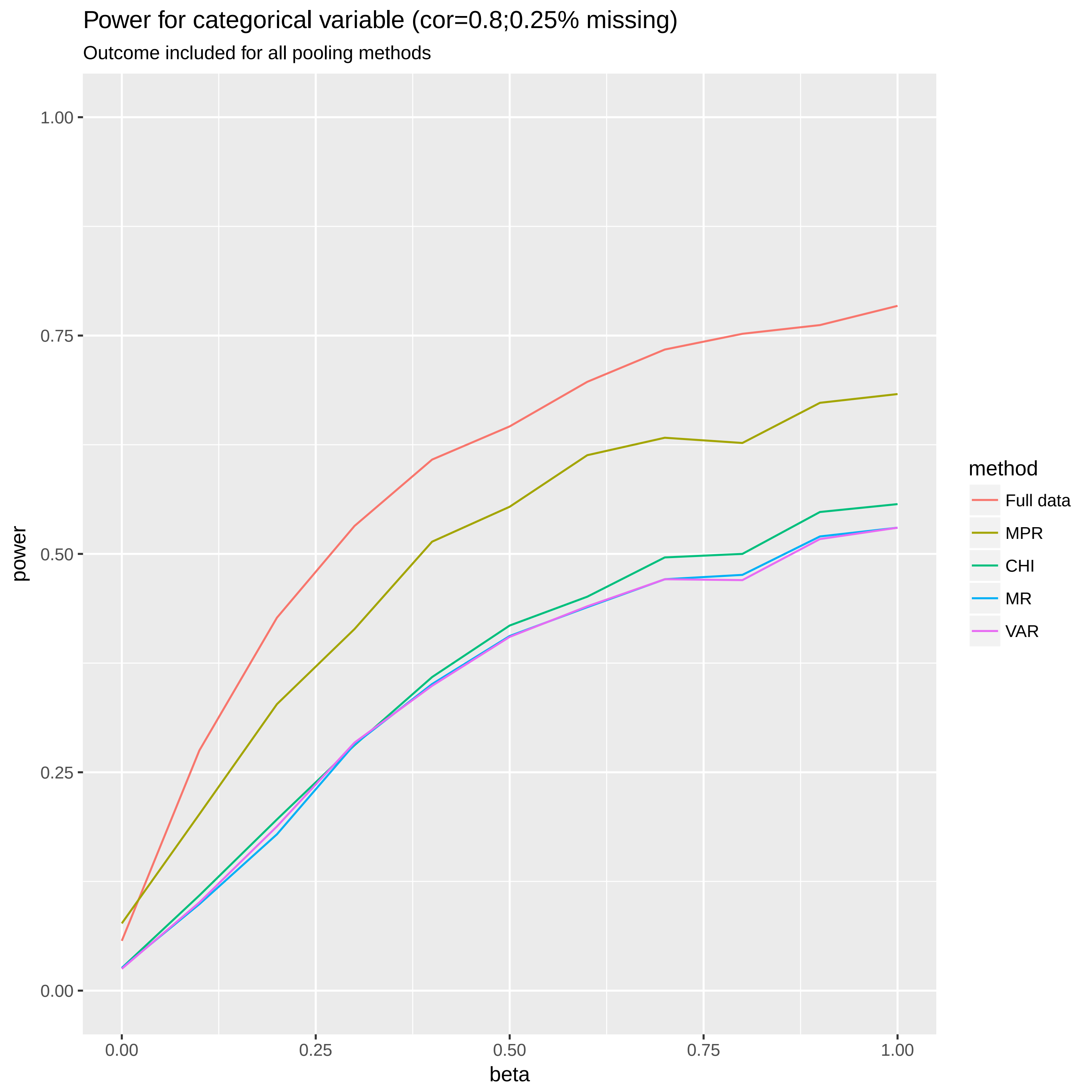

Power for categorical variable (cor=0.8;0.25% missing)
Outcome included for all pooling methods
power
beta
1.00
0.75
0.50
0.25
0.00
0.00
0.25
0.50
0.75
1.00
method
Full data
MPR
CHI
MR
VAR

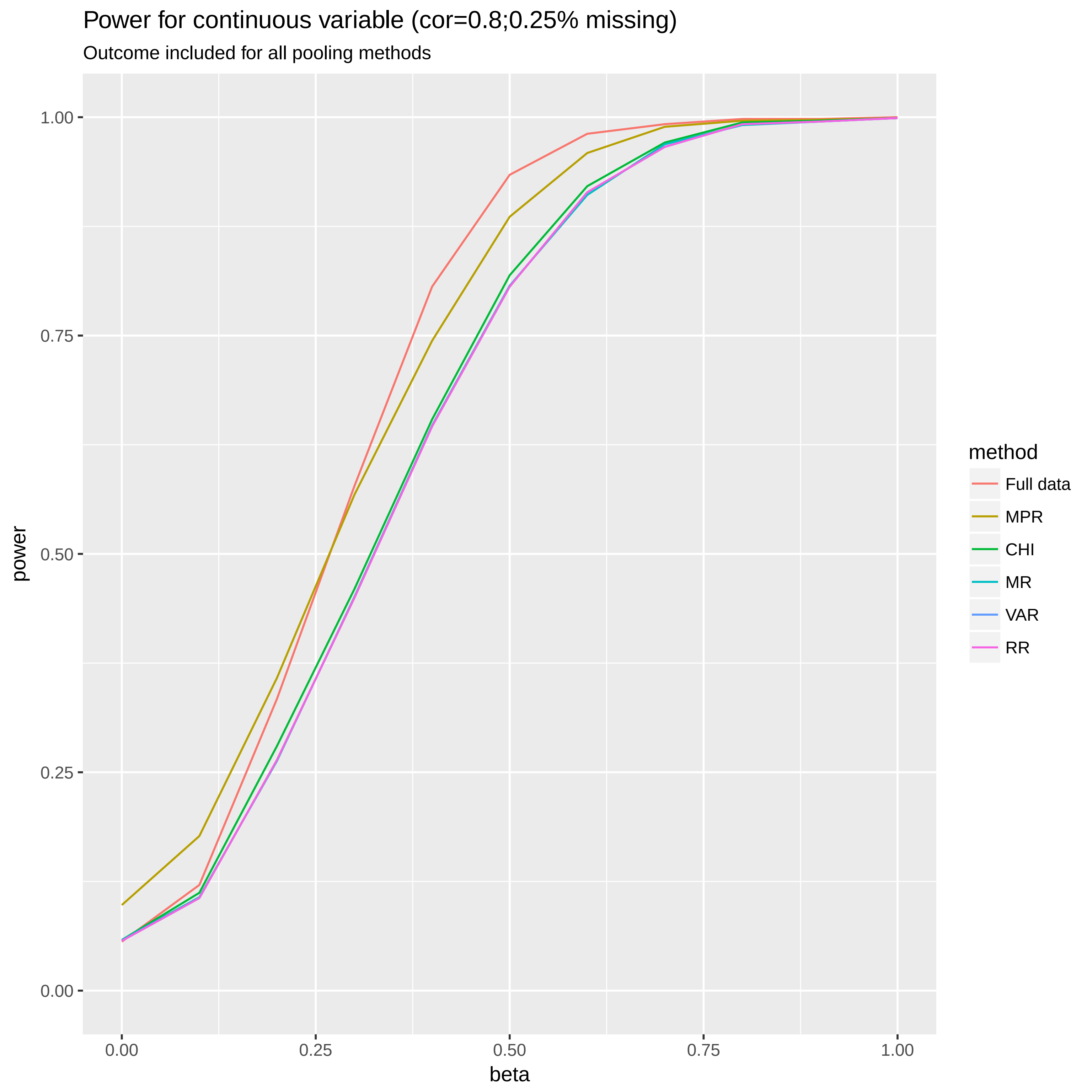
Power for continuous variable (cor=0.8;0.25% missing)
Outcome included for all pooling methods
power
beta
1.00
0.75
0.50
0.25
0.00
0.00
0.25
0.50
0.75
1.00
method
Full data
MPR
CHI
MR
VAR
RR

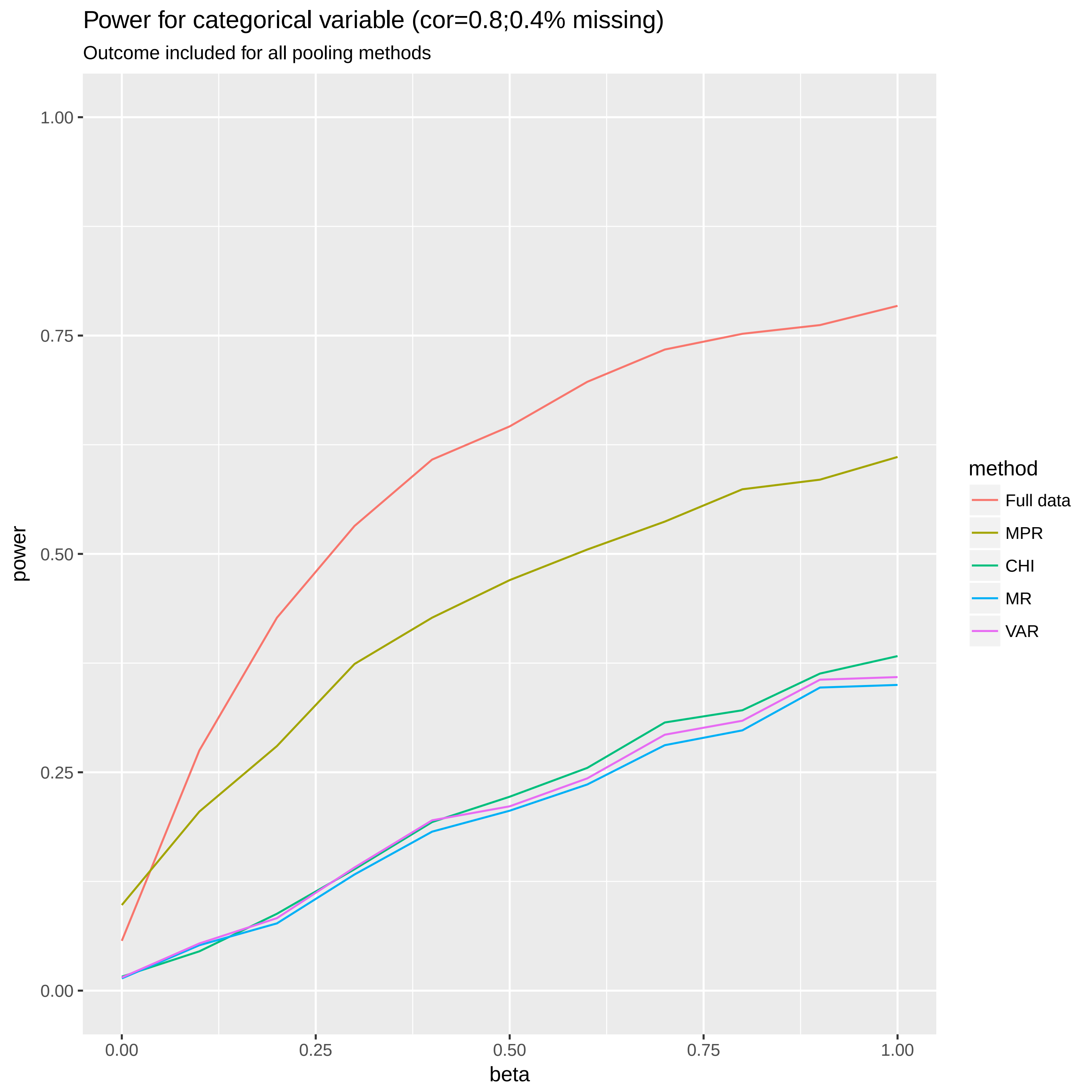

Power for categorical variable (cor=0.8;0.4% missing)
Outcome included for all pooling methods
power
1.00
0.75
0.50
0.25
0.00
beta
0.00
0.25
0.50
0.75
1.00
method
Full data
MPR
CHI
MR
VAR

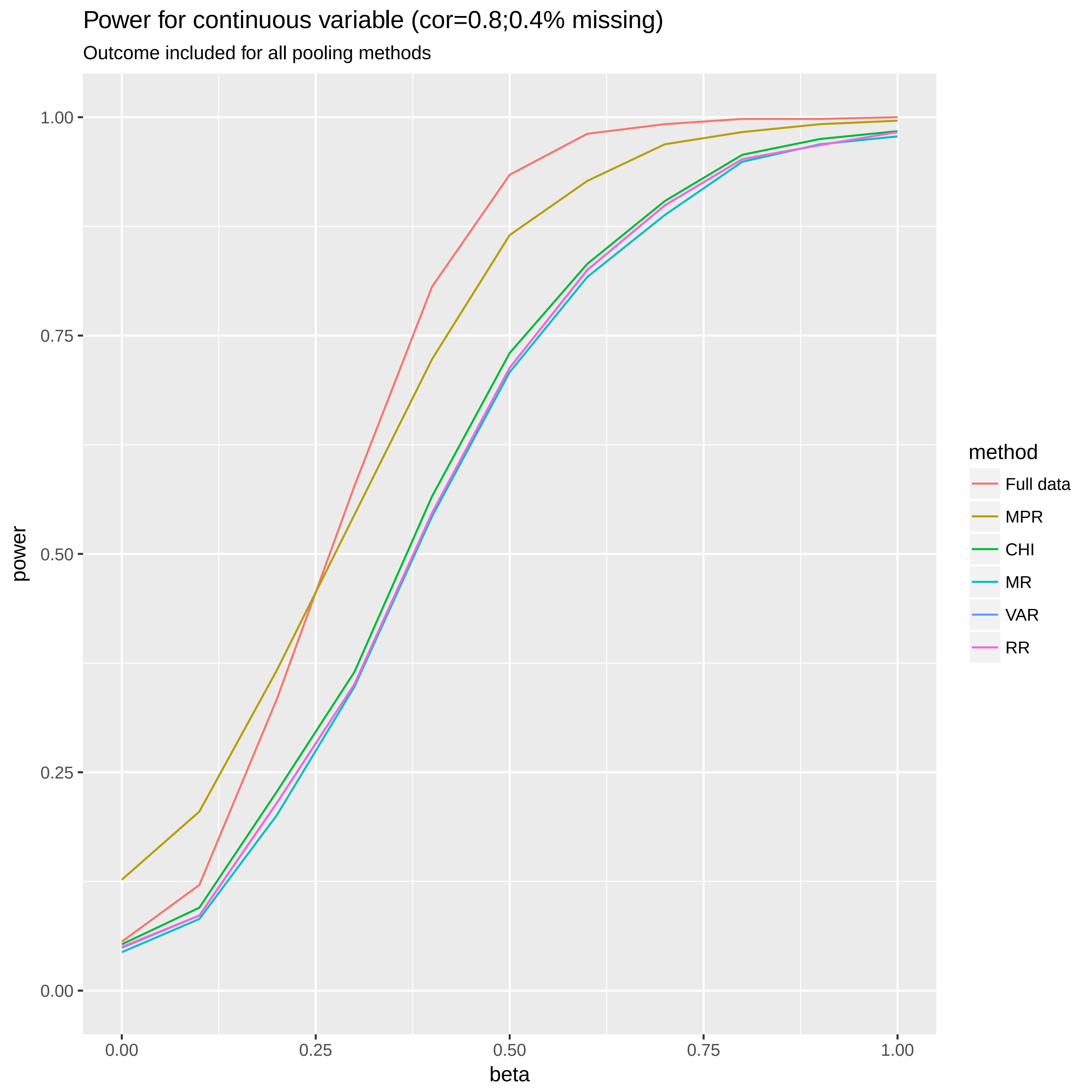

Power for continuous variable (cor=0.8;0.4% missing)
Outcome included for all pooling methods
power
beta
1.00
0.75
0.50
0.25
0.00
0.00
0.25
0.50
0.75
1.00
method
Full data
MPR
CHI
MR
VAR
RR

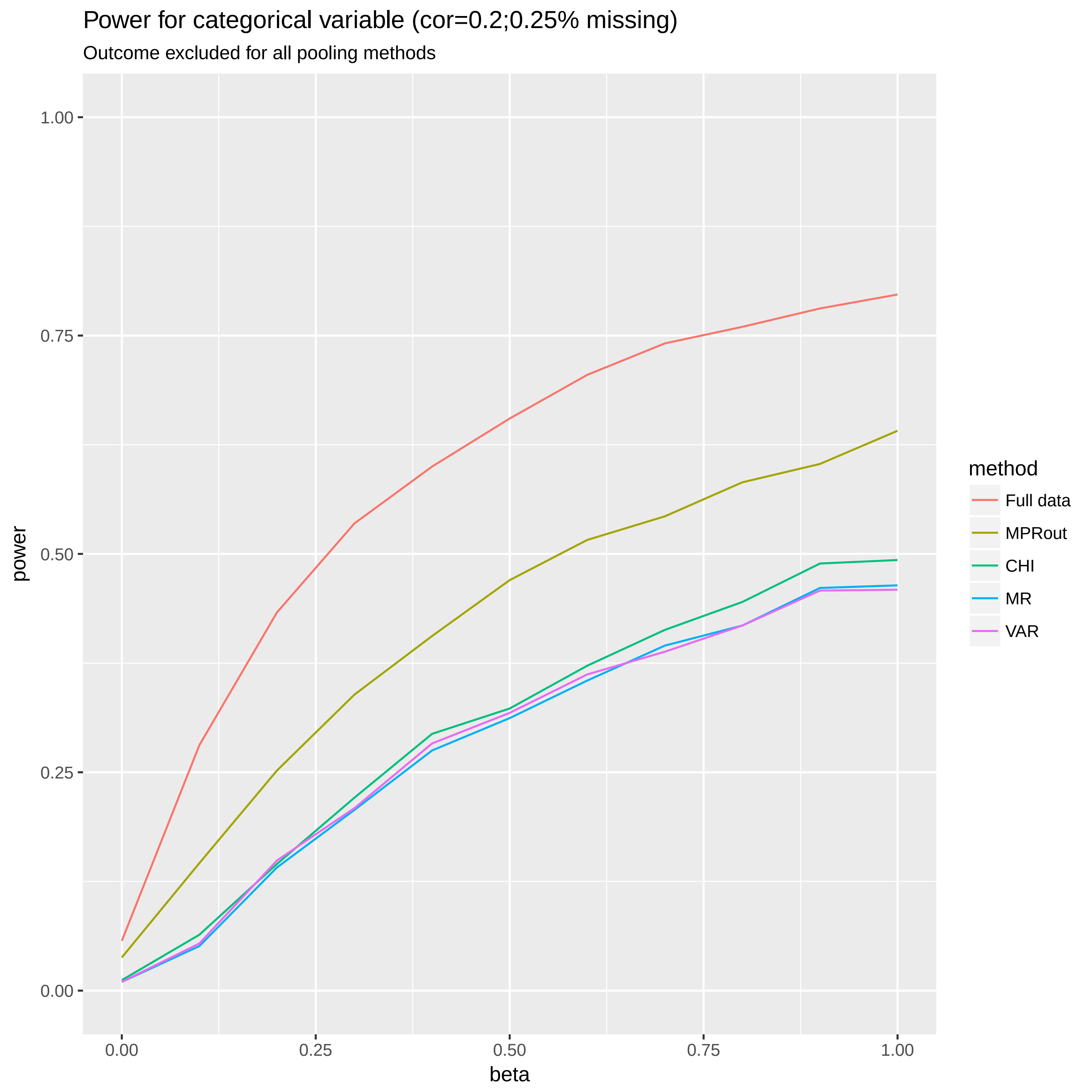

Power for categorical variable (cor=0.2;0.25% missing)
Outcome excluded for all pooling methods
power
beta
1.00
0.75
0.50
0.25
0.00
0.00
0.25
0.50
0.75
1.00
method
Full data
MPRout
CHI
MR
VAR

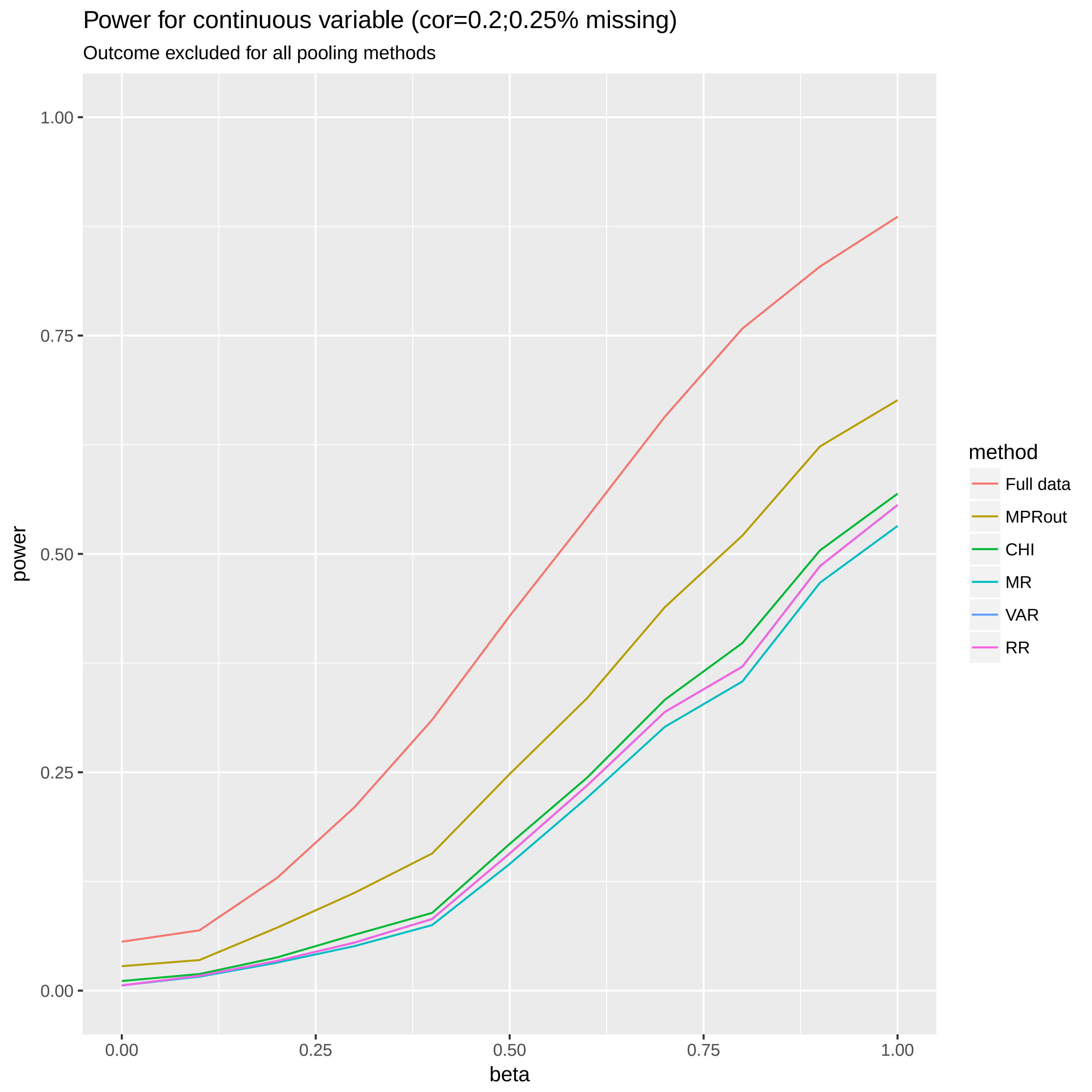

Power for continuous variable (cor=0.2;0.25% missing)
Outcome excluded for all pooling methods
power
beta
1.00
0.75
0.50
0.25
0.00
0.00
0.25
0.50
0.75
1.00
method
Full data
MPRout
CHI
MR
VAR
RR

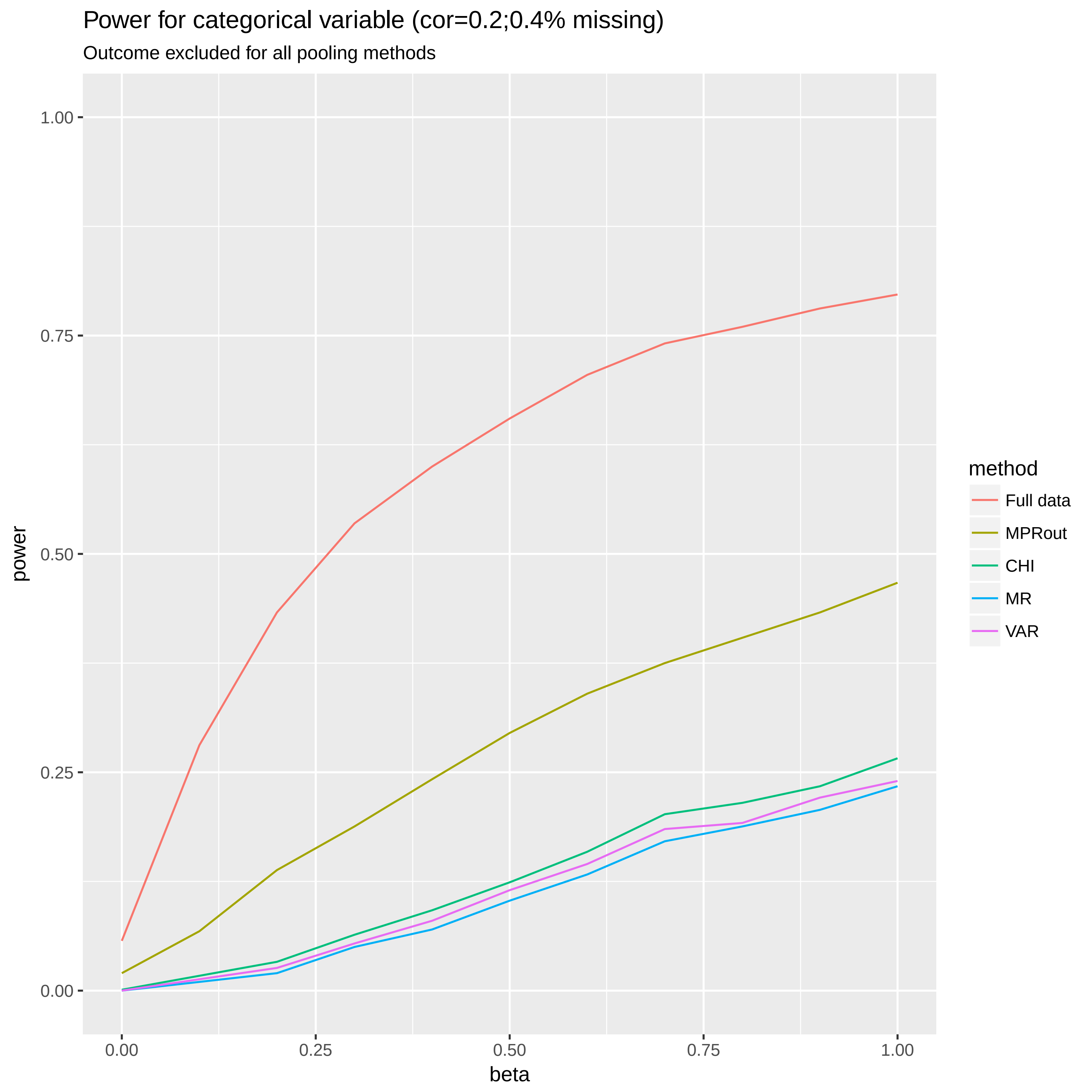
Power for categorical variable (cor=0.2;0.4% missing)
Outcome excluded for all pooling methods
power
1.00
0.75
0.50
0.25
0.00
0.00
0.25
0.50
0.75
1.00
beta
method
Full data
MPRout
CHI
MR
VAR

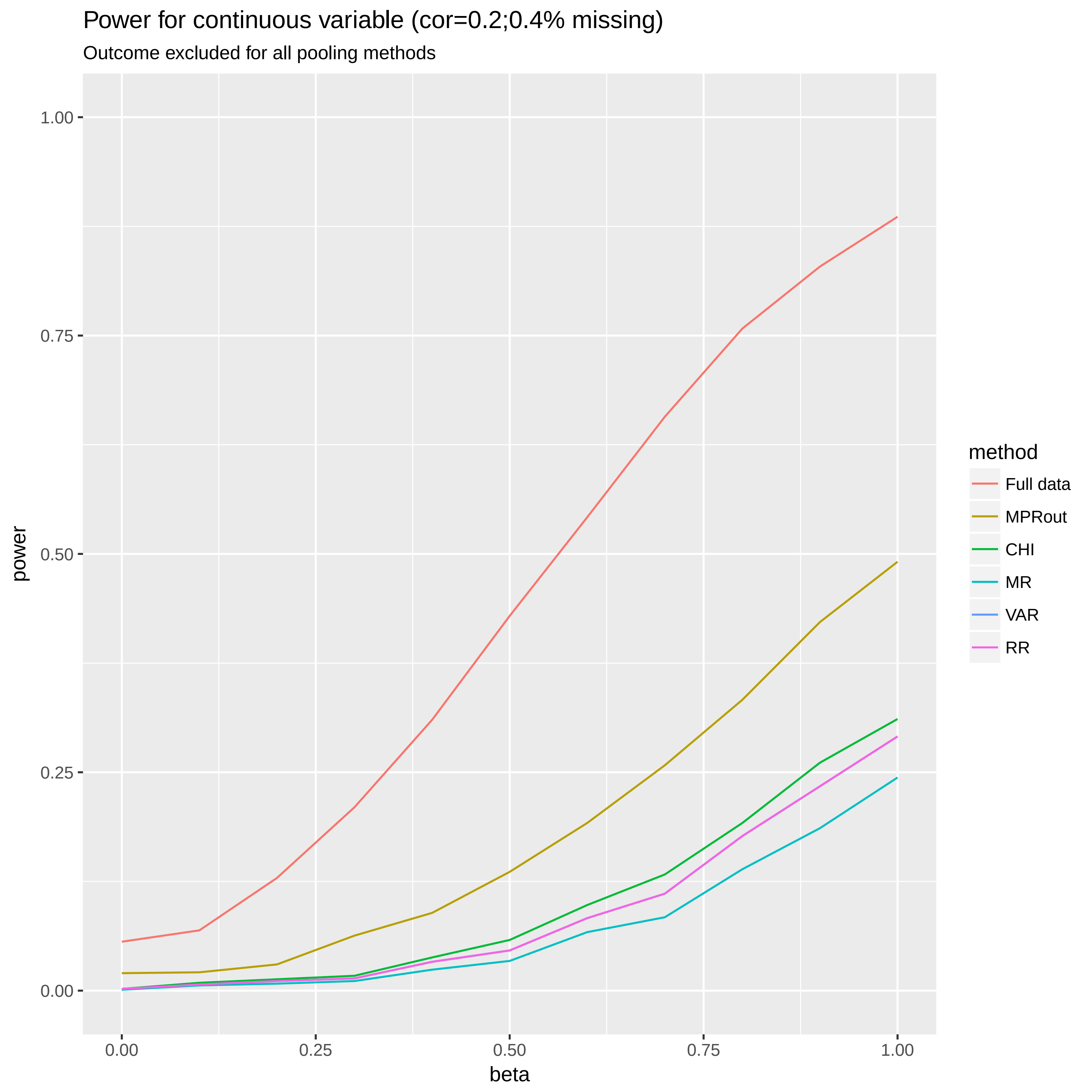
Power for continuous variable (cor=0.2;0.4% missing)
Outcome excluded for all pooling methods
power
1.00
0.75
0.50
0.25
0.00
0.00
0.25
0.50
0.75
1.00
beta
method
Full data
MPRout
CHI
MR
VAR
RR

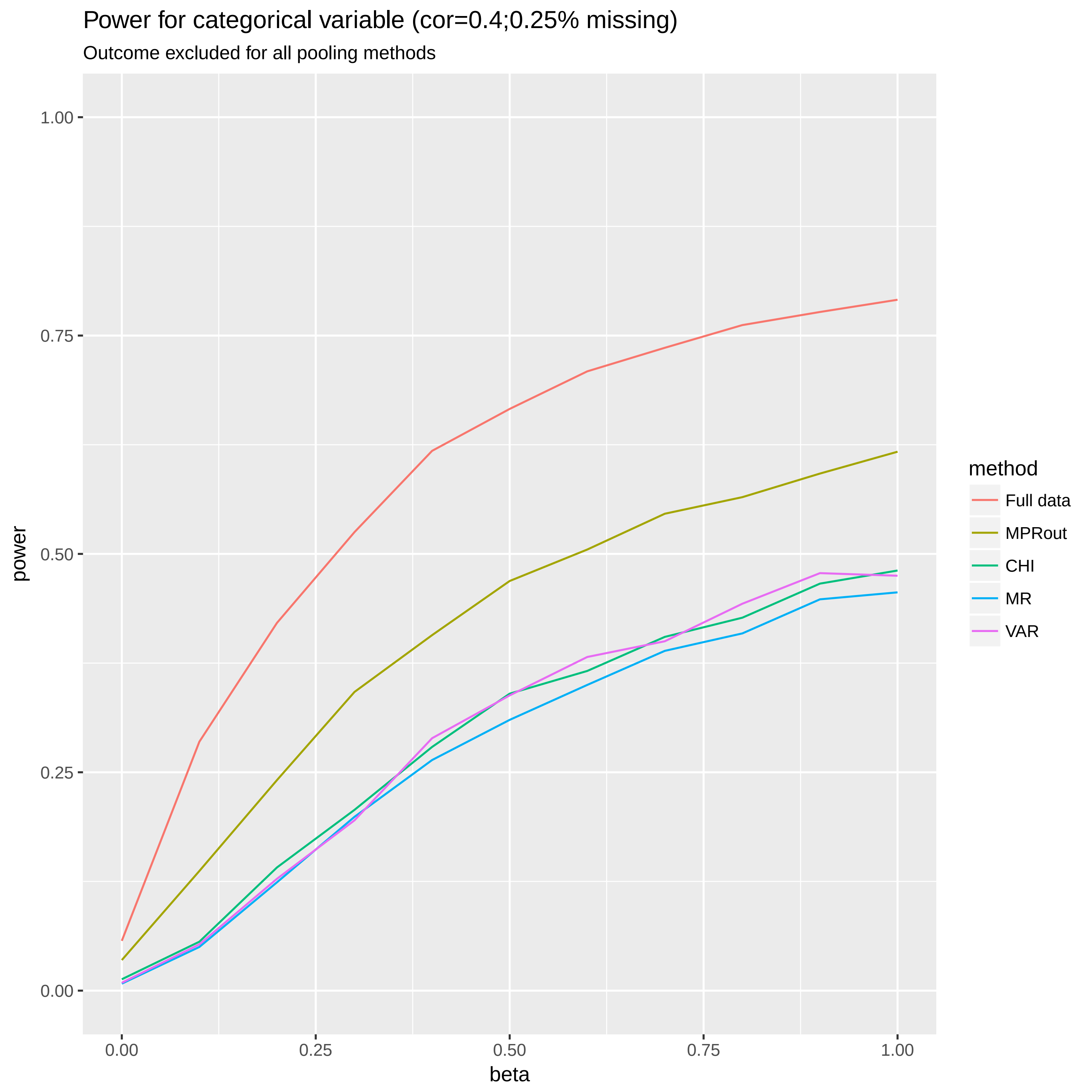

Power for categorical variable (cor=0.4;0.25% missing)
Outcome excluded for all pooling methods
power
beta
0.00
0.25
0.50
0.75
1.00
method
Full data
MPRout
CHI
MR
VAR

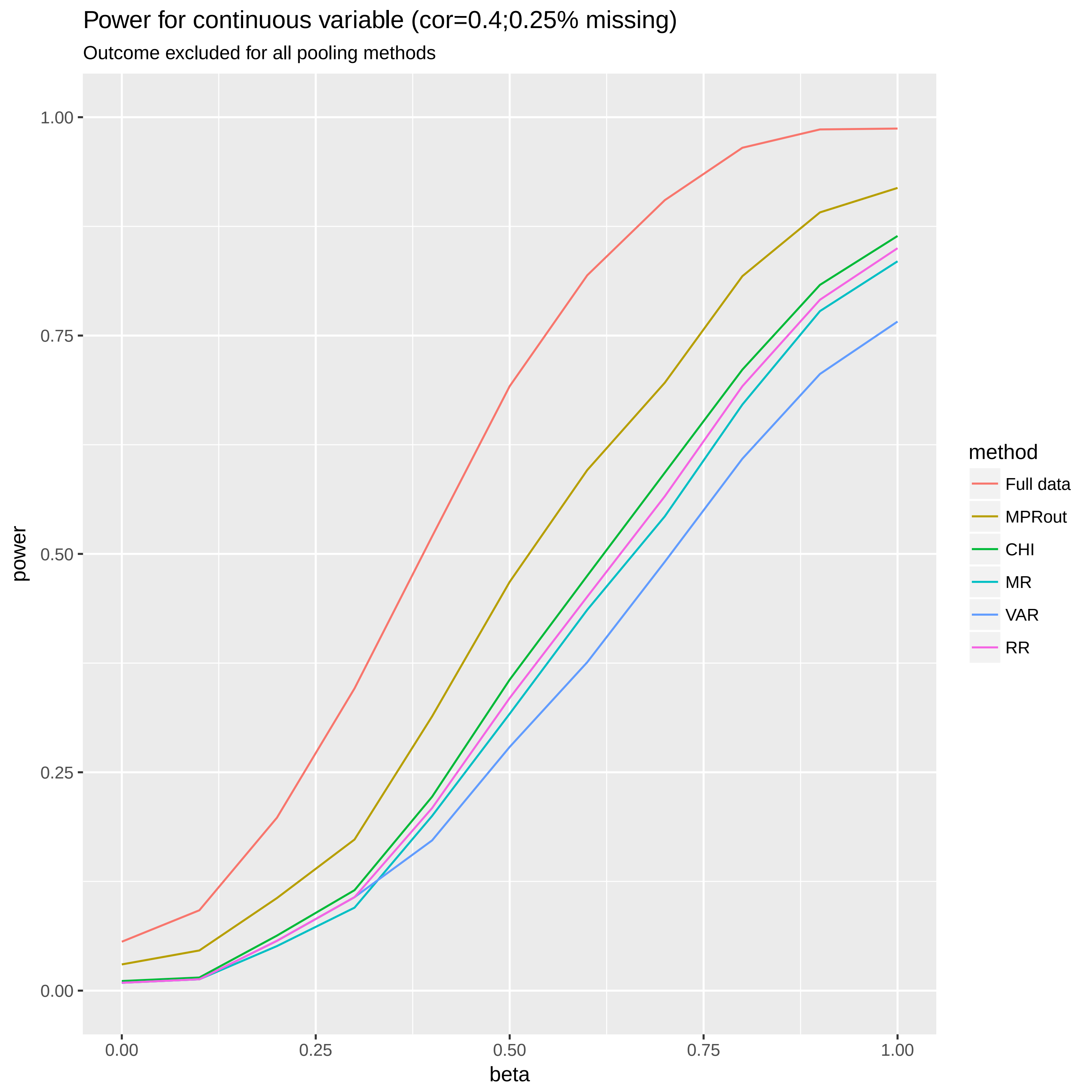
Power for continuous variable (cor=0.4;0.25% missing)
Outcome excluded for all pooling methods
power
beta
1.00
0.75
0.50
0.25
0.00
0.00
0.25
0.50
0.75
1.00
method
Full data
MPRout
CHI
MR
VAR
RR

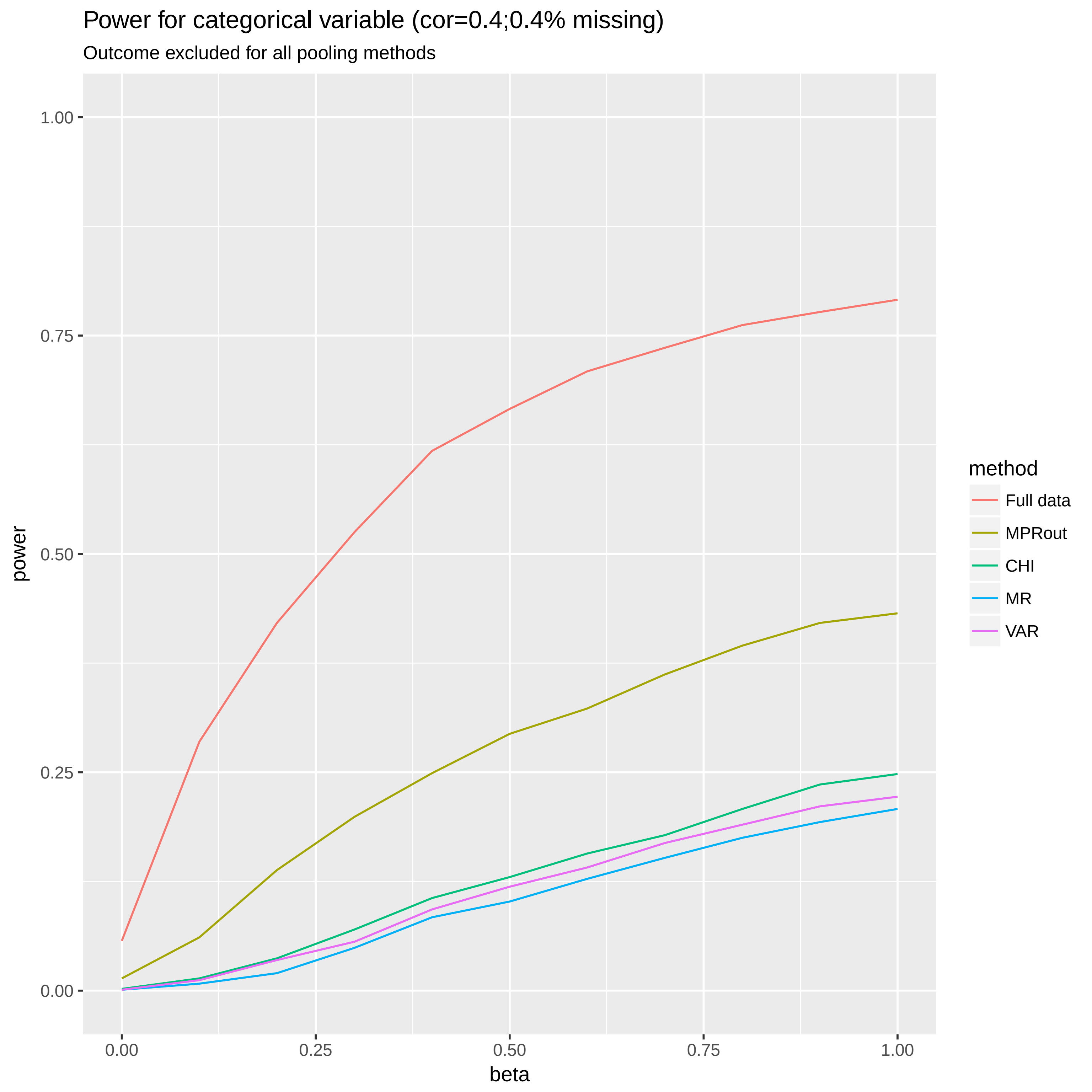
Power for categorical variable (cor=0.4;0.4% missing)
Outcome excluded for all pooling methods
power
beta
0.00
0.25
0.50
0.75
1.00
method
Full data
MPRout
CHI
MR
VAR

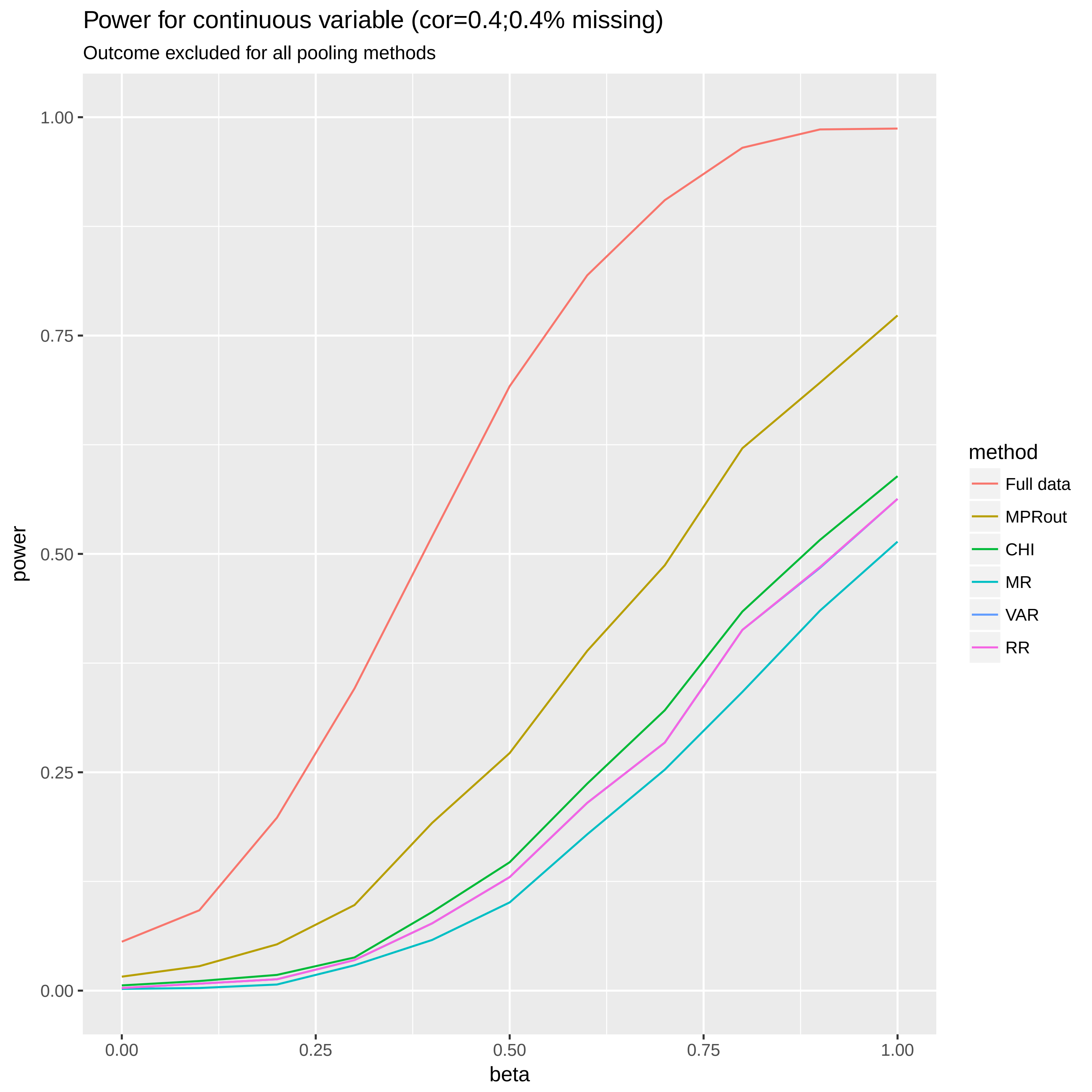
Power for continuous variable (cor=0.4;0.4% missing)
Outcome excluded for all pooling methods
power
beta
1.00
0.75
0.50
0.25
0.00
0.00
0.25
0.50
0.75
1.00
method
Full data
MPRout
CHI
MR
VAR
RR

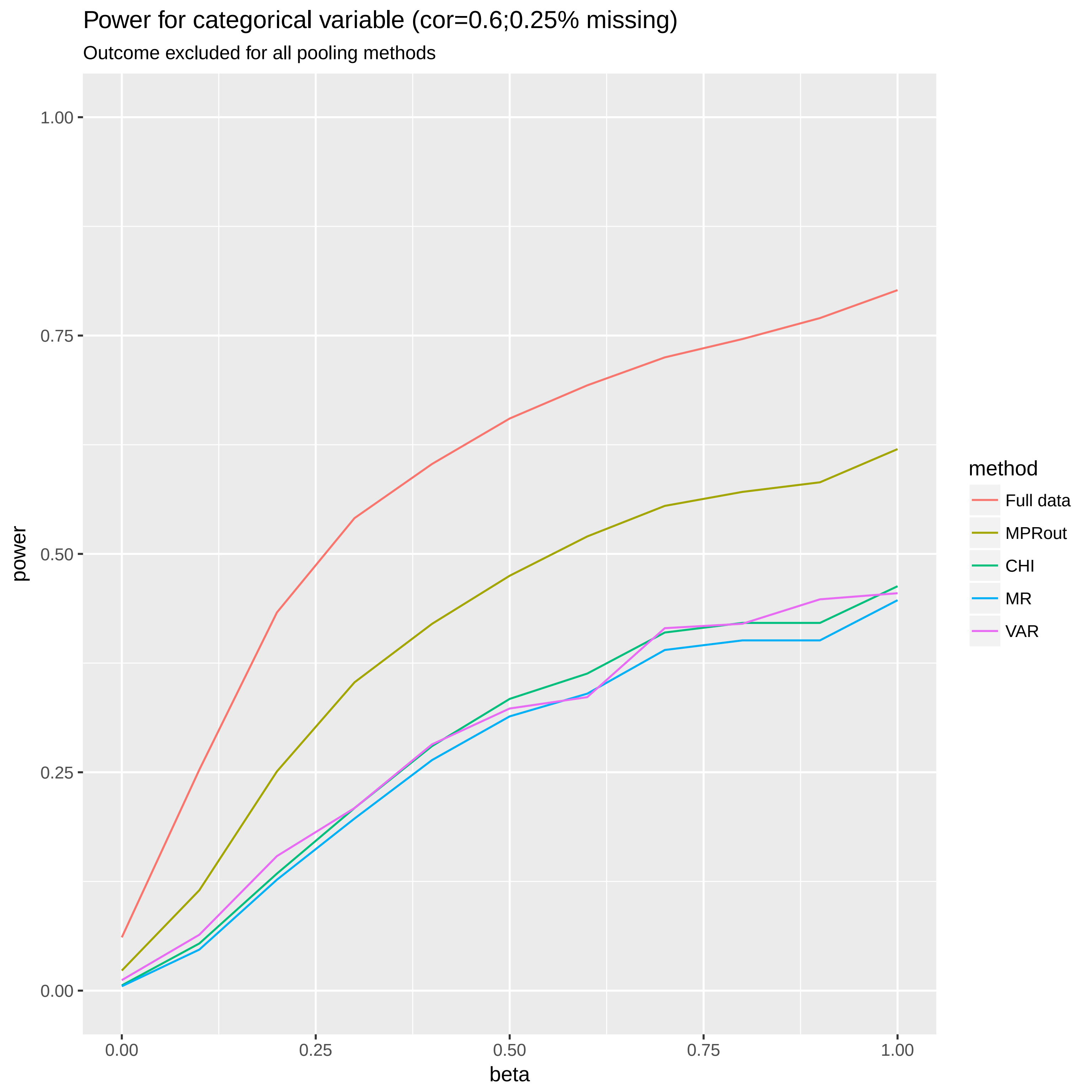
Power for categorical variable (cor=0.6;0.25% missing)
Outcome excluded for all pooling methods
power
1.00
0.75
0.50
0.25
0.00
beta
0.00
0.25
0.50
0.75
1.00
method
Full data
MPRout
CHI
MR
VAR

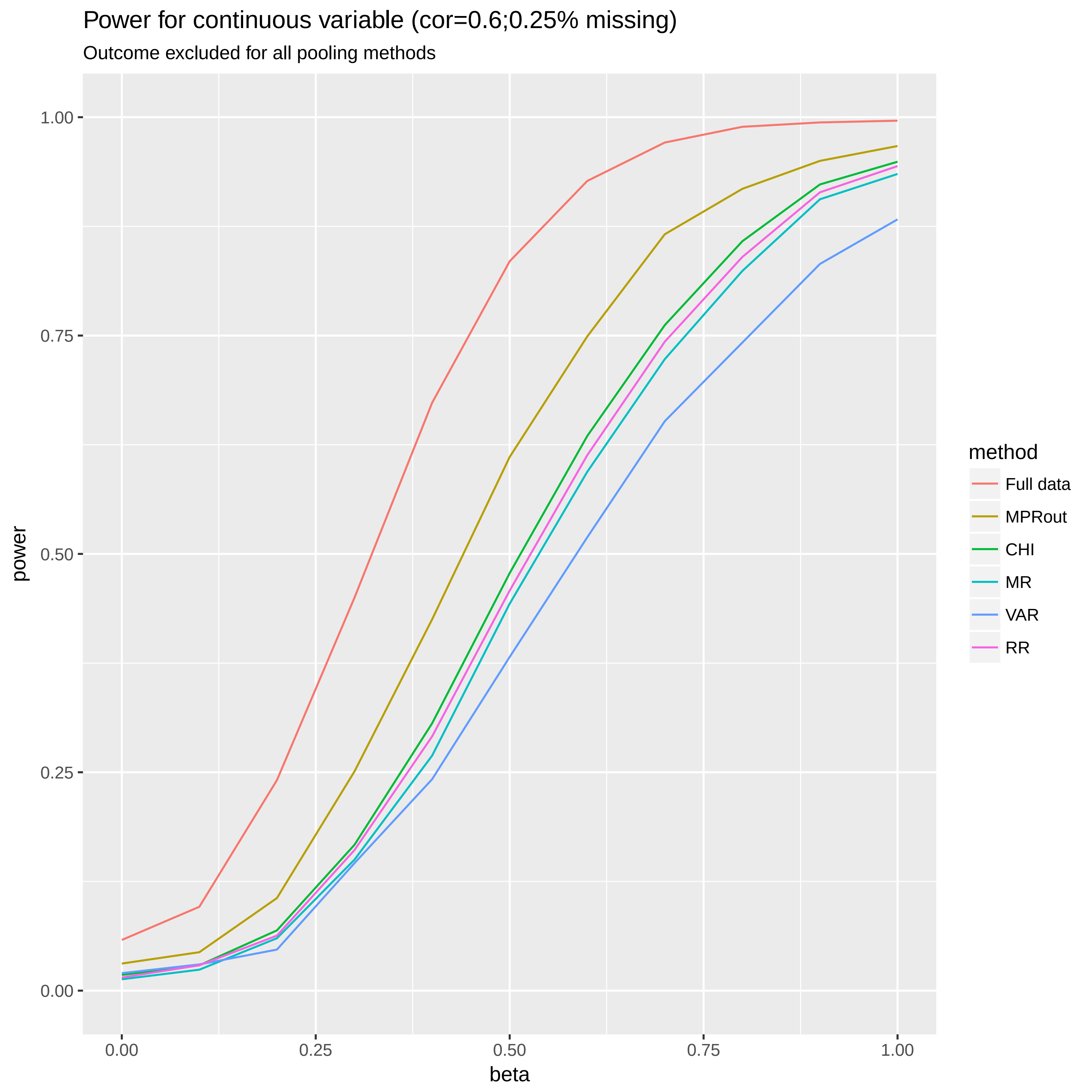

Power for continuous variable (cor=0.6;0.25% missing)
Outcome excluded for all pooling methods
power
beta
1.00
0.75
0.50
0.25
0.00
0.00
0.25
0.50
0.75
1.00
method
Full data
MPRout
CHI
MR
VAR
RR

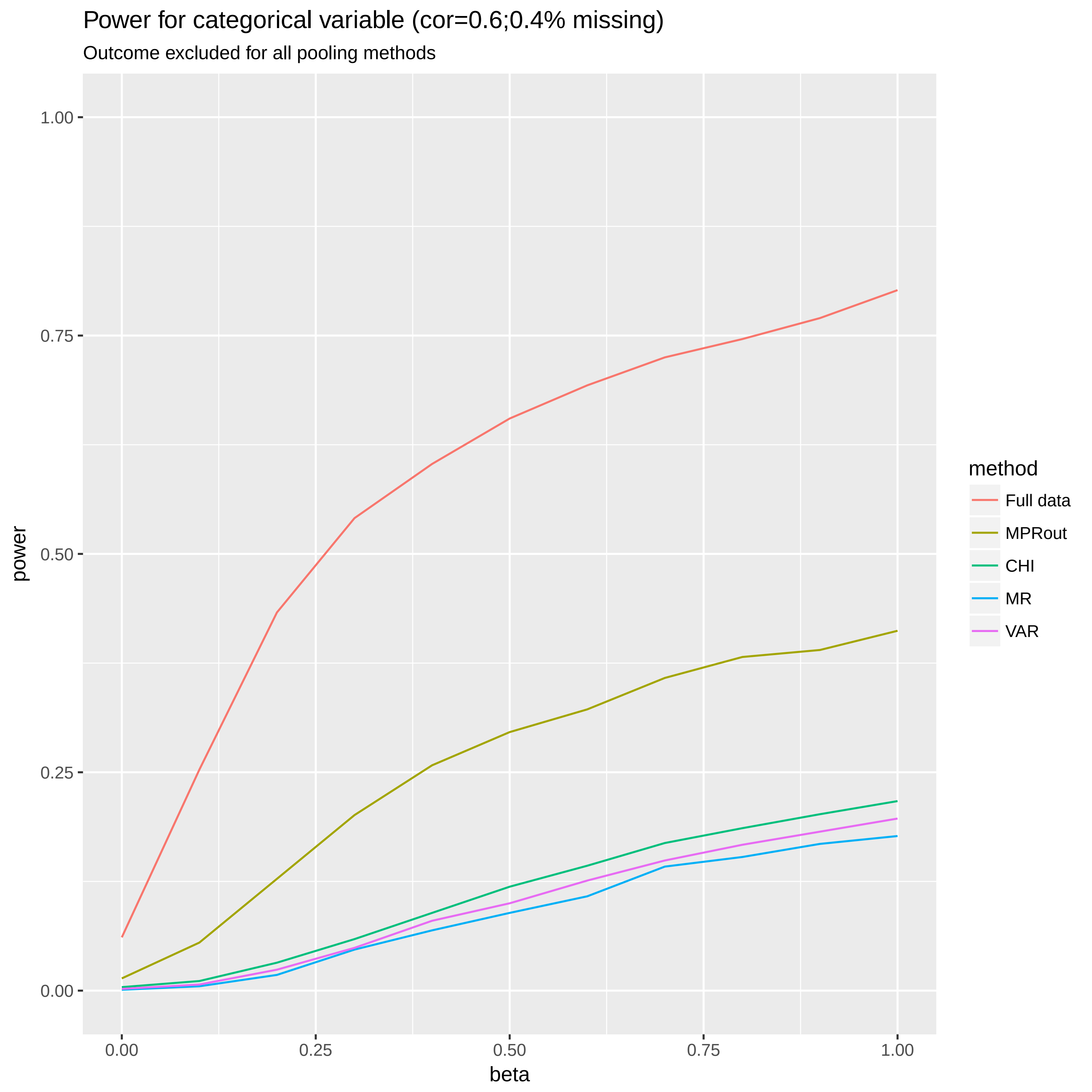

Power for categorical variable (cor=0.6;0.4% missing)
Outcome excluded for all pooling methods
power
1.00
0.75
0.50
0.25
0.00
0.00
0.25
0.50
0.75
1.00
beta
method
Full data
MPRout
CHI
MR
VAR

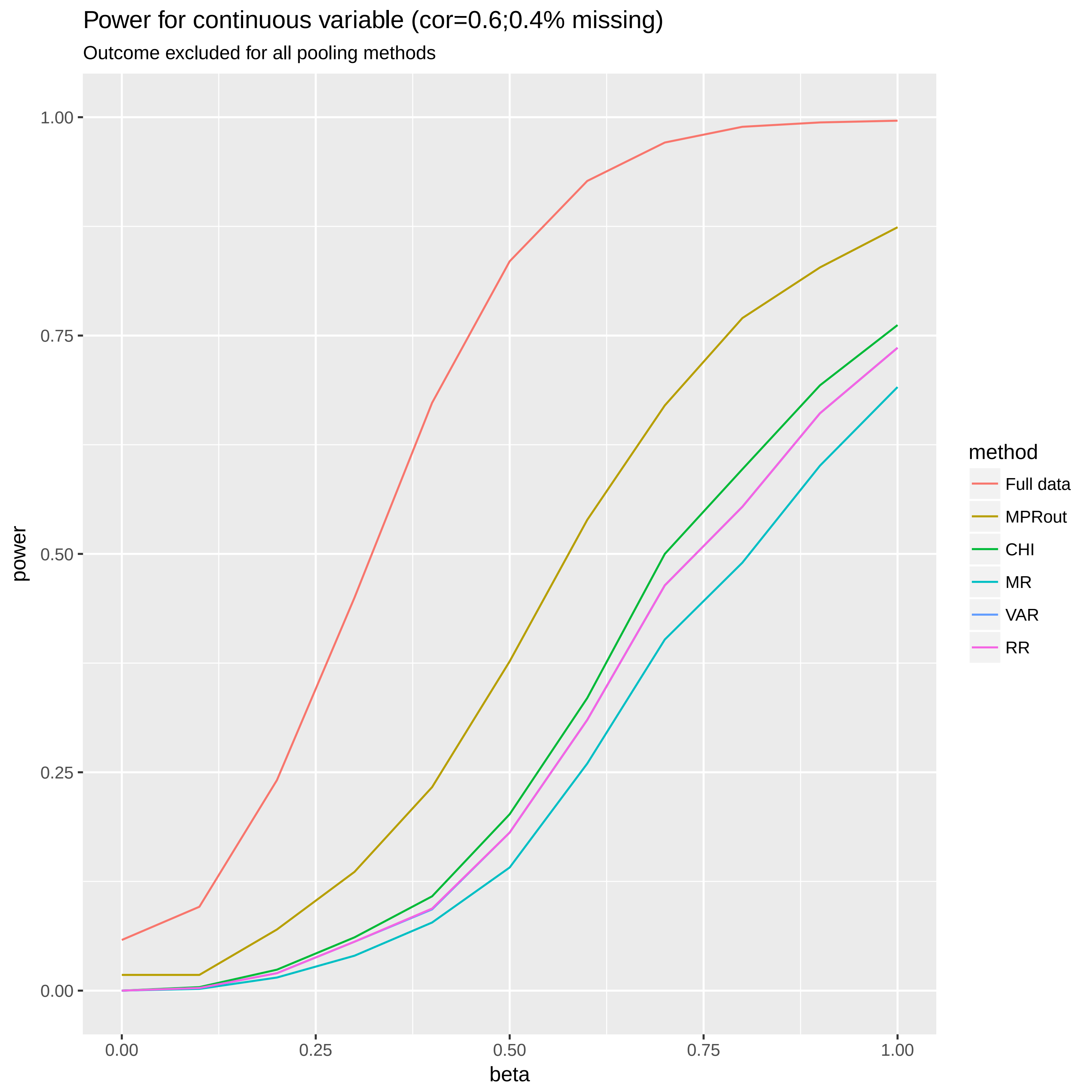

Power for continuous variable (cor=0.6;0.4% missing)
Outcome excluded for all pooling methods
power
beta
1.00
0.75
0.50
0.25
0.00
0.00
0.25
0.50
0.75
1.00
method
Full data
MPRout
CHI
MR
VAR
RR

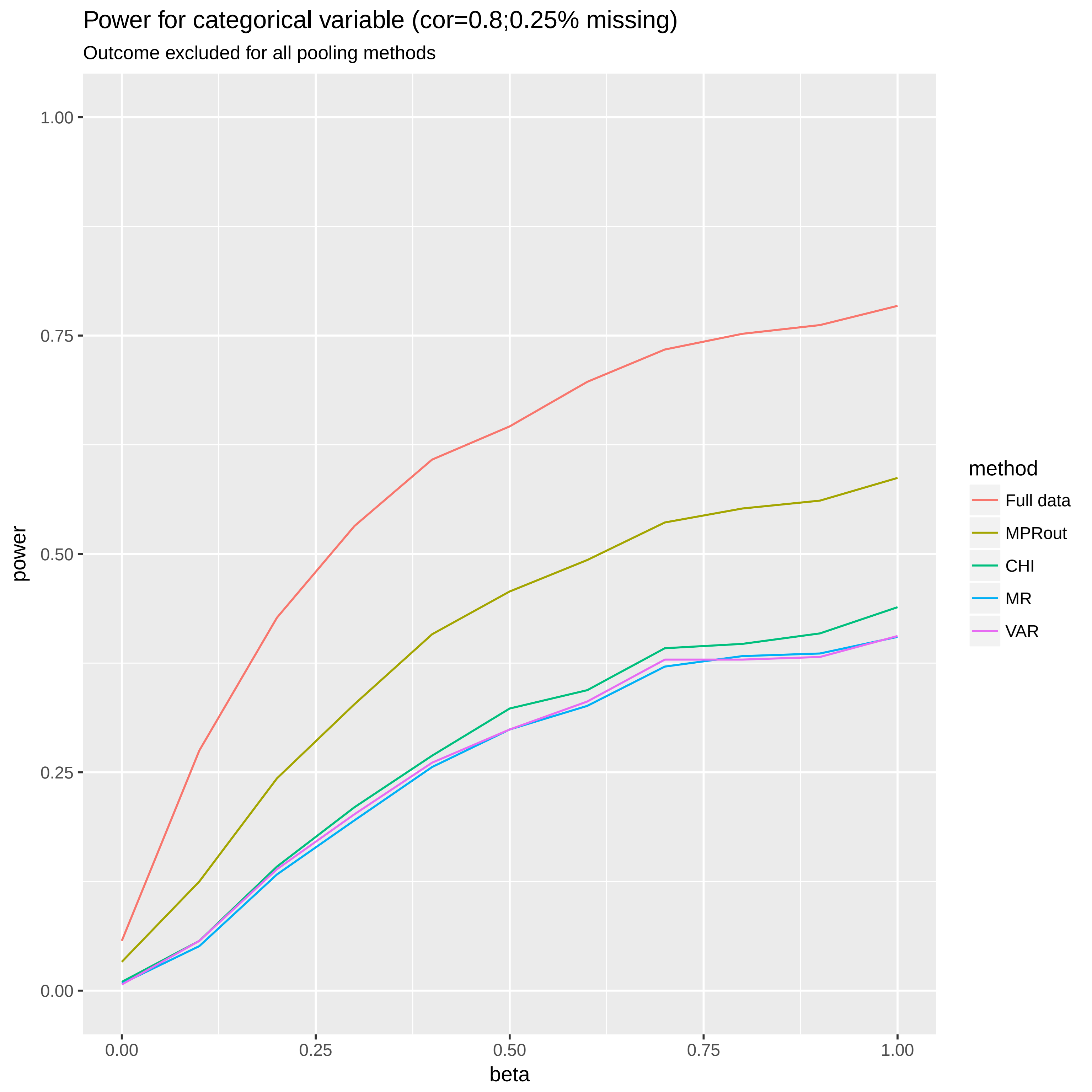

Power for categorical variable (cor=0.8;0.25% missing)
Outcome excluded for all pooling methods
power
beta
1.00
0.75
0.50
0.25
0.00
0.00
0.25
0.50
0.75
1.00
method
Full data
MPRout
CHI
MR
VAR

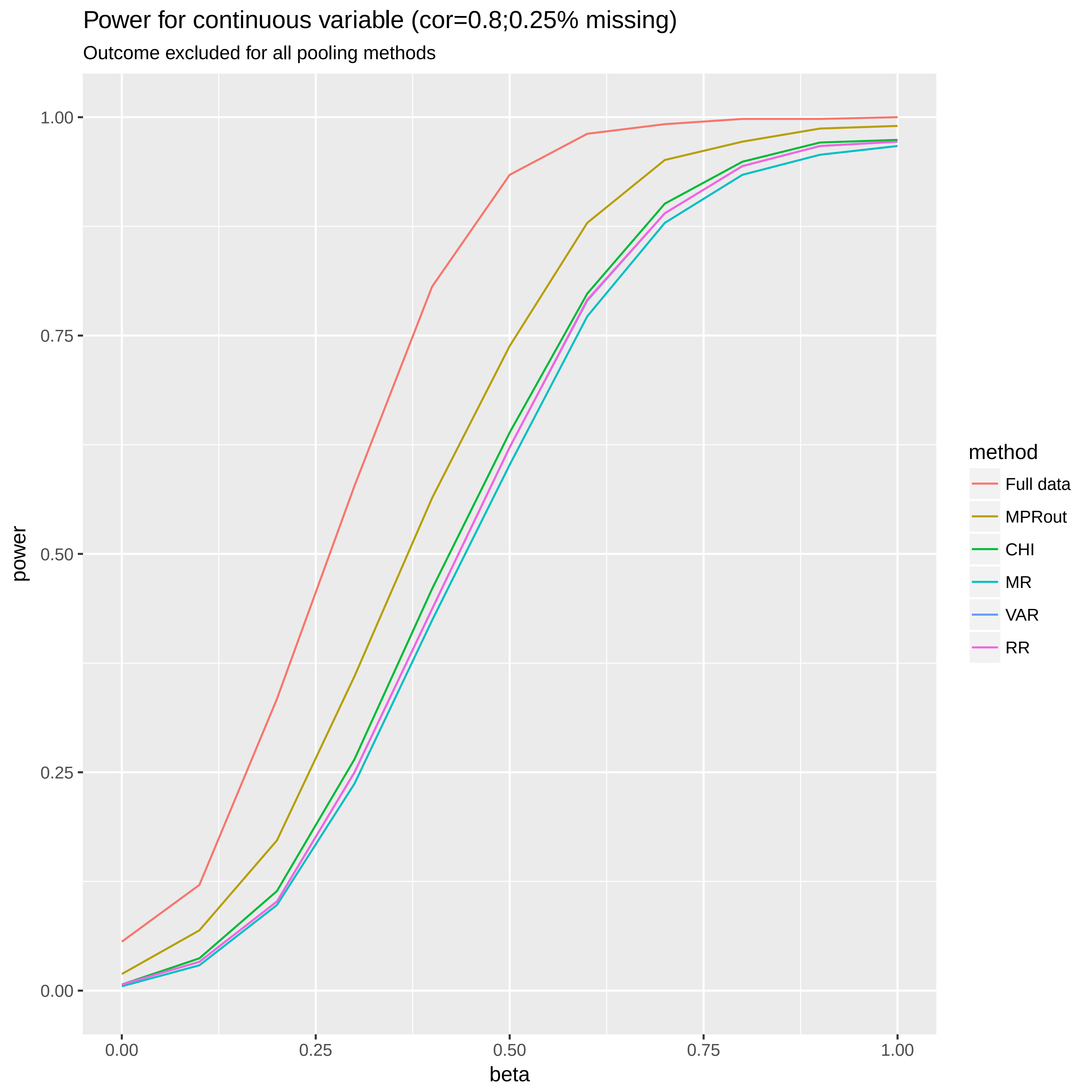
Power for continuous variable (cor=0.8;0.25% missing)
Outcome excluded for all pooling methods
power
beta
1.00
0.75
0.50
0.25
0.00
0.00
0.25
0.50
0.75
1.00
method
Full data
MPRout
CHI
MR
VAR
RR

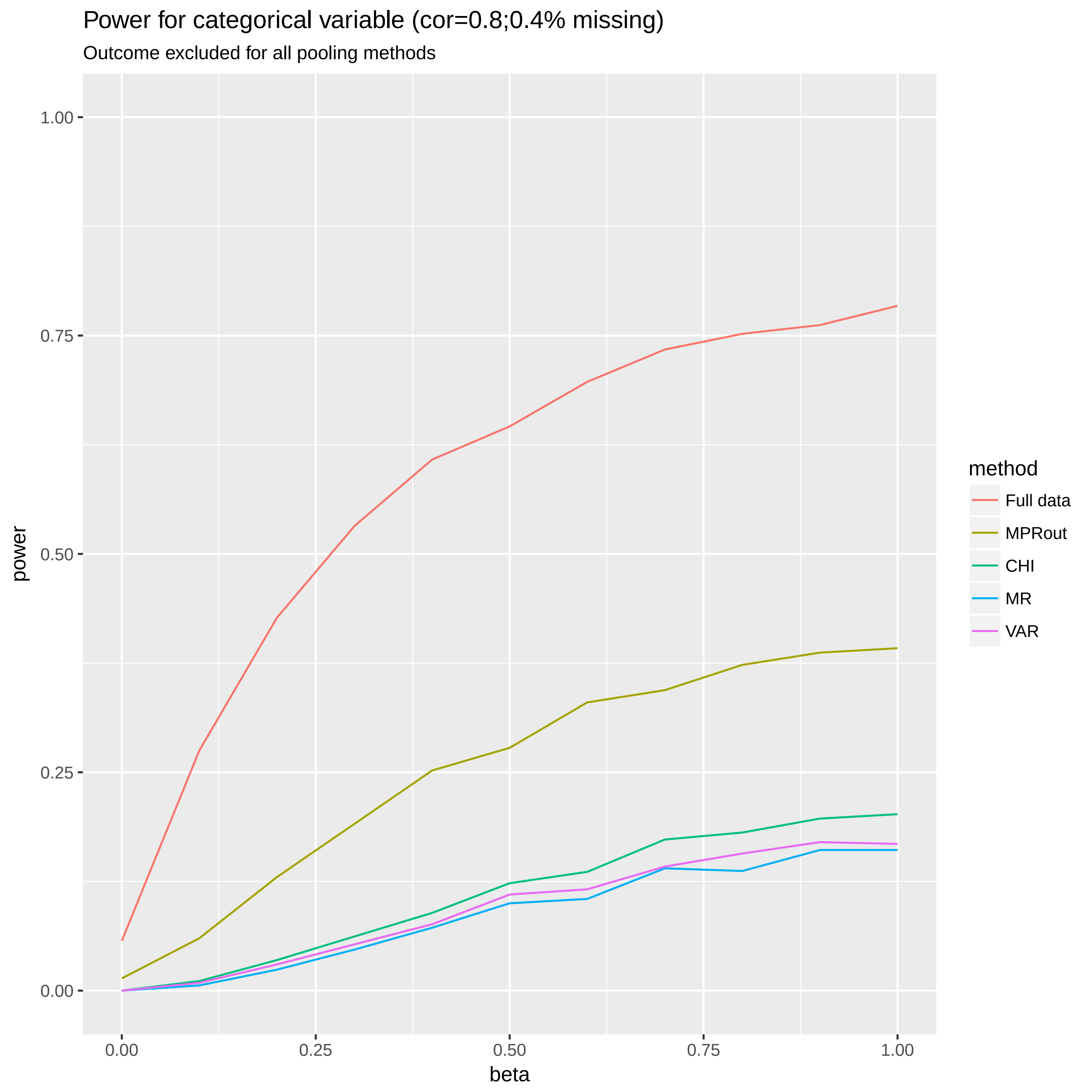
Power for categorical variable (cor=0.8;0.4% missing)
Outcome excluded for all pooling methods
power
1.00
0.75
0.50
0.25
0.00
beta
0.00
0.25
0.50
0.75
1.00
method
Full data
MPRout
CHI
MR
VAR

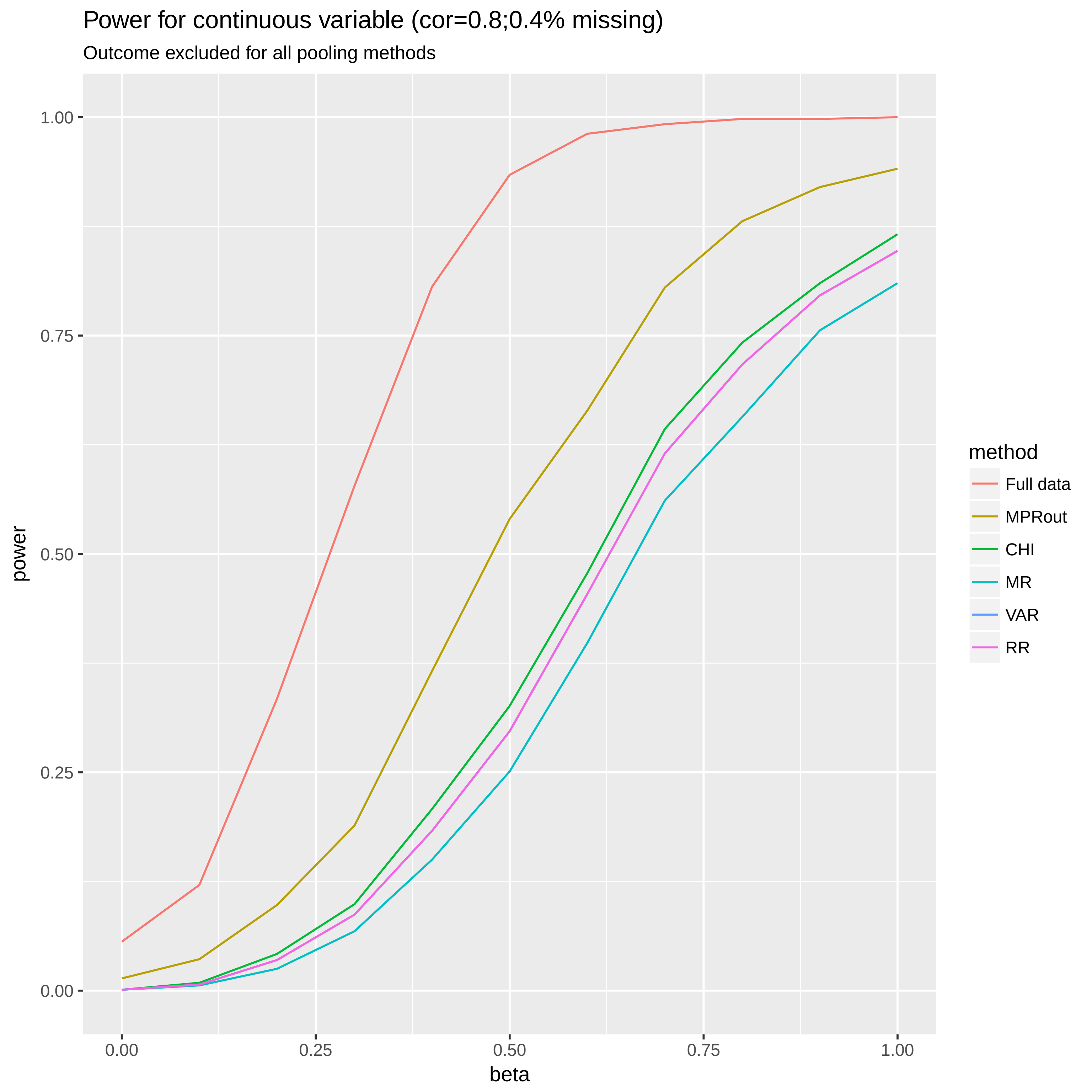

Power for continuous variable (cor=0.8;0.4% missing)
Outcome excluded for all pooling methods
power
1.00
0.75
0.50
0.25
0.00
beta
0.00
0.25
0.50
0.75
1.00
method
Full data
MPRout
CHI
MR
VAR
RR

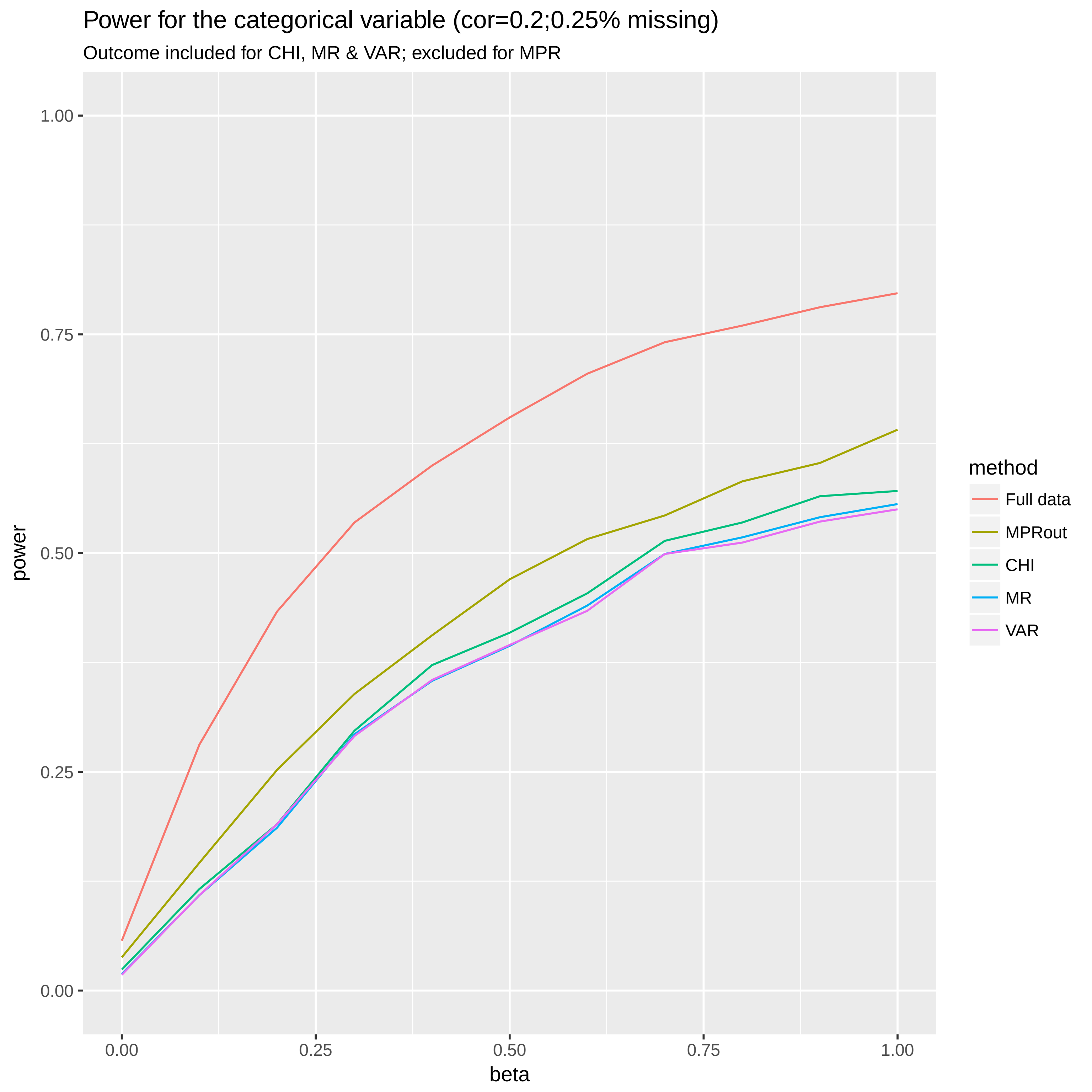

Power for the categorical variable (cor=0.2;0.25% missing)
Outcome included for CHI, MR & VAR; excluded for MPR
power
1.00
0.75
0.50
0.25
0.00
beta
0.00
0.25
0.50
0.75
1.00
method
Full data
MPRout
CHI
MR
VAR

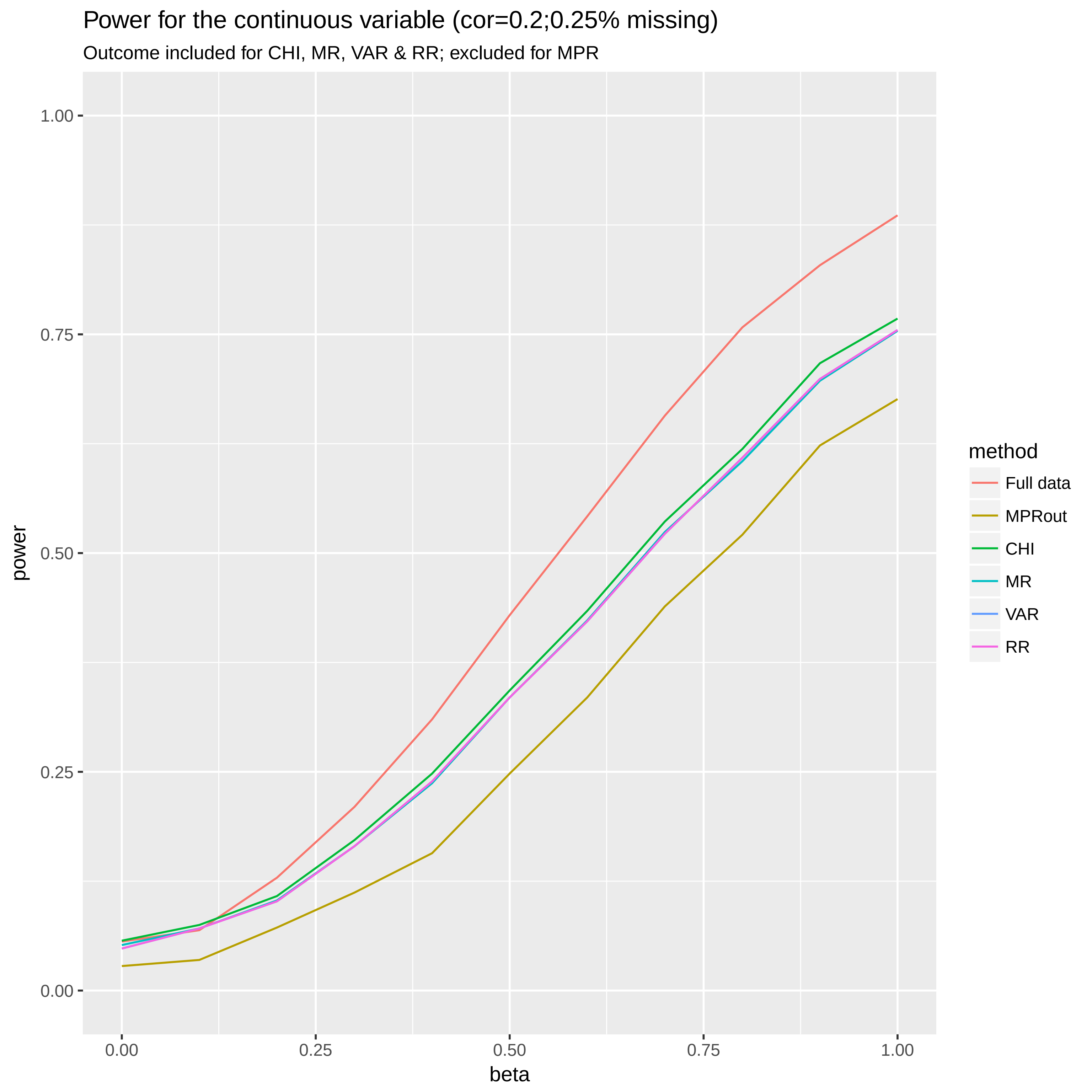
Power for the continuous variable (cor=0.2;0.25% missing)
Outcome included for CHI, MR, VAR & RR; excluded for MPR
power
1.00
0.75
0.50
0.25
0.00
beta
0.00
0.25
0.50
0.75
1.00
method
Full data
MPRout
CHI
MR
VAR
RR

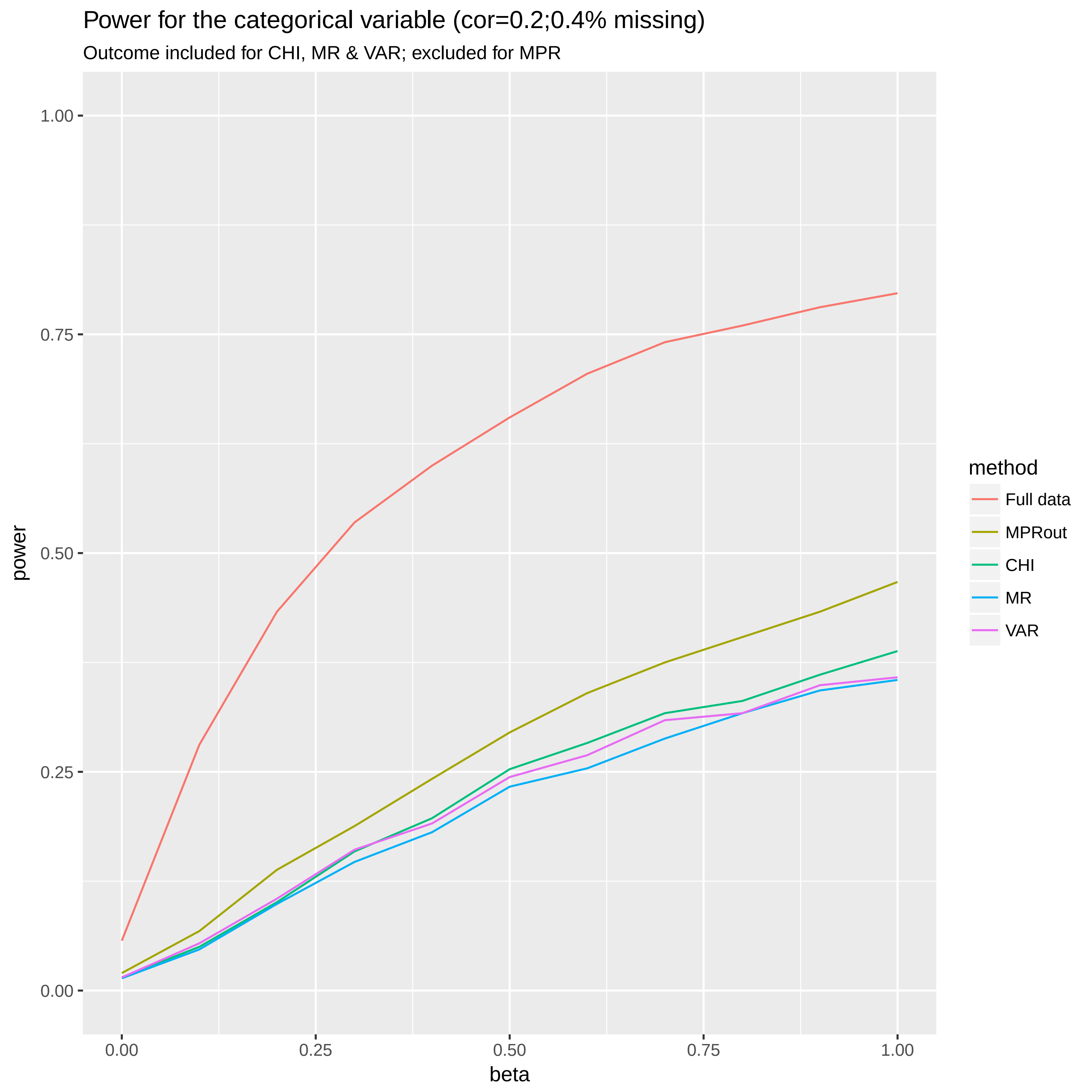

Power for the categorical variable (cor=0.2;0.4% missing)
Outcome included for CHI, MR & VAR; excluded for MPR
power
beta
0.00
0.25
0.50
0.75
1.00
method
Full data
MPRout
CHI
MR
VAR

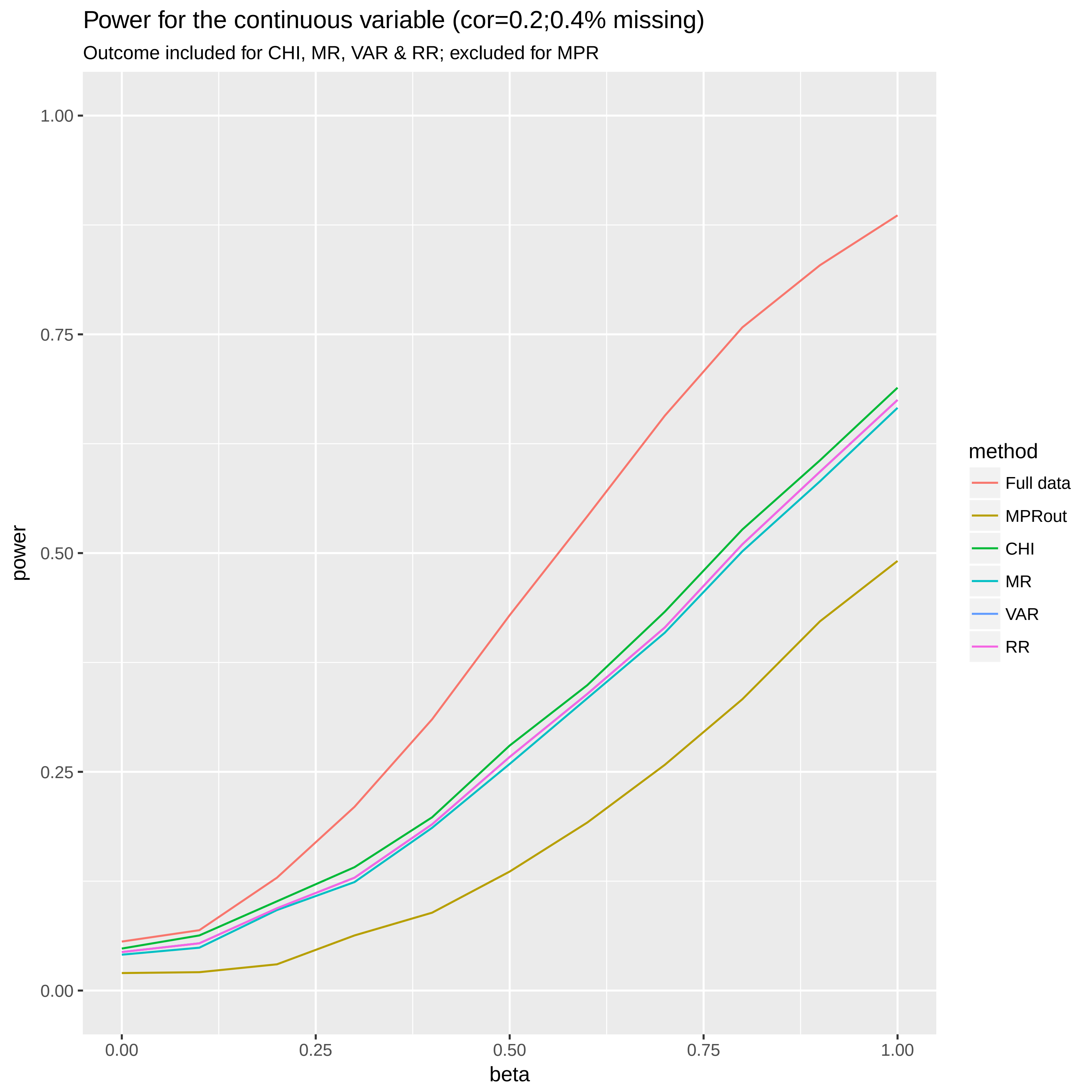
Power for the continuous variable (cor=0.2;0.4% missing)
Outcome included for CHI, MR, VAR & RR; excluded for MPR
power
beta
1.00
0.75
0.50
0.25
0.00
0.00
0.25
0.50
0.75
1.00
method
Full data
MPRout
CHI
MR
VAR
RR

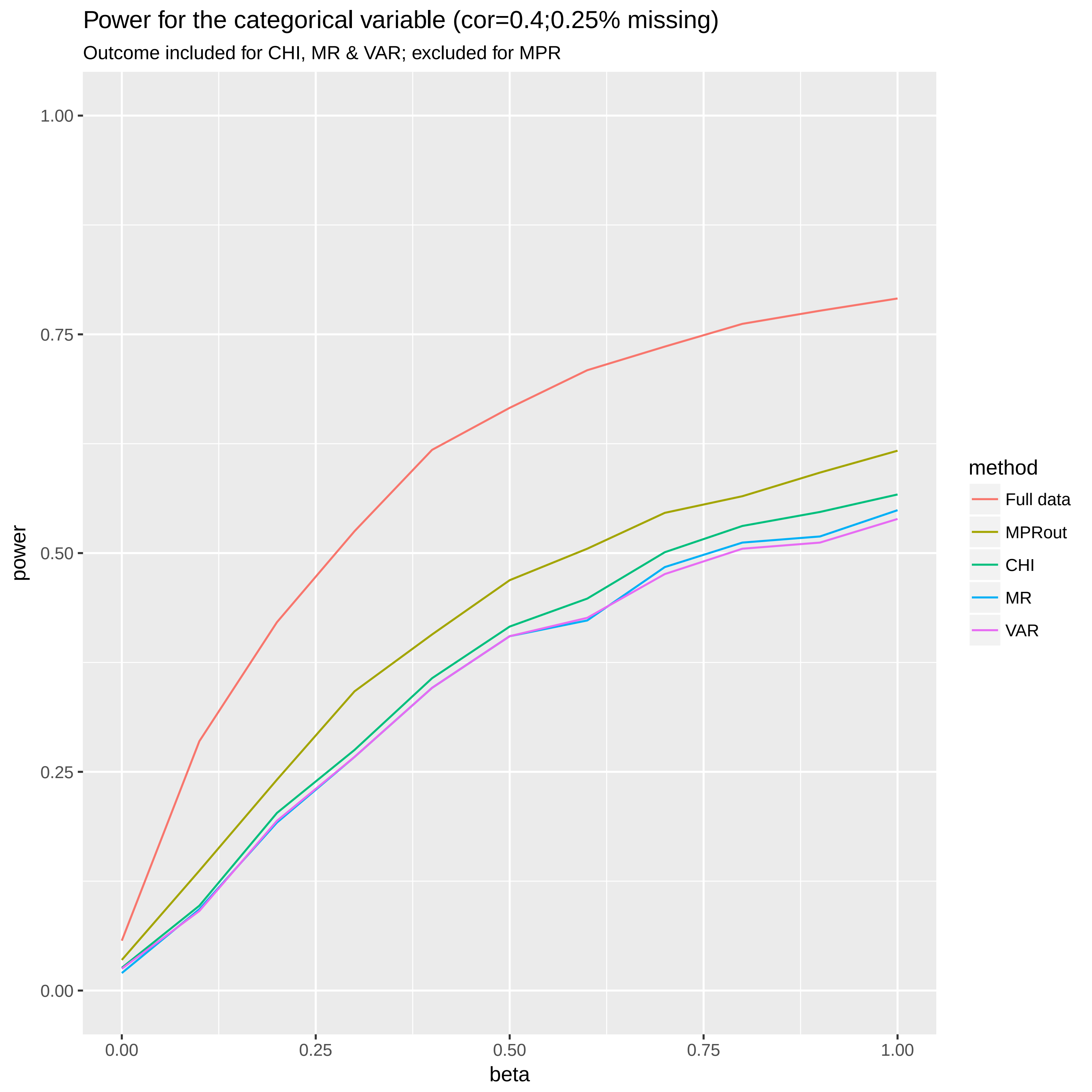
Power for the categorical variable (cor=0.4;0.25% missing)
Outcome included for CHI, MR & VAR; excluded for MPR
power
1.00
0.75
0.50
0.25
0.00
beta
0.00
0.25
0.50
0.75
1.00
method
Full data
MPRout
CHI
MR
VAR

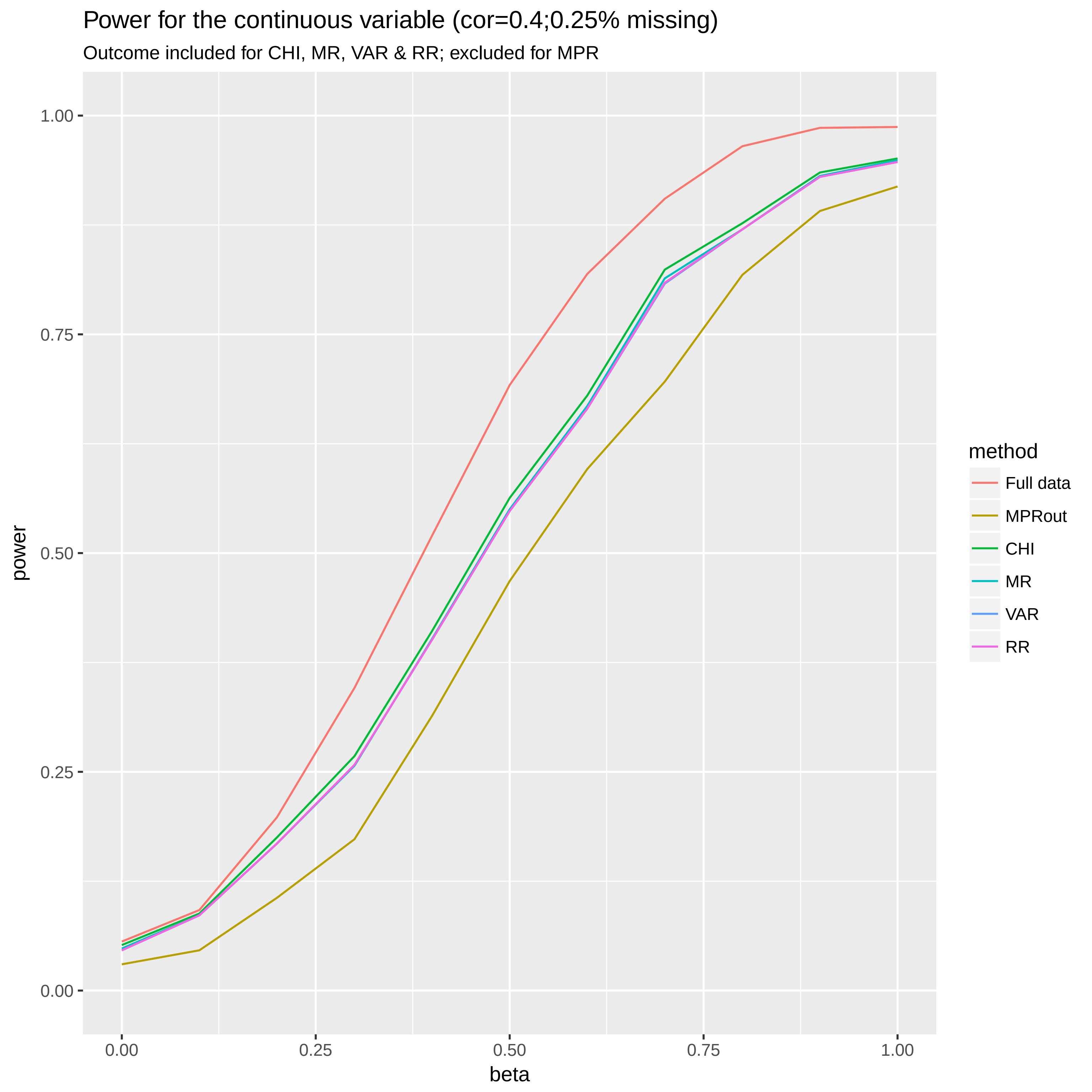

Power for the continuous variable (cor=0.4;0.25% missing)
Outcome included for CHI, MR, VAR & RR; excluded for MPR
power
1.00
0.75
0.50
0.25
0.00
beta
0.00
0.25
0.50
0.75
1.00
method
Full data
MPRout
CHI
MR
VAR
RR

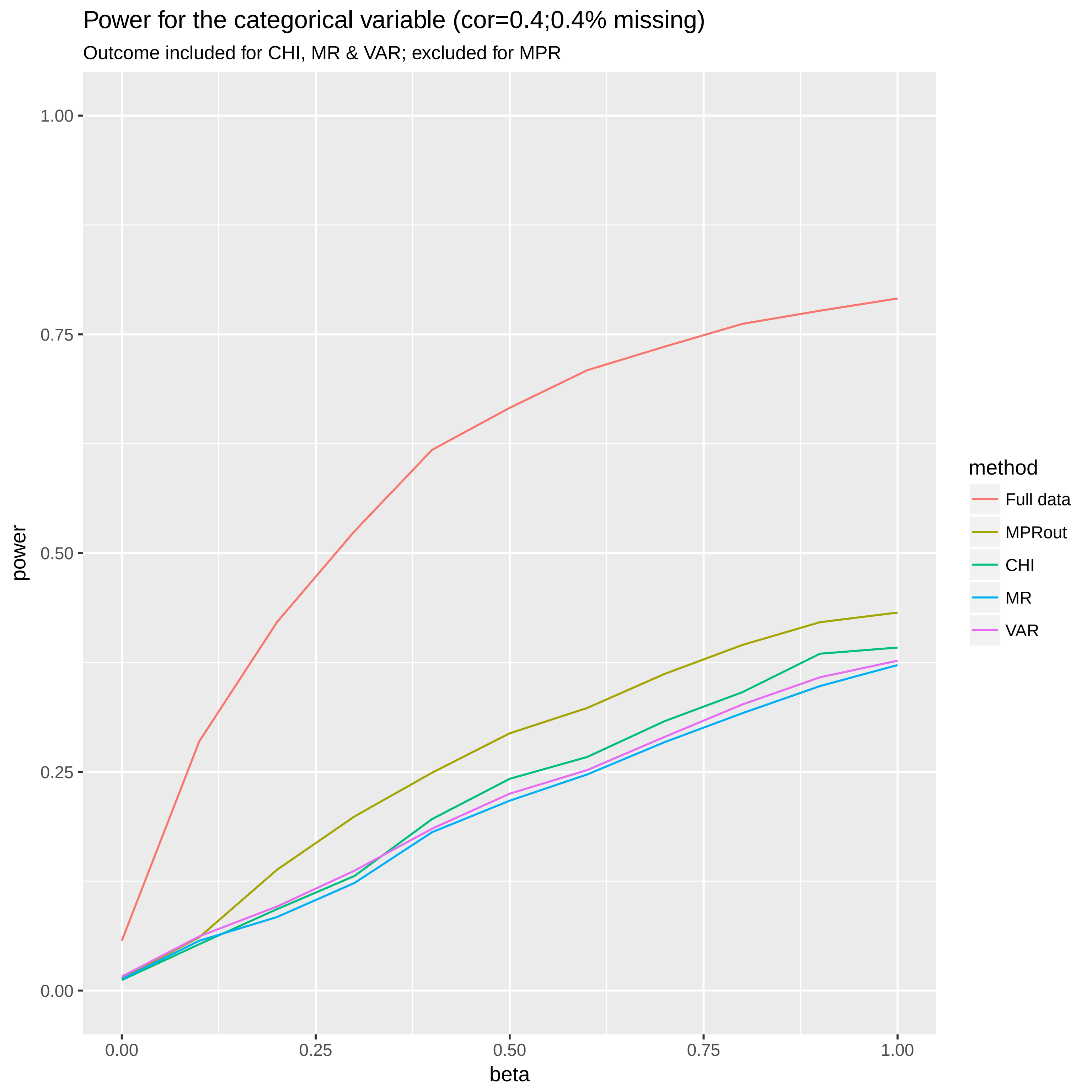

Power for the categorical variable (cor=0.4;0.4% missing)
Outcome included for CHI, MR & VAR; excluded for MPR
power
beta
1.00
0.75
0.50
0.25
0.00
0.00
0.25
0.50
0.75
1.00
method
Full data
MPRout
CHI
MR
VAR

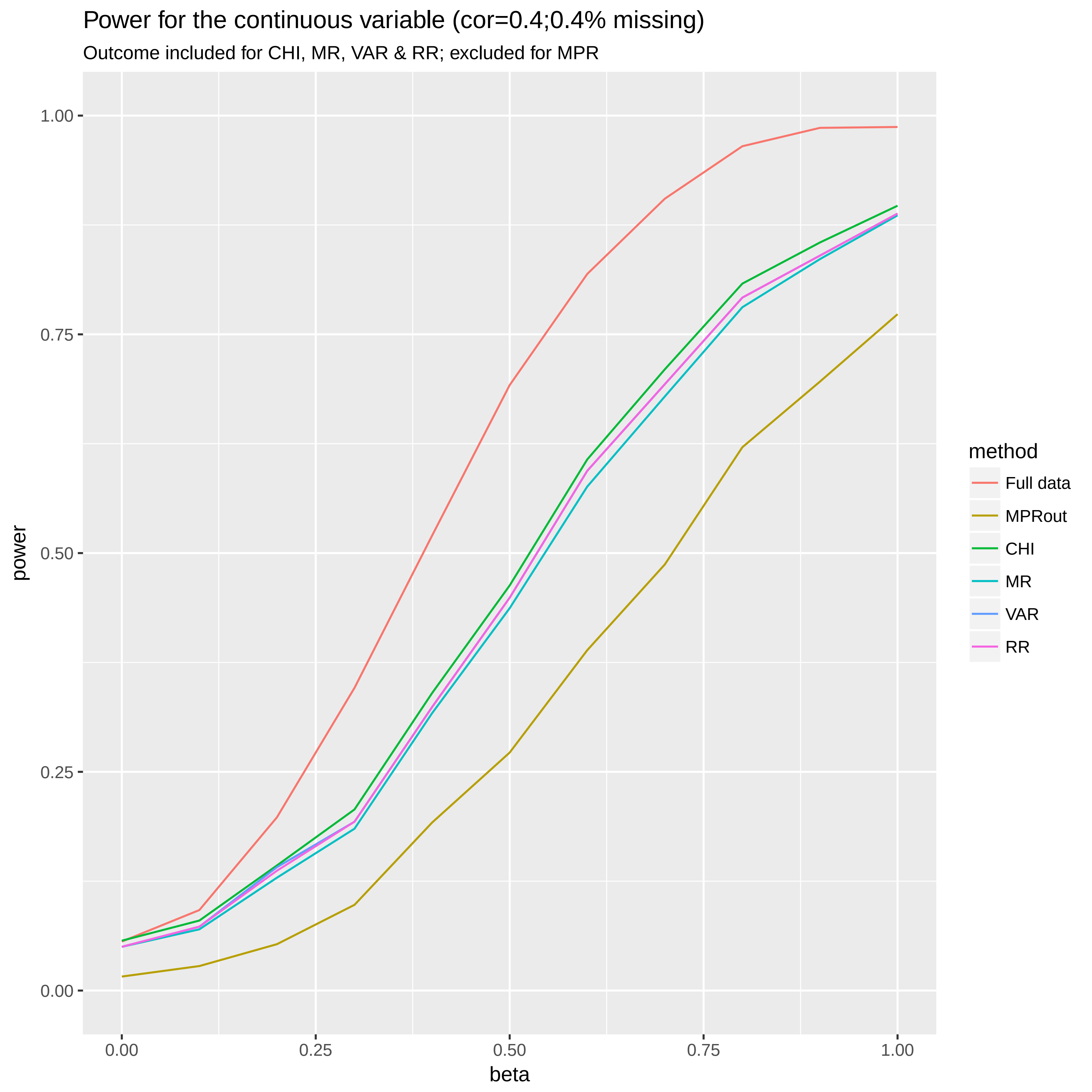
Power for the continuous variable (cor=0.4;0.4% missing)
Outcome included for CHI, MR, VAR & RR; excluded for MPR
power
1.00
0.75
0.50
0.25
0.00
beta
0.00
0.25
0.50
0.75
1.00
method
Full data
MPRout
CHI
MR
VAR
RR

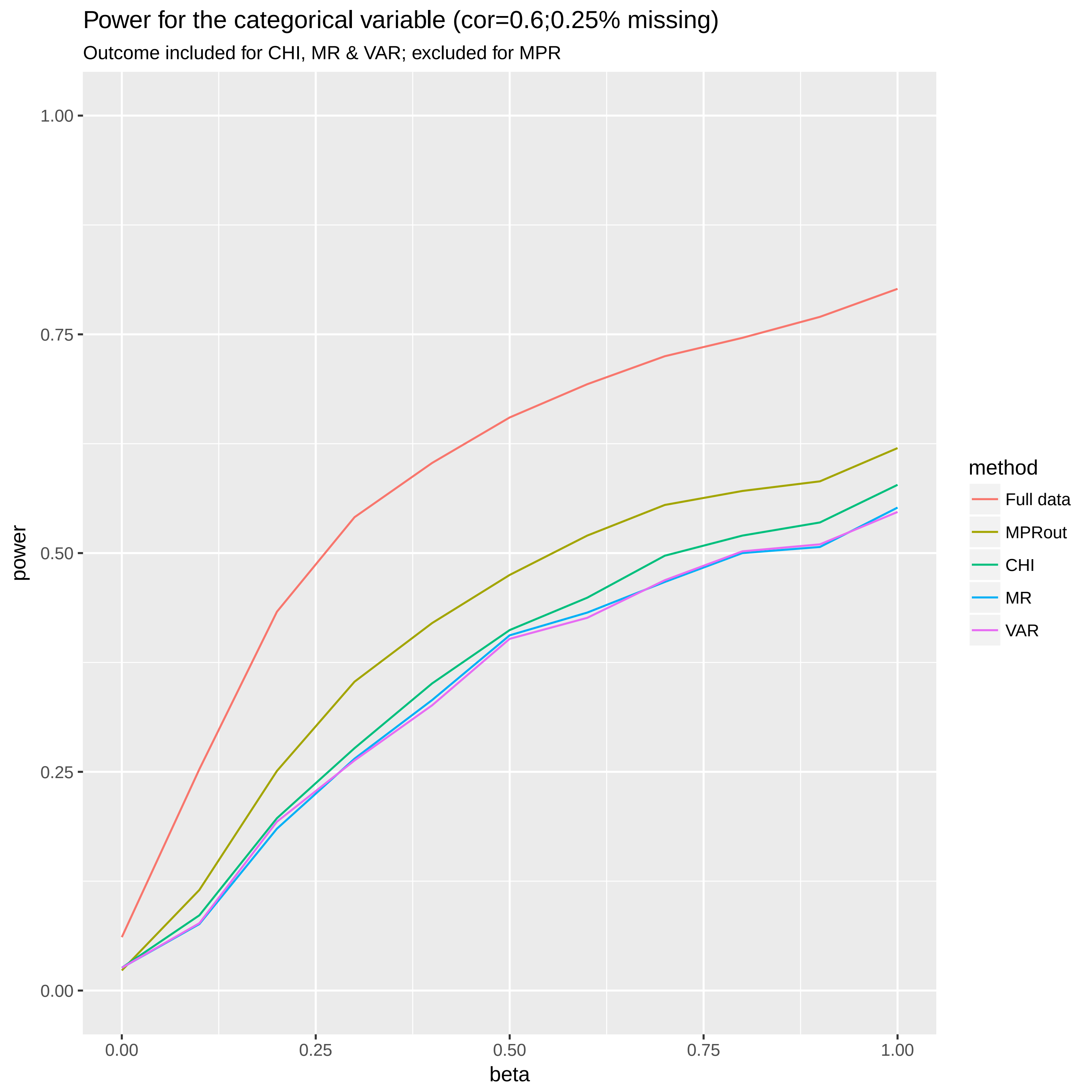

Power for the categorical variable (cor=0.6;0.25% missing)
Outcome included for CHI, MR & VAR; excluded for MPR
power
1.00
0.75
0.50
0.25
0.00
beta
0.00
0.25
0.50
0.75
1.00
method
Full data
MPRout
CHI
MR
VAR

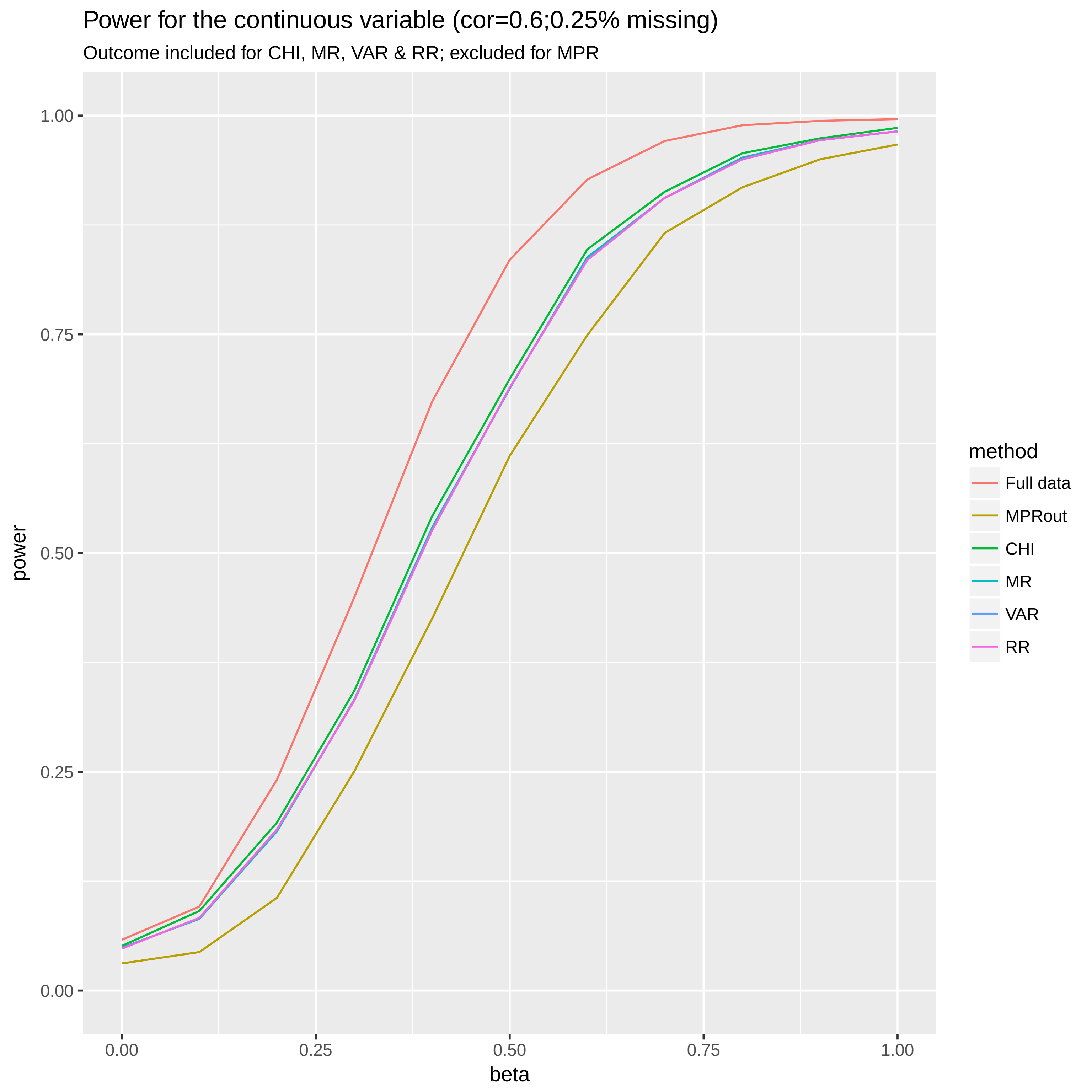

Power for the continuous variable (cor=0.6;0.25% missing)
Outcome included for CHI, MR, VAR & RR; excluded for MPR
power
1.00
0.75
0.50
0.25
0.00
0.00
0.25
0.50
0.75
1.00
beta
method
Full data
MPRout
CHI
MR
VAR
RR

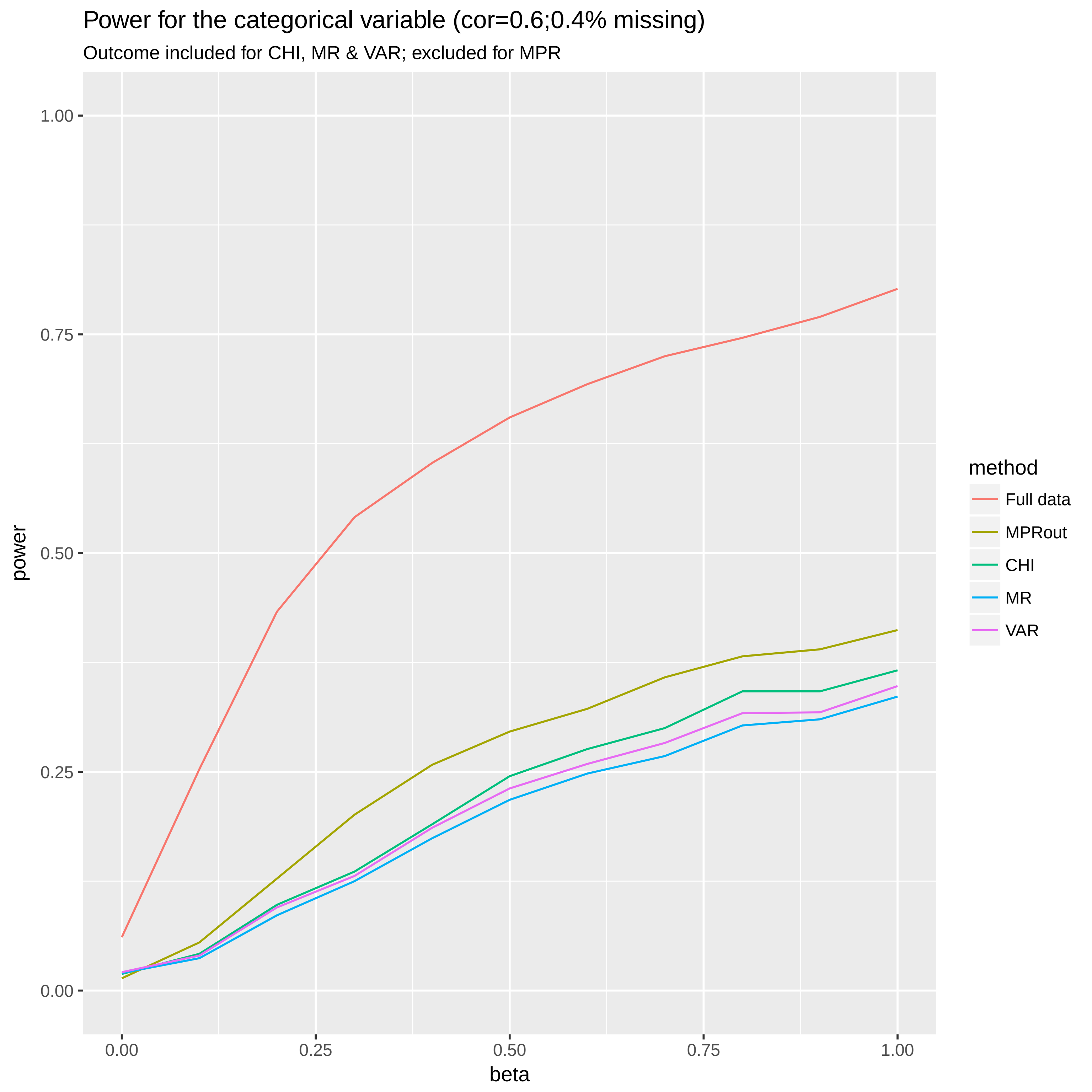

Power for the categorical variable (cor=0.6;0.4% missing)
Outcome included for CHI, MR & VAR; excluded for MPR
power
1.00
0.75
0.50
0.25
0.00
beta
0.00
0.25
0.50
0.75
1.00
method
Full data
MPRout
CHI
MR
VAR

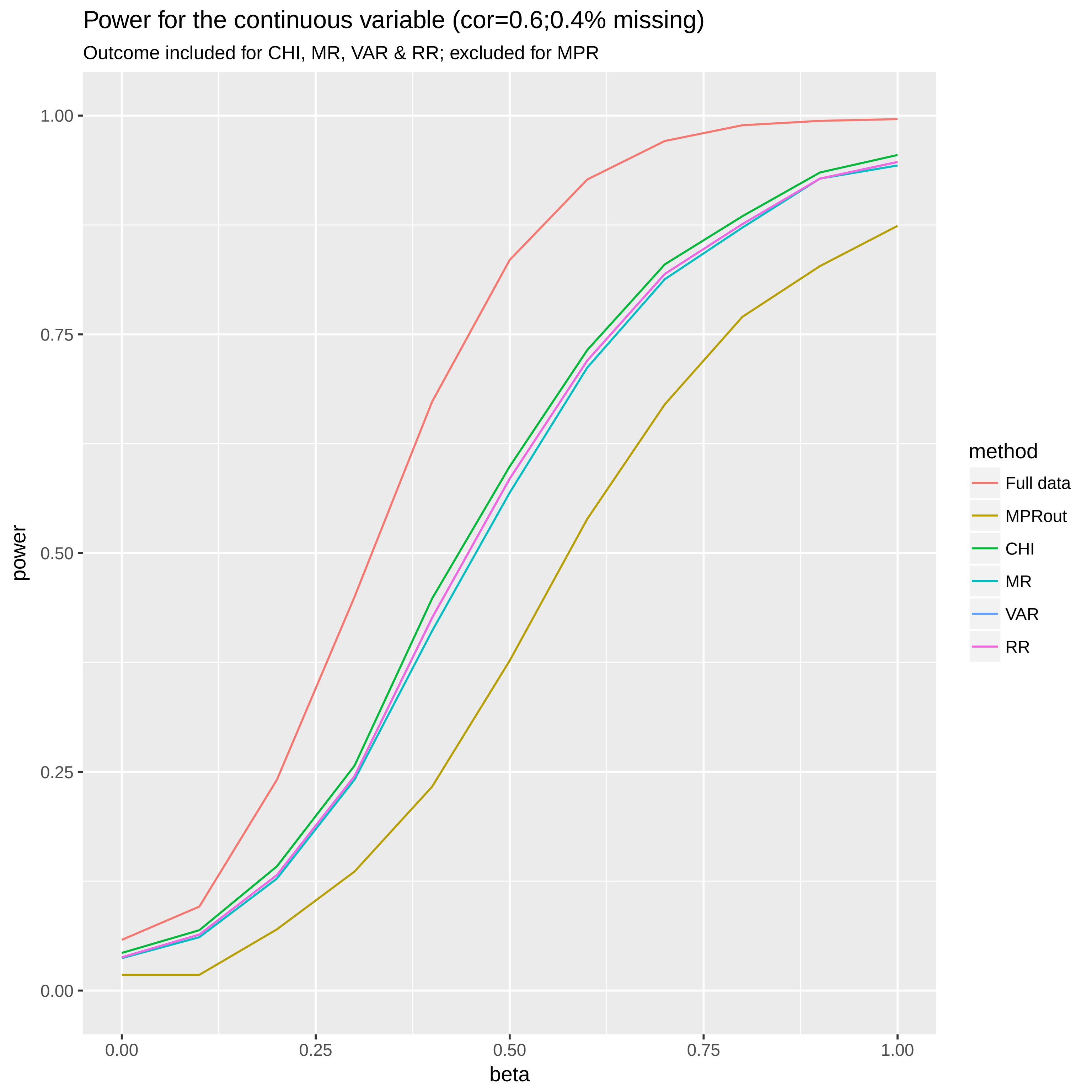
Power for the continuous variable (cor=0.6;0.4% missing)
Outcome included for CHI, MR, VAR & RR; excluded for MPR
power
beta
0.00
0.25
0.50
0.75
1.00
method
Full data
MPRout
CHI
MR
VAR
RR

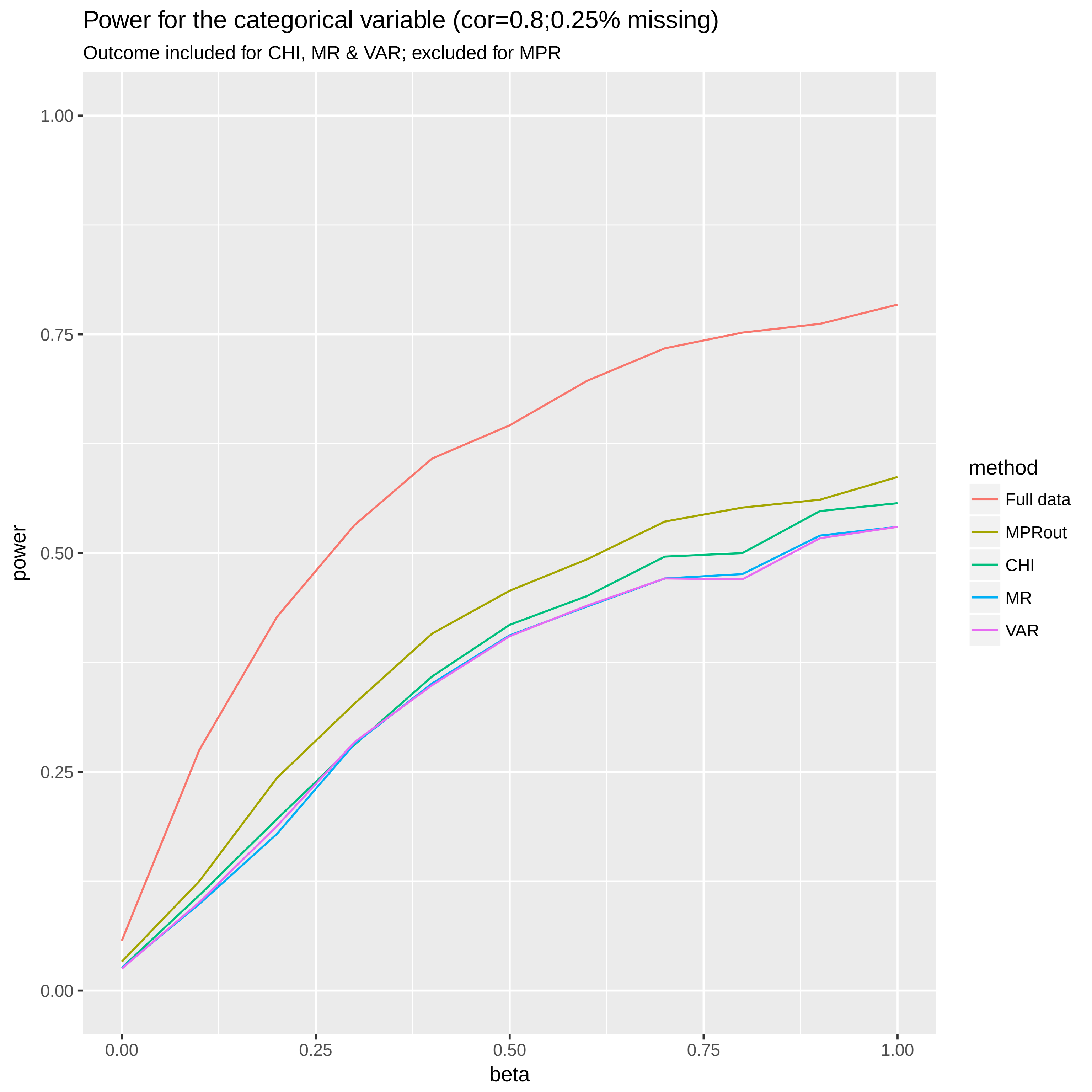
Power for the categorical variable (cor=0.8;0.25% missing)
Outcome included for CHI, MR & VAR; excluded for MPR
power
beta
1.00
0.75
0.50
0.25
0.00
0.00
0.25
0.50
0.75
1.00
method
Full data
MPRout
CHI
MR
VAR

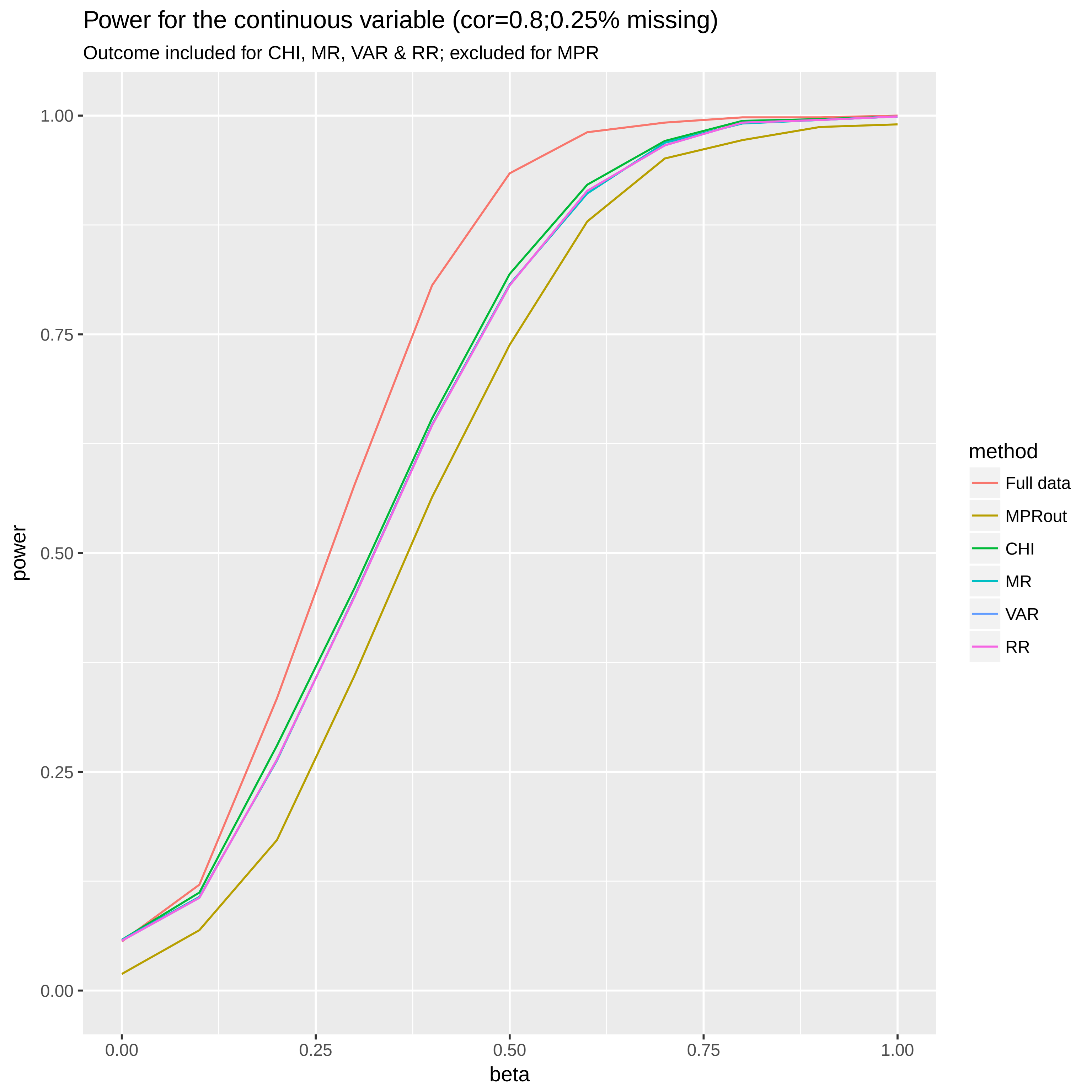
Power for the continuous variable (cor=0.8;0.25% missing)
Outcome included for CHI, MR, VAR & RR; excluded for MPR
power
1.00
0.75
0.50
0.25
0.00
beta
0.00
0.25
0.50
0.75
1.00
method
Full data
MPRout
CHI
MR
VAR
RR

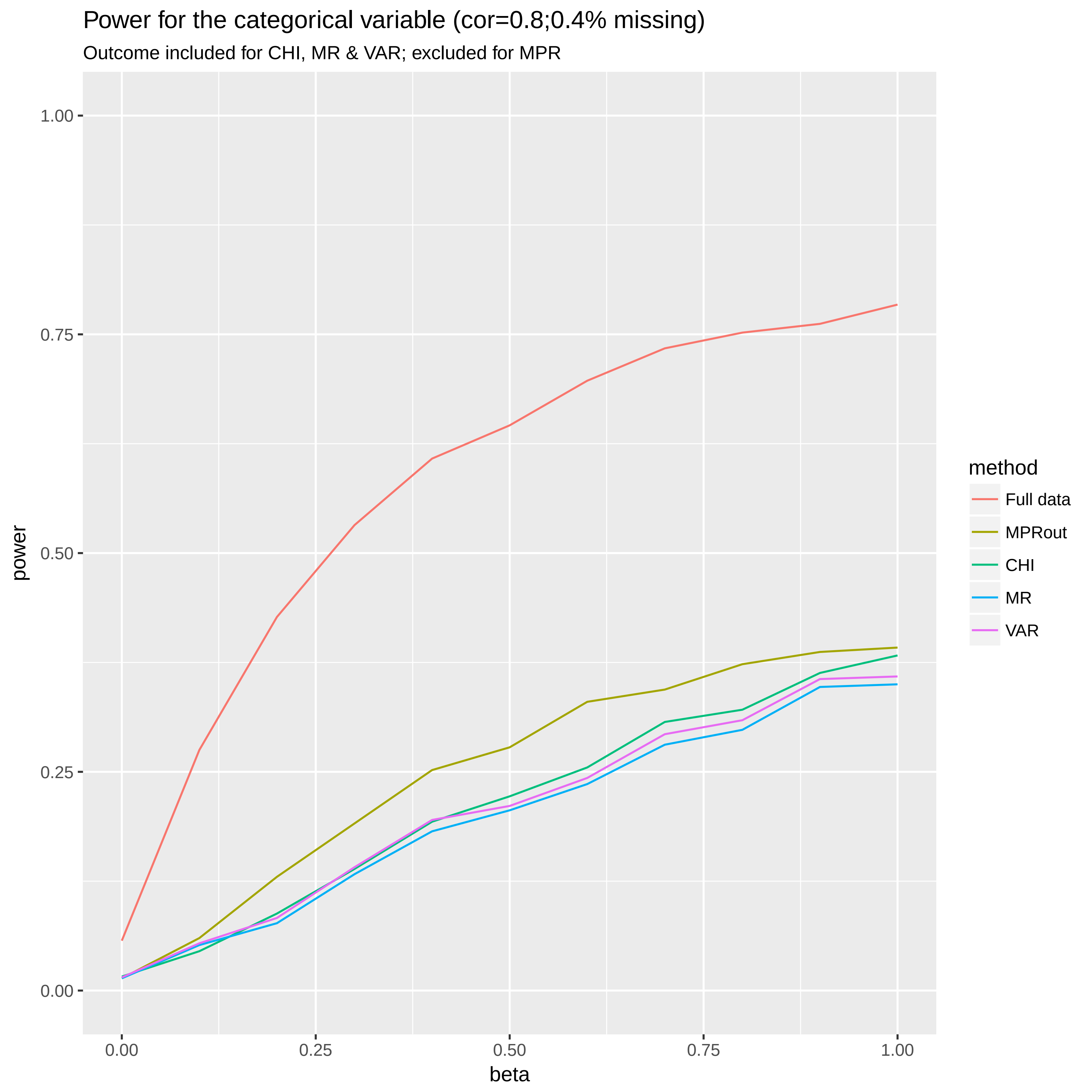

Power for the categorical variable (cor=0.8;0.4% missing)
Outcome included for CHI, MR & VAR; excluded for MPR
power
1.00
0.75
0.50
0.25
0.00
beta
0.00
0.25
0.50
0.75
1.00
method
Full data
MPRout
CHI
MR
VAR

Power for the continuous variable (cor=0.8;0.4% missing)

Outcome included for CHI, MR, VAR & RR; excluded for MPR

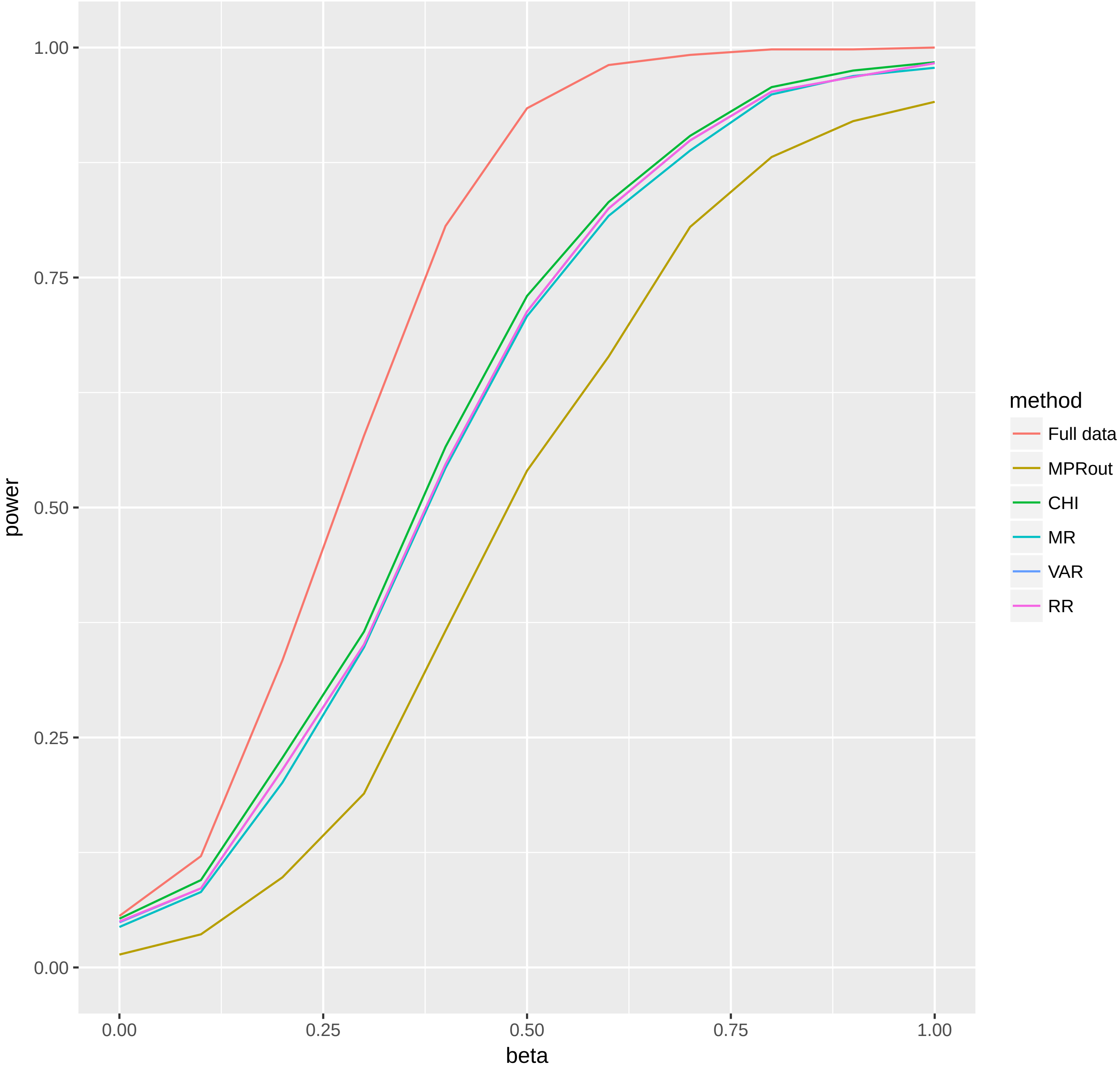

Supplement: Supplementary file 2 — Full overview of simulation results. (PDF 81 kb) [file 12874_2017_404_MOESM2_ESM.pdf]
